# Proteotranscriptomic Discrimination of Tumor and Normal Tissues in Renal Cell Carcinoma

**DOI:** 10.3390/ijms24054488

**Published:** 2023-02-24

**Authors:** Áron Bartha, Zsuzsanna Darula, Gyöngyi Munkácsy, Éva Klement, Péter Nyirády, Balázs Győrffy

**Affiliations:** 1 Cancer Biomarker Research Group, Institute of Enzymology, RCNS, H-1117 Budapest, Hungary; 2 National Laboratory for Drug Research and Development, RCNS, H-1117 Budapest, Hungary; 3 II. Department of Pediatrics, Semmelweis University, H-1094 Budapest, Hungary; 4 Single Cell Omics Advanced Core Facility, HCEMM, H-6728 Szeged, Hungary; 5 Laboratory of Proteomics Research, BRC, H-6726 Szeged, Hungary; 6 Department of Urology, Semmelweis University, H-1082 Budapest, Hungary; 7 Department of Bioinformatics, Semmelweis University, H-1094 Budapest, Hungary

**Keywords:** kidney cancer, proteomics, biomarker, diagnostics, mass spectrometry

## Abstract

Clear cell renal carcinoma is the most frequent type of kidney cancer, with an increasing incidence rate worldwide. In this research, we used a proteotranscriptomic approach to differentiate normal and tumor tissues in clear cell renal cell carcinoma (ccRCC). Using transcriptomic data of patients with malignant and paired normal tissue samples from gene array cohorts, we identified the top genes over-expressed in ccRCC. We collected surgically resected ccRCC specimens to further investigate the transcriptomic results on the proteome level. The differential protein abundance was evaluated using targeted mass spectrometry (MS). We assembled a database of 558 renal tissue samples from NCBI GEO and used these to uncover the top genes with higher expression in ccRCC. For protein level analysis 162 malignant and normal kidney tissue samples were acquired. The most consistently upregulated genes were IGFBP3, PLIN2, PLOD2, PFKP, VEGFA, and CCND1 (*p* < 10^−5^ for each gene). Mass spectrometry further validated the differential protein abundance of these genes (IGFBP3, *p* = 7.53 × 10^−18^; PLIN2, *p* = 3.9 × 10^−39^; PLOD2, *p* = 6.51 × 10^−36^; PFKP, *p* = 1.01 × 10^−47^; VEGFA, *p* = 1.40 × 10^−22^; CCND1, *p* = 1.04 × 10^−24^). We also identified those proteins which correlate with overall survival. Finally, a support vector machine-based classification algorithm using the protein-level data was set up. We used transcriptomic and proteomic data to identify a minimal panel of proteins highly specific for clear cell renal carcinoma tissues. The introduced gene panel could be used as a promising tool in the clinical setting.

## 1. Introduction

Clear cell renal carcinoma (ccRCC) is the malignant transformation of epithelial cells of the kidney and is the most frequent form of kidney tumors with approx. 70% of all kidney cancer cases [1]. In 2020, there were 431,288 new cases and 179,368 deaths from kidney and renal pelvis cancer worldwide [2]. Although the rate of new cases seems to rise, in the past decades, the mortality rates are stagnating in the US [3]. Risk factors of ccRCC include obesity, smoking, hypertension, older age, and male gender. Patients with a family history of ccRCC also have a higher risk of developing this disease [4].

Diagnosis of ccRCC is usually based on radiological imaging and tissue slide-based histopathological examination. Histopathological confirmation is essential before systematic therapy initiation. [4] Treatment of ccRCC can include surgery, percutaneous ablation [5], and targeted drugs including VEGF inhibitors [6] and mTOR inhibitors [7]. In the case of localized disease, surgical intervention is the first-line therapy, and depending on the size and stage, the intervention can range from partial to radical nephrectomy. If the tumor mass is relatively small, ablative techniques (such as cryo-, thermo-, or radio-ablation) are also available [5]. Patients with early-stage and lack of distant metastasis have more favorable survival rates than those with advanced disease [8]. Patients with advanced disease (stage IV) also require systemic therapy using mTOR inhibitors, VEGF inhibitors, or checkpoint inhibitors such as nivolumab, avelumab, pembrolizumab, ipilimumab, and interleukin 2 therapy [9].

MS was introduced almost half a century ago in endocrinology and toxicology for drug, steroid, and organic acid quantitation and got its main medical application in the widespread newborn screening [10,11]. Although the setup of MS-based diagnostic applications can be costly and complicated at the beginning, their versatility and reliability lead to new applications in clinical settings. In recent years, MS has been proven to be a comparatively cost-effective, precise, and quick analysis tool in microbial identification [12]. With the advent of proteomics and proteogenomics, MS-based techniques have an increasing role in cancer diagnostics, as well [13].

Uncovering a protein abundance-based panel specific to ccRCC could provide valuable support for the everyday clinical diagnostic and therapeutic decision-making process. Our study aimed to utilize large-scale transcriptomic studies to find genes showing higher expression in ccRCC. Then, by using our patient cohort with available proteomic and clinical data, we investigated the abundance of expressed proteins and the effect of these proteins on survival. By specifically focusing on markers with higher expression in tumor tissues, we aim to increase the specificity of our analysis to solidify future clinical application of the results.

## 2. Results

### 2.1. Database Setup

**Figure 1:**
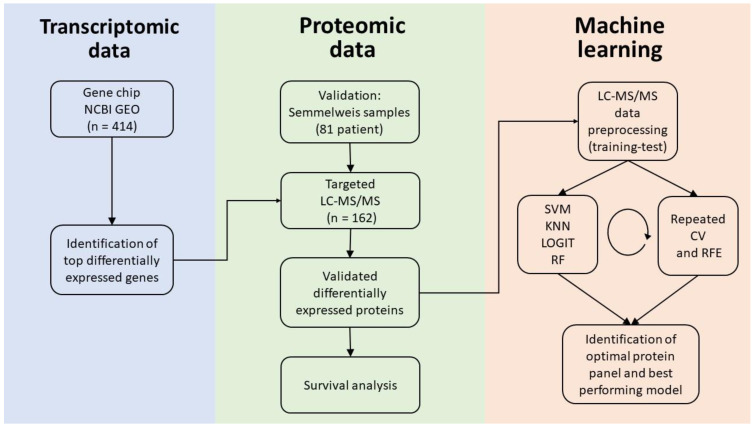
Analysis pipeline. Using gene chip data, we identified the top differentially expressed genes discriminating normal kidney tissue and ccRCC. We verified the identified gene panel using an independent validation cohort. We performed targeted LC-MS/MS to measure protein abundance for the selected top genes in the Semmelweis cohort. Using proteomic data, we established an optimal gene panel and the most accurate model for ccRCC detection. *CV: K-fold cross-validation, RFE: recursive feature elimination, KNN: k-nearest neighbors, RF: random forest, LOGIT: logistic regression, and SVM: support vector machines*.

Altogether, we included 23 GEO series which contained 715 samples. Out of these 715 samples, 277 were from normal kidney tissues, and 438 were from ccRCC. Out of the entire gene array database, 414 samples were paired samples (207 pairs), and we used the paired specimens for the identification of differentially expressed genes. The entire analysis pipeline is summarized in Figure 1. Patient characteristics are listed in Table 1.

### 2.2. Genes Over-Expressed in ccRCC

**Figure 2:**
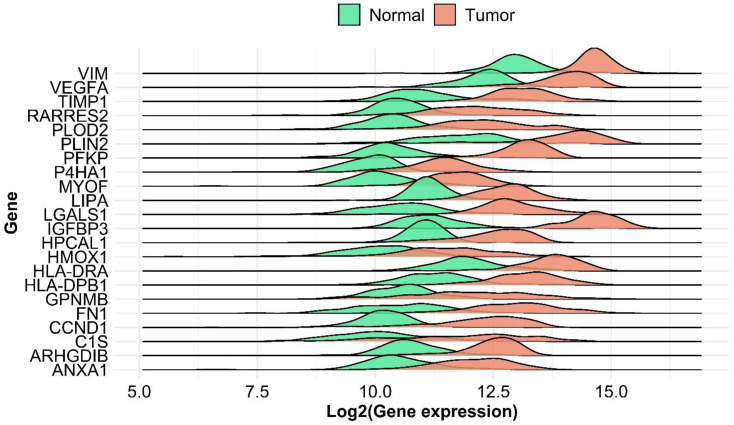
Differential gene expression of compared normal and ccRCC tumor samples from transcriptomic data. Ridge plots of differentially expressed genes shows the distribution of log2 expression values.

We uncovered significantly differentially expressed genes between paired ccRCC and adjacent normal tissues. IGFBP3 was found to be the most upregulated gene in tumor tissues (FC gene chip = 8.15, *p* = 5.88 × 10^−32^). The most significant genes include previously established molecular targets like VEGFA (FC gene chip = 3.02 *p* = 5.1 × 10^−31^) and CCND1 (FC gene chip = 4.12, *p* = 4.1 × 10^−31^). PLIN2 and PLOD2 also showed notable gene expression differences with FC values of 3.85 and 4.2 and adjusted *p* values of 3.09 × 10^−31^ and 5.24 × 10^−32^, respectively. The top differentially expressed genes are shown in Figure 2 and listed in detail in Supplementary Table S2.

### 2.3. Proteomic Analysis

**Figure 3:**
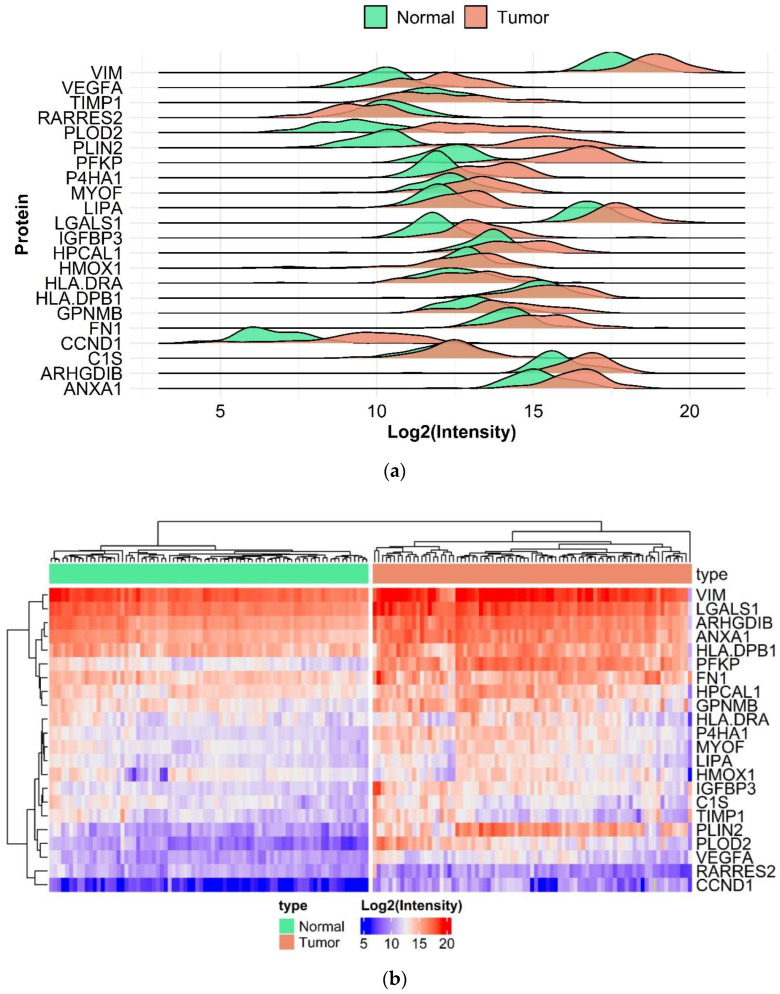
Differential protein abundances of compared normal and ccRCC tumor samples. Ridge plots of differentially expressed proteins shows the distribution of log2 intensity values (**a**). Heatmap of log2 intensity values (**b**).

**Figure 4:**
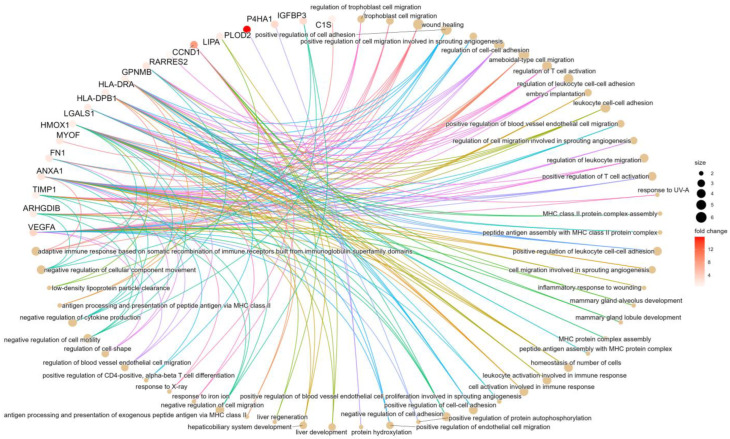
Gene ontology of the top genes. Gene ontology (GO) analysis of the strongest genes which discriminate normal kidney and ccRCC in all investigated cohorts. In the Gene-concept network plot (cnet plot) the linkages of genes and biological concepts are presented as a circular-shaped network. The color of the genes represents the FC values, and the size of the GO terms represents the associated genes.

Proteomic analysis was performed using 162 normal and malignant tissue samples. Of the complete list of the 31 selected genes from gene chip results, we were able to successfully measure 22 in the targeted LC-MS/MS. Top differentially expressed genes include PLIN2 (FC = 26.01, *p* = 3.9 × 10^−39^), PLOD2 (FC = 15.83, *p* = 6.51 × 10^−36^), PFKP (FC = 12.78, *p* = 1.01 × 10^−47^), IGFBP3 (FC = 3.04, *p* = 7.53 × 10^−18^), CCND1(FC = 7.9, *p* = 1.04 × 10^−24^) and VEGFA (FC = 3.5, *p* = 1.4 × 10^−22^) shown in Figure 3. Differential analysis between male and female patients resulted in no significant differences. Regression analysis of age and protein expression showed a significant result only in the case of IGFBP2, however, the adjusted R-squared value was 0.064. Thus, we can conclude that neither age nor gender can be considered as a covariate factor. Further results are provided in the Supplementary Table S4. Using the clusterProfiler R package, we performed an enrichment analysis; mostly enriched GO terms are connected to migration and adhesion. Results of the enrichment analysis are presented in Figure 4 and Supplemental Figure S1. Detailed results of the protein expression changes are also presented in Table 2. Intensities of the 22 best protein-specific peptides are presented in Supplemental Figure S2.

### 2.4. Survival Analysis Using Proteome-Level Data

**Figure 5:**
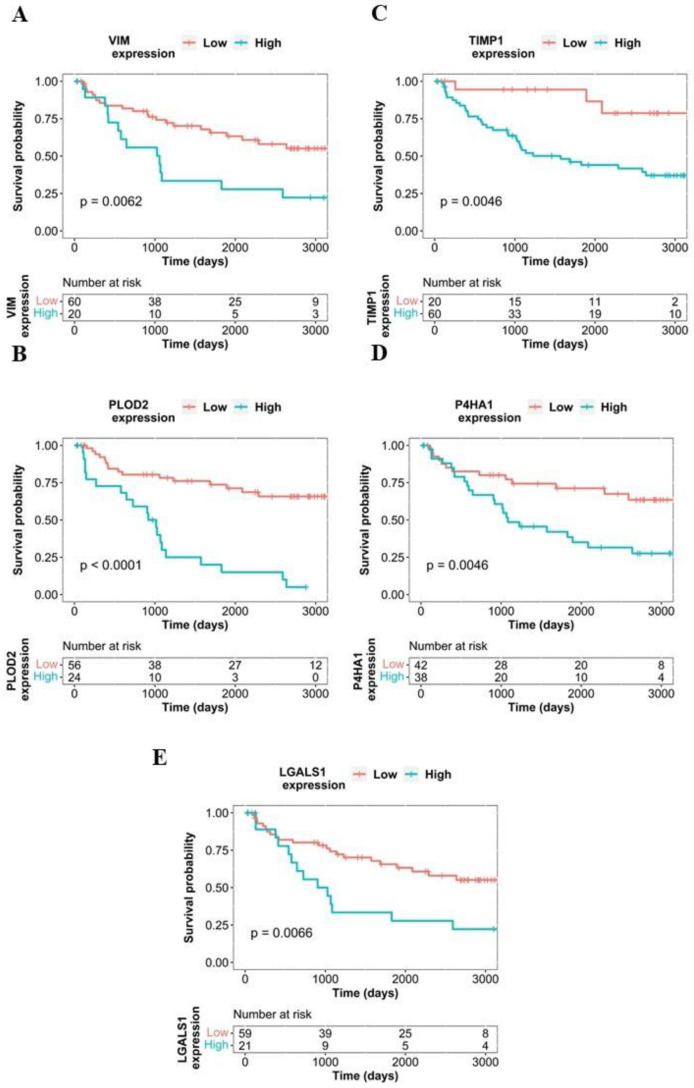
Kaplan–Meier plots of VIM (**A**), PLOD2 (**B**), TIMP1 (**C**), P4HA1 (**D**), LGALS1 (**E**), each protein shows a significant correlation with impaired overall survival.

To estimate the potential effects of protein expression on patient survival, we performed a survival analysis using all available proteins. Five out of the investigated proteins showed a correlation with survival. Patients with elevated expression of PLOD2 protein showed significantly worse overall survival compared to subjects with lower expression (*p* = 2.42 × 10^−7^, HR = 5.03). Overexpression of further proteins such as TIMP1 (*p* < 3 × 10^−2^, HR = 4.71), VIM (*p* < 3 × 10^−2^, HR = 2.49), LGALS1 (*p* < 3 × 10^−2^, HR = 2.47), and P4HA1 *p* < 3 × 10^−2^, HR = 2.6) also showed significant correlation with impaired overall survival. Kaplan–Meier curves of the best-performing proteins are shown in Figure 5; further results of survival analysis are presented in Supplemental Table S3 and as supplementary figures

### 2.5. Validation Using Data from CPTAC

**Figure 6:**
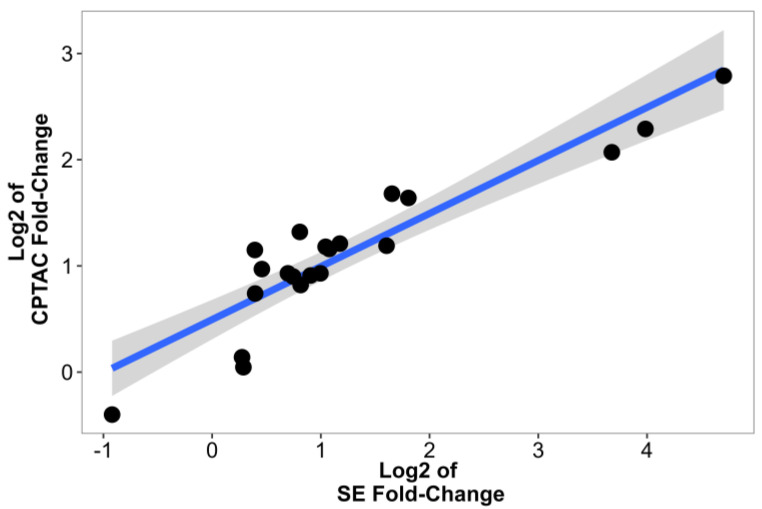
Correlation analysis of log-transformed CPTAC and SE Fold-change values. Each dot represents a FC value of a protein, we also added a trend line using a linear model.

To further support our analysis, we validated our results using CPTAC data from the study of Clark et al. [14]. Out of the 22 proteins identified by our current study, 21 were also available in the CPTAC dataset. The FC values between the two MS analyses had comparable results. Correlation analysis of the log2FC values of the CPTAC and SE cohorts resulted in a significant correlation (R = 0.91, *p* = 3.7 × 10^−9^, Figure 6). Top proteins identified, such as PLIN2 (FC = 6.92, *p* = 1.7 × 10^−33^), PLOD2 (FC = 4.89, *p* = 7.4 × 10^−33^), PFKP (FC = 4.2, *p* = 4.3 × 10^−56^), IGFBP3 (FC = 2.28, *p* = 2.1 × 10^−31^), and VEGFA (FC = 3.12, *p* = 3 × 10^−32^), had significant differences between normal kidney and ccRCC in the CPTAC study. Further results are displayed in Table 3.

### 2.6. ccRCC-Specific Model Creation

MS-based protein abundance data of the investigated proteins in the 162 patient samples were used for establishing the most robust classification algorithm. We investigated multiple machine learning methods (including k-nearest neighbors, random forest, logistic regression, and support vector machines) to build a model which can differentiate between normal and malignant kidney tissues. For the proper estimation of the optimal gene panel, we performed recursive feature elimination. Of the four methods, SVM delivered the best performance in both test and training cohorts using nine proteins as input. SVM was able to identify tumor tissues from MS quantification data with a classification accuracy of 0.98 in the test set (Kappa = 0.95, sensitivity = 0.95, specificity = 1). Results of all four methods (SVM, k-nearest neighbors, random forest, and logistic regression) in both training and test sets are displayed in Table 4; the list of optimal genes is provided in Table 5, and the accuracy of each method with different gene panels is presented in Supplemental Figure S3.

## 3. Discussion

Current clinical diagnostics of cancer rely mainly on pathological examination using tissue slide staining or immune histochemistry. The importance of tissue inspection is undoubted. However, with the increasing burden of workload in pathological diagnostics, the need for further potent diagnostic possibilities and tools capable to provide sufficient pathological decision support is necessary. While transcriptome-based methods are useful for this purpose, several studies with promising results were published recently in the proteome field as well. Establishing proteins with differential abundance in malignant samples compared to healthy tissues can provide valuable information in diagnostics and therapeutic target identification. For example, a breast cancer study comparing malignant breast cancer samples to adjacent normal samples using MS identified a novel luminal subtype [15]. A comparison of normal prostate and prostate adenocarcinoma samples was performed to identify a new prognostic biomarker [16].

Like other cancer types, early surgical intervention is the best solution for total recovery in ccRCC as well. Especially in the early stages, when the disease is localized, partial or radical nephrectomy is the most frequently performed treatment option [5]. In the present study, by using transcriptomic data, we uncovered genes with higher expression in ccRCC, and we then developed an algorithm capable of identifying ccRCC tissues with accuracy high enough for future clinical application. We focused on genes having higher expression in the tumor tissues. By using targeted MS data of the selected proteins, our algorithm can differentiate between normal and malignant tissues and could provide valuable decision support during the pathological diagnostic process.

The final discriminative algorithm is based on the differential expression of nine proteins. Of these, VEGFA and CCND1 are well-known cancer biomarkers. VEGFA (vascular endothelial growth factor A) is used as a target molecule in ccRCC treatment [6]. CCND1 (cyclin D1), a member of the cyclin family, acts as a regulator of cyclin-dependent kinases (CDKs). CDK inhibitors are widely used in the treatment of breast cancer [17]. PLOD2 (procollagen-lysin 2-oxoglutarate 5-dioxygenase) has a role in the maintenance of intermolecular collagen cross-links [18]. The aberrant function of PLOD2 might have a role in ovarian cancer [18] and gastric cancer progression [19]. PFKP (phosphofructokinase platelet isoform) is responsible for one of the early steps of glycolysis [20]. It might also have a crucial part in metabolic reprogramming in multiple cancer types like breast cancer [21] and non-small cell lung cancer [22]. IGFBP3 (insulin-like growth factor binding protein 3) acts as a carrier protein of several types of IGF molecules, and it is related to cell growth and differentiation [23]. IGFBP3 has been shown to be important in the development of colorectal and breast cancer [23,24]. PLIN2 (perilipin 2) is a member of the perilipin family and takes part in the formation of intracellular lipid storage droplets in multiple tissue types [25]. It has been connected to the development of atherosclerosis [26] but it has relevance in cancer initiation and progression as well [25]. Using Western blot technique, an earlier study has proposed PLIN2 as a potential plasma biomarker in ccRCC [27]. As both IGFP3 and PLIN2 can be detected in the plasma, we hypothesize that they could also serve as potential diagnostic biomarkers of ccRCC. Using our current knowledge, however, we lack any robust evidence for our hypothesis.

By survival analysis, we identified five proteins with a high expression which correlates with poor survival outcomes. Out of these five, PLOD2, VIM, and P4HA1 are also highlighted by our model. Both PLOD2 and P4HA1 are enzymes involved in collagen-related pathways and proved to be a biomarker of epithelial-to-mesenchymal transition (EMT) in multiple types of cancers [28,29]. While vimentin acts as an important structural protein and a known marker of EMT, overexpression of these proteins in patients with poor survival outcomes implies their involvement in EMT and metastasis formation in renal cell clear carcinoma.

We must note an important limitation of our approach. Although transcriptome-based examinations can provide valuable input of new potential biomarkers, due to mechanisms like alternative splicing, mutations, and post-translational modifications, RNA expression only moderately correlates with protein expression [30]. A further limitation of our model is the incapability of tumor stage estimation, as staging is usually based on imaging, pathological examination, and further clinical characteristics.

In conclusion, we used a database of renal samples of paired normal and tumor tissues to identify biomarkers differentiating renal clear cell cancer (ccRCC) and normal kidney tissues. With a support vector machine-based machine learning algorithm using nine genes, we set up a model which can differentiate between normal and malignant ccRCC tissues using proteomic data. Finally, a set of proteins showed a significant correlation with poor survival outcomes and might serve as potential biomarkers of progression.

## 4. Materials and Methods

### 4.1. Gene Chip Database Comprising Normal and Tumor Tissues

To set up the gene chip cohort, we searched the NCBI GEO repository (https://www.ncbi.nlm.nih.gov/geo/, accessed on 21 January 2021) for potential ccRCC and normal specimens using keywords “ccRCC” AND “normal” OR “GPL570” OR “GPL571” OR “GPL96”. Only those datasets involved contained normal tissues adjacent to tumors from HGU133, HGU133A_2, and HGU133A platforms. We filtered the datasets to exclude xenograft experiments, pooled samples, and cell line studies. Samples with insufficient description, nonexistent raw data, and repeatedly published data with distinct identifiers have been removed. To achieve this, the expression of the first twenty genes was determined, and samples with identical values were identified. In each case, the first published version was retained in the dataset. After the manual selection, the remaining samples were normalized using the MAS5 algorithm by utilizing the Affy Bioconductor library [31]. Finally, a second scaling normalization was executed to set the mean expression on each array to 1000. JetSet correction and annotation package was used to pick the proper probe set for each gene [32].

### 4.2. Determining Differentially Expressed Genes

Data processing and analysis were performed in R version 4.1.0 (https://www.r-project.org, accessed on 6 June 2021). Wilcoxon test was used to compare the tumorous and adjacent normal samples. Genes showing significant differences according to the Wilcoxon test (*p* < 0.01) have been selected and ranked based on their fold-change values (FC). The Benjamini–Hochberg method was used for *p*-value adjustment. Finally, the top 31 genes with an FC over two were selected for further investigation.

### 4.3. Ethics Statement

ccRCC samples were collected at the Department of Urology of the Semmelweis University. An institutional ethical review board approved the study under the number ID 7852-5/2014/EKU by Semmelweis University Regional and Institutional Committee of Science and Research Ethics. All subjects were treated under the tenets of the Declaration of Helsinki and written informed consents were obtained before sample collection.

### 4.4. Sample Collection

Clear cell renal carcinoma and adjacent normal samples were collected during surgical resection, and the tissue samples were stored immediately at −80 °C.

Protein isolation was performed using the AllPrep DNA/RNA/Protein Mini Kit by the manufacturer’s protocol using 30 mg of tissue samples.

### 4.5. Targeted Liquid Chromatography Coupled Tandem Mass Spectrometry (LC-MS/MS) Analysis

The expression of selected target proteins was verified by targeted LC/MS-MS. After isolation, protein samples were stored in guanidine isothiocyanate and stored at −80 °C. For targeted quantification, we used stable isotope labeled (SIL) peptides (1–5 respectively for each protein, labeled at Arg:13C6;15N4, Lys:13C6;15N2); the peptide sequences of the 75 SIL peptides are listed in Supplementary Table S1. Protein concentration was determined by the bicinchoninic acid (BCA) test. Samples were reduced by dithiothreitol (DTT) and alkylated using iodoacetamide followed by protein precipitation; then, samples were re-dissolved in 5% SDS/50 mM ammonium-bicarbonate for the BCA test. Sample volumes representing 50 μg protein content were digested by trypsin according to the S-trap protocol (https://files.protifi.com/protocols/s-trap-mini-long-4-1.pdf, accessed on 9 January 2023).

LC-MS/MS analysis was performed using an ACQUITY UPLC M-Class system (Waters, Milford, MA, USA) with HPLC coupled to an Orbitrap Fusion Lumos Tribrid (Thermo Fisher Scientific, Waltham, MA, USA) mass spectrometer on the mixture of the protein digests spiked with the mixture of the SIL peptides. Samples were loaded onto a trap column, ACQUITY UPLC M-Class Symmetry C18 Trap (100 Å, 5 µm, 180 µm × 20 mm, 2G, V/M); the sample loading time was 5 min; the flow rate was 5 µL/min, and separation was performed on an ACQUITY UPLC M-Class Peptide BEH C18 (130 Å, 1.7 µm, 75 µm × 250 mm) column with a flow rate of 400 nL/min. MS data acquisition was performed in an internal standard triggered parallel reaction monitoring fashion [33], where the presence of the corresponding SIL peptides, verified by their expected retention time and MS2 fragmentation pattern, triggers data acquisition of the targeted peptides with high sensitivity and resolution. MS signal intensities of the SIL peptides were between 1–5 × 10^7^. Raw MS data were analyzed using the Skyline software and the MSstats statistical analysis tool. During the data processing steps, we performed the inbuilt normalization steps of the MSstats software package, which includes median polishing and log2 transformation.

### 4.6. Statistical and Functional Analysis, Data Visualization

T-test was used to compare the log2 transformed protein intensity values between the tumorous and adjacent normal samples. In order to examine if any of the gene candidates are affected by covariates, we performed a *t*-test to see if any of the proteins show differential expression between male and female patients. To examine age as a covariate factor, we performed regression analysis to see if any of the examined proteins are influenced by age. Functional analysis was performed using the clusterProfiler R package [34]. For each protein, we performed Cox proportional hazard regression analysis. To estimate the best cutoff value for each protein, we examined each possible cutoff values between the lower and the upper quartiles; these cutoff values have been used for Kaplan–Meier plot visualization. The Benjamini–Hochberg method was used for *p*-value adjustment. For survival analysis, we used the survminer and survival R packages. Further visualization has been done using the R packages ggplot2 [35], ComplexHeatmap [36], and ggrepel (https://cran.r-project.org/web/packages/ggrepel/index.html, accessed on 13 December 2022).

### 4.7. Building a Model for ccRCC Detection

Using the results of the targeted LC/MS-MS log2 intensity values, we tried four supervised AI methods, k-nearest neighbors (KNN), random forest (RF), logistic regression (LOGIT), and support vector machines (SVM), to set up the most accurate model for cancer detection. The data matrix from MS data was the input for the classification model, and we used the “caret” R package for data preparation and model establishment [37,38]. From all available patients with MS data, we had to remove one patient due to a missing value. The entire cohort was split into training and test cohorts with a ratio of 0.7:0.3. Repeated K-fold cross-validation was used for training cohort resampling with 10 folds and 5 repeats. Within the resampling mechanism, we performed recursive feature elimination to specify the ideal number of used genes for each of the SVM, KNN, LOGIT, and RF algorithms. Model prediction capability was validated using the test set. The caret package’s built-in methods were used to determine accuracy, specificity, sensitivity, and kappa value, as well as for visualization.

## Figures and Tables

**Table 1:** Patient characteristics of the two datasets with normal and tumor tissues including the Semmelweis cohort (*n* = 81 patients) used for MS and the gene chip cohort (*n* = 207 patients) collected from NCBI GEO.

Semmelweis Cohort			Gene Chip Cohort		
Min age	37		Min age	35	
Median age	62		Median age	64	
Max age	89		Max age	85	
Mean age	61.5 ± 10.8		Mean age	63.96 ± 13.12	
**Stage**	**N**	**%**	**Stage**	**N**	**%**
Stage I	30	37%	Stage I	46	22.2%
Stage II	8	9.9%	Stage II	27	13%
Stage III	38	46.9%	Stage III	29	14%
Stage IV	2	2.5%	Stage IV	18	8.7%
NA	3	3.7%	NA	87	57.9%
**Gender**	**N**	**%**	**Gender**	**N**	**%**
Male	50	61.7%	Male	40	19.2%
Female	31	38.3%	Female	22	10.6%
			NA	145	70.2
**Race**	**N**		**Smoker**	**N**	**%**
Caucasian	81		yes	23	11.1%
			no	40	19.3%
			NA	144	79.6%
			**Obese**	**N**	**%**
			yes	19	9.2%
			no	44	21.3%
			NA	144	69.5%

**Table 2:** Summary table of differential expression analysis of the twenty genes reaching significance in all cohorts. The nine genes used in the final SVM model building to detect ccRCC are highlighted with bold.

	Gene Chip Cohort		SE-MS Cohort	
	Fold-Change	Adjusted *p*	Fold-Change	Adjusted *p*
**ANXA1**	**2.89**	**1.02 ∗ 10^−31^**	**2.26**	**1.46 ∗ 10^−13^**
ARHGDIB	3.07	6.39 ∗ 10^−32^	1.68	4.83 ∗ 10^−7^
C1S	3.64	1.40 ∗ 10^−24^	1.22	0.1042807
**CCND1**	**4.12**	**4.09 ∗ 10^−31^**	**7.89**	**1.04 ∗ 10^−24^**
FN1	5.21	5.24 ∗ 10-^32^	1.99	2.31 ∗ 10^−8^
GPNMB	3.48	2.07 ∗ 10^−28^	2.11	1.02 ∗ 10^−7^
HLA-DPB1	3.45	3.13 ∗ 10^−31^	1.37	0.012
HLA-DRA	3.17	1.44 ∗ 10^−31^	1.31	0.056
HMOX1	2.95	4.14 ∗ 10^−28^	1.32	0.081
HPCAL1	2.86	4.26 ∗ 10^−31^	1.75	5.33 ∗ 10^−6^
**IGFBP3**	**8.15**	**5.88 ∗ 10^−32^**	**3.04**	**7.53 ∗ 10^−18^**
LGALS1	4.57	5.24 ∗ 10^−32^	1.76	6.03 ∗ 10^−8^
LIPA	3.07	5.24 ∗ 10^−32^	1.62	7.13 ∗ 10^−7^
MYOF	2.86	5.24 ∗ 10^−32^	1.87	5.39 ∗ 10^−8^
**P4HA1 **	**2.96**	**5.24 ∗ 10^−32^**	**3.15**	**2.30 ∗ 10^−22^**
**PFKP **	**5.69**	**5.24 ∗ 10^−32^**	**12.78**	**1.01 ∗ 10^−47^**
**PLIN2**	**3.85**	**3.09 ∗ 10^−31^**	**26.09**	**3.90 ∗ 10^−39^**
**PLOD2 **	**4.21**	**5.24 ∗ 10^−32^**	**15.84**	**6.51 ∗ 10^−36^**
RARRES2	3.35	2.11 ∗ 10^−30^	0.53	2.11 ∗ 10^−7^
TIMP1	3.61	5.24 ∗ 10^−32^	1.21	0.213
**VEGFA **	**3.02**	**5.11 ∗ 10^−31^**	**3.49**	**1.40 ∗ 10^−22^**
**VIM **	**2.88**	**7.36 ∗ 10^−32^**	**2.06**	**4.09 ∗ 10^−8^**

**Table 3:** Summary table of own MS data and CPTAC protein expression differences.

SE Data MS			CPTAC Protein Data	
	Fold-Change	Adjusted *p*-Value	Fold-Change	Adjusted *p*-Value
ANXA1	2.26	1.46 ∗ 10^−13^	2.31	6.60 ∗ 10^−41^
ARHGDIB	1.68	4.83 ∗ 10^−7^	1.87	7.10 ∗ 10^−42^
C1S	1.22	0.10	1.03	0.49
FN1	1.99	2.31 ∗ 10^−8^	1.91	1.90 ∗ 10^−25^
GPNMB	2.11	1.02 ∗ 10^−7^	2.23	2.60 ∗ 10^−17^
HLA-DPB1	1.37	0.01	1.96	3.10 ∗ 10^−32^
HLA-DRA	1.31	0.06	2.22	7.80 ∗ 10^−36^
HMOX1	1.32	0.08	1.67	1.20 ∗ 10^−29^
HPCAL1	1.75	5.33 ∗ 10^−6^	2.50	5.00 ∗ 10^−45^
IGFBP3	3.04	7.53 ∗ 10^−18^	2.28	2.10 ∗ 10^−31^
LGALS1	1.76	6.03 ∗ 10^−8^	1.77	1.60 ∗ 10^−33^
LIPA	1.62	7.13 ∗ 10^−7^	1.91	9.40 ∗ 10^−31^
MYOF	1.87	5.39 ∗ 10^−8^	1.88	2.00 ∗ 10^−39^
P4HA1	3.15	2.30 ∗ 10^−22^	3.20	9.90 ∗ 10^−57^
PFKP	12.78	1.01 ∗ 10^−47^	4.20	4.30 ∗ 10^−56^
PLIN2	26.09	3.90 ∗ 10^−39^	6.92	1.70 ∗ 10^−33^
PLOD2	15.84	6.51 ∗ 10^−36^	4.89	7.40 ∗ 10^−33^
RARRES2	0.53	2.11 ∗ 10^−7^	0.76	1.20 ∗ 10^−13^
TIMP1	1.21	0.21	1.10	0.17
VEGFA	3.49	1.40 ∗ 10^−22^	3.12	3.00 ∗ 10^−32^
VIM	2.06	4.09 ∗ 10^−8^	2.27	1.70 ∗ 10^−63^
CCND1	7.89	1.04 ∗ 10^−24^	-	-

**Table 4:** Summary table of classification accuracy, sensitivity, specificity, and Kappa values in the test set by each applied method. KNN: k-nearest neighbors, RF: random forest, LOGIT: logistic regression, and SVM: support vector machines.

	RF	SVM	KNN	LOGIT
**Accuracy**	0.958	0.979	0.9375	0.958
**Kappa**	0.916	0.958	0.8750	0.916
**Sensitivity**	0.916	0.958	0.8750	0.916
**Specificity**	1.0	1.0	1.0	1.0

**Table 5:** Summary table of ideal gene panels in each algorithm. KNN: k-nearest neighbors, RF: random forest, LOGIT: logistic regression, and SVM: support vector machines.

**RF**	PFKP	PLOD2	PLIN2						
**SVM**	PFKP	PLIN2	PLOD2	IGFBP3	VEGFA	P4HA1	CCND1	VIM	ANXA1
**KNN**	PFKP	PLIN2	PLOD2	IGFBP3	VEGFA	P4HA1	CCND1		
**LOGIT**	PFKP	PLIN2	PLOD2						

## Data Availability

The datasets generated during and/or analyzed during the current study are available from this link: https://github.com/4ronB/Multi-omic-discrimination-of-tumor-and-normal-tissues-in-renal-cell-carcinoma-, accessed on 3 January 2023; The raw mass spectrometry proteomics data have been deposited to the ProteomeXchange Consortium via the PRIDE partner repository with the dataset identifier PXD033709.

## Supplementary Materials

The following supporting information can be downloaded at: https://www.mdpi.com/article/10.3390/ijms24054488/s1.
