# Supplementary material for: Proteotranscriptomic Discrimination of Tumor and Normal Tissues in Renal Cell Carcinoma
Source: Int J Mol Sci. 2023 Feb 24;24(5):4488. doi: 10.3390/ijms24054488 (PMC10003397; doi:10.3390/ijms24054488)
Supplement: Supplementary file 1 [file ijms-24-04488-s001.zip › Results_Supplemental Figure S1.docx]

**Figure S1.** Dot plot of significant GO biological process categories, the size of the circles represents the number of genes connected to a specific function and adjusted p values are represented by the color of the circles **(A)**. The barplot represents the most significantly enriched terms, the color of each bars represents the adjusted p values according to the enrichment analysis **(B)**.

| **A** | **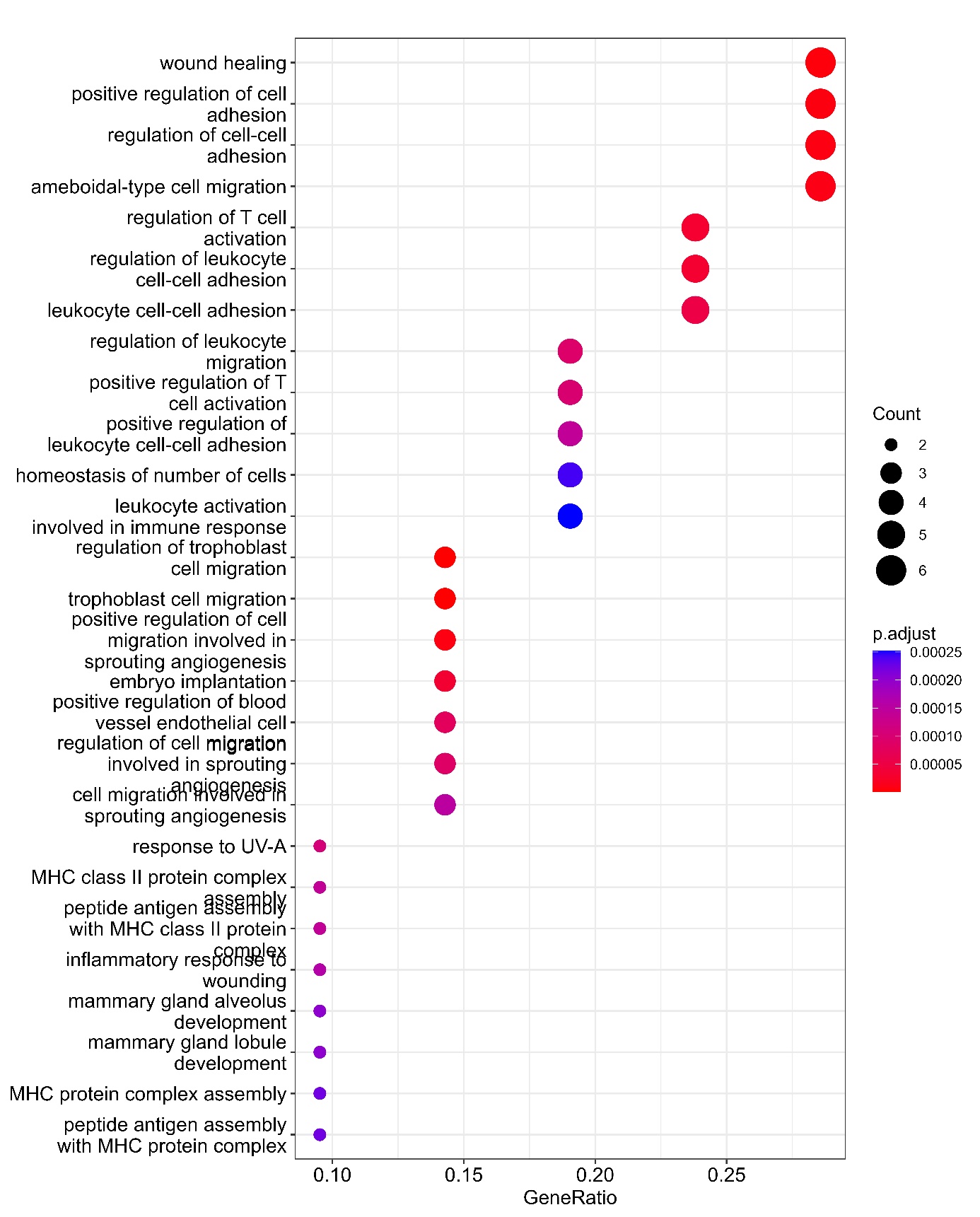** |
| --- | --- |
| **B** | **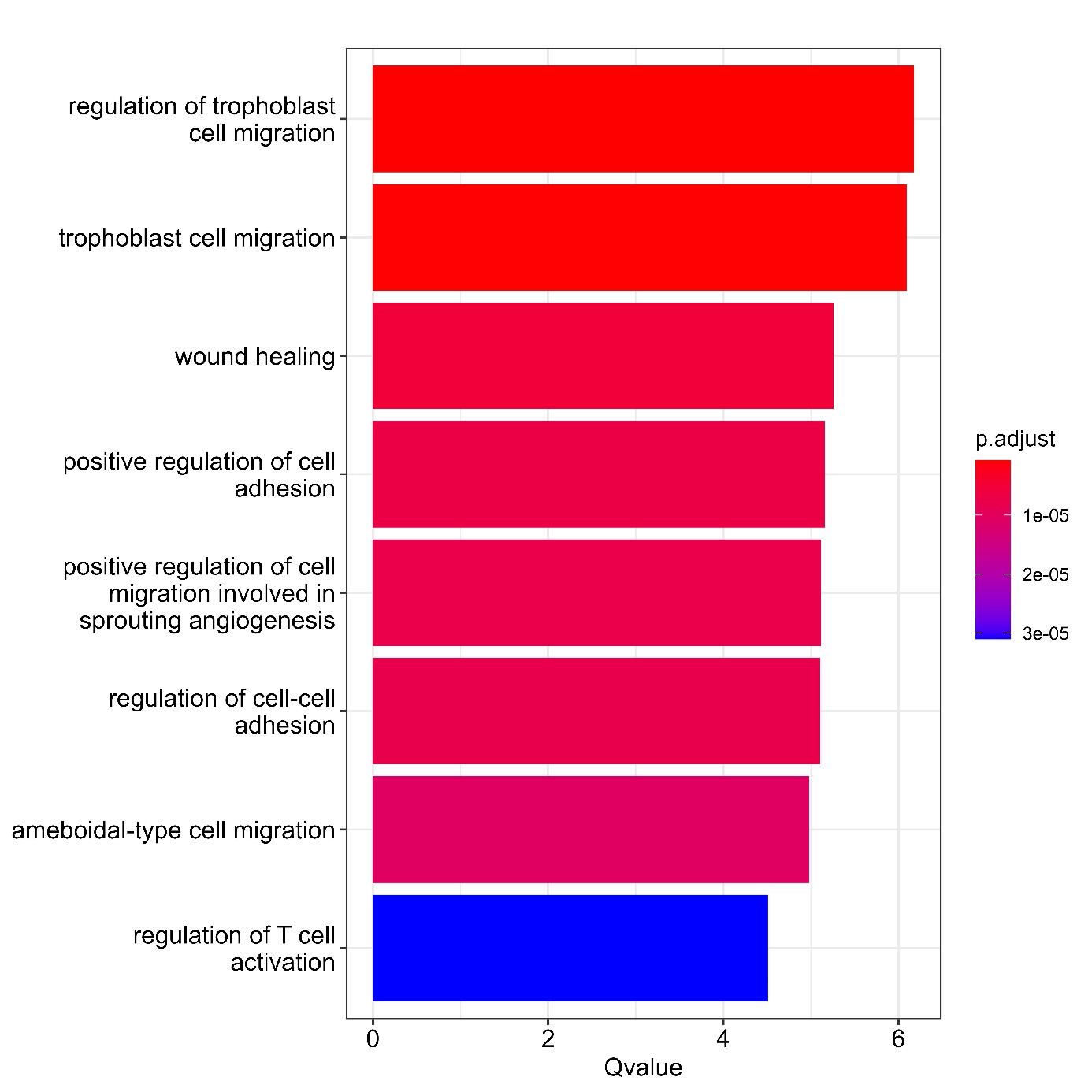** |
