# Supplementary material for: Proteotranscriptomic Discrimination of Tumor and Normal Tissues in Renal Cell Carcinoma
Source: Int J Mol Sci. 2023 Feb 24;24(5):4488. doi: 10.3390/ijms24054488 (PMC10003397; doi:10.3390/ijms24054488)
Supplement: Supplementary file 1 [file ijms-24-04488-s001.zip › Results_Supplemental Figure S2.pdf]

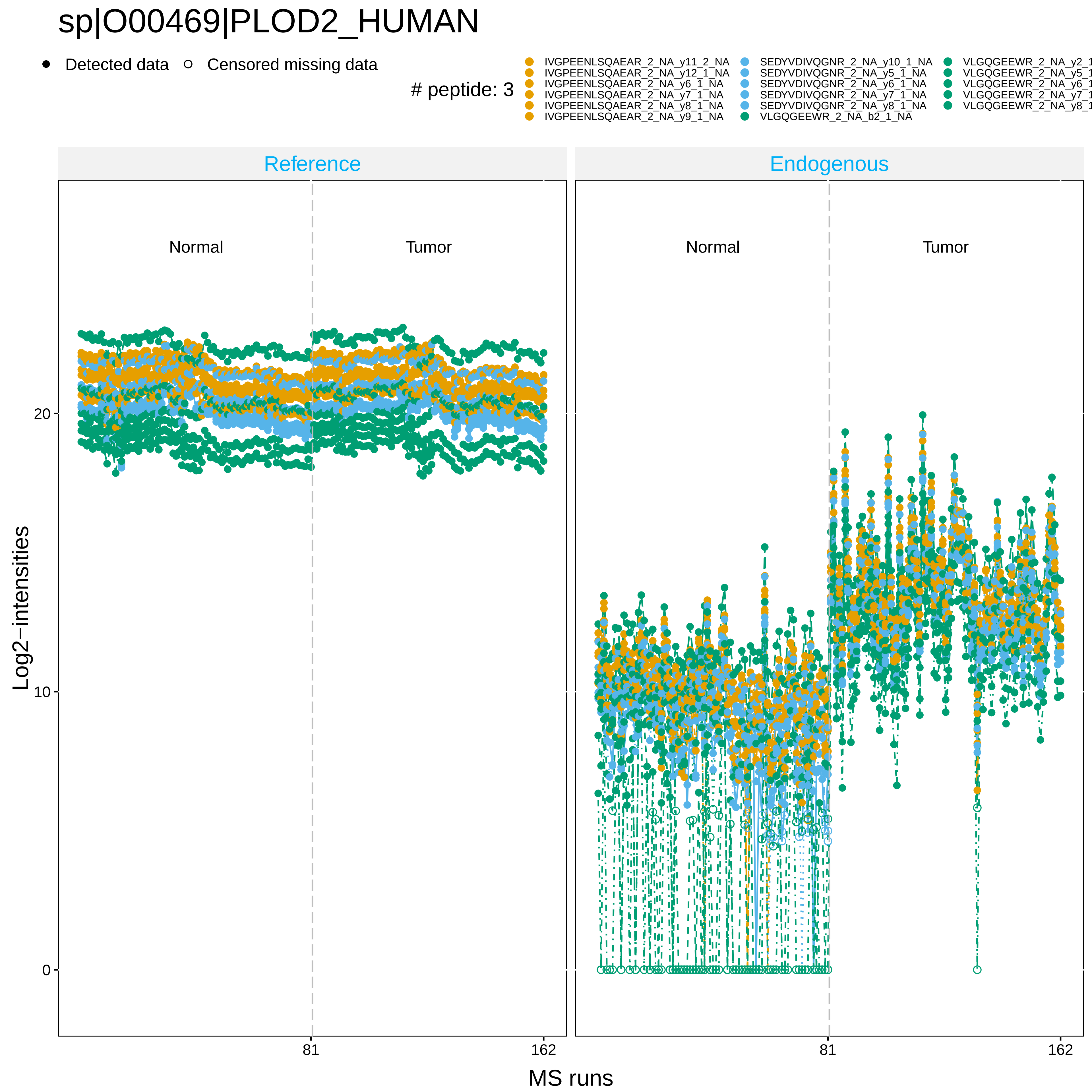

sp|O00469|PLOD2_HUMAN
Detected data
Censored missing data
peptide: 3
IVGPEENLSQAEAR_2_NA_y11_2_NA
IVGPEENLSQAEAR_2_NA_y12_1_NA
IVGPEENLSQAEAR_2_NA_y6_1_NA
IVGPEENLSQAEAR_2_NA_y7_1_NA
IVGPEENLSQAEAR_2_NA_y8_1_NA
IVGPEENLSQAEAR_2_NA_y9_1_NA
SEDYVDIVQGNR_2_NA_y10_1_NA
SEDYVDIVQGNR_2_NA_y5_1_NA
SEDYVDIVQGNR_2_NA_y6_1_NA
SEDYVDIVQGNR_2_NA_y7_1_NA
SEDYVDIVQGNR_2_NA_y8_1_NA
VLGQGEEWR_2_NA_b2_1_NA
Reference
Endogenous
Normal
Tumor
Log2−intensities
20
10
0
81
162
MS runs

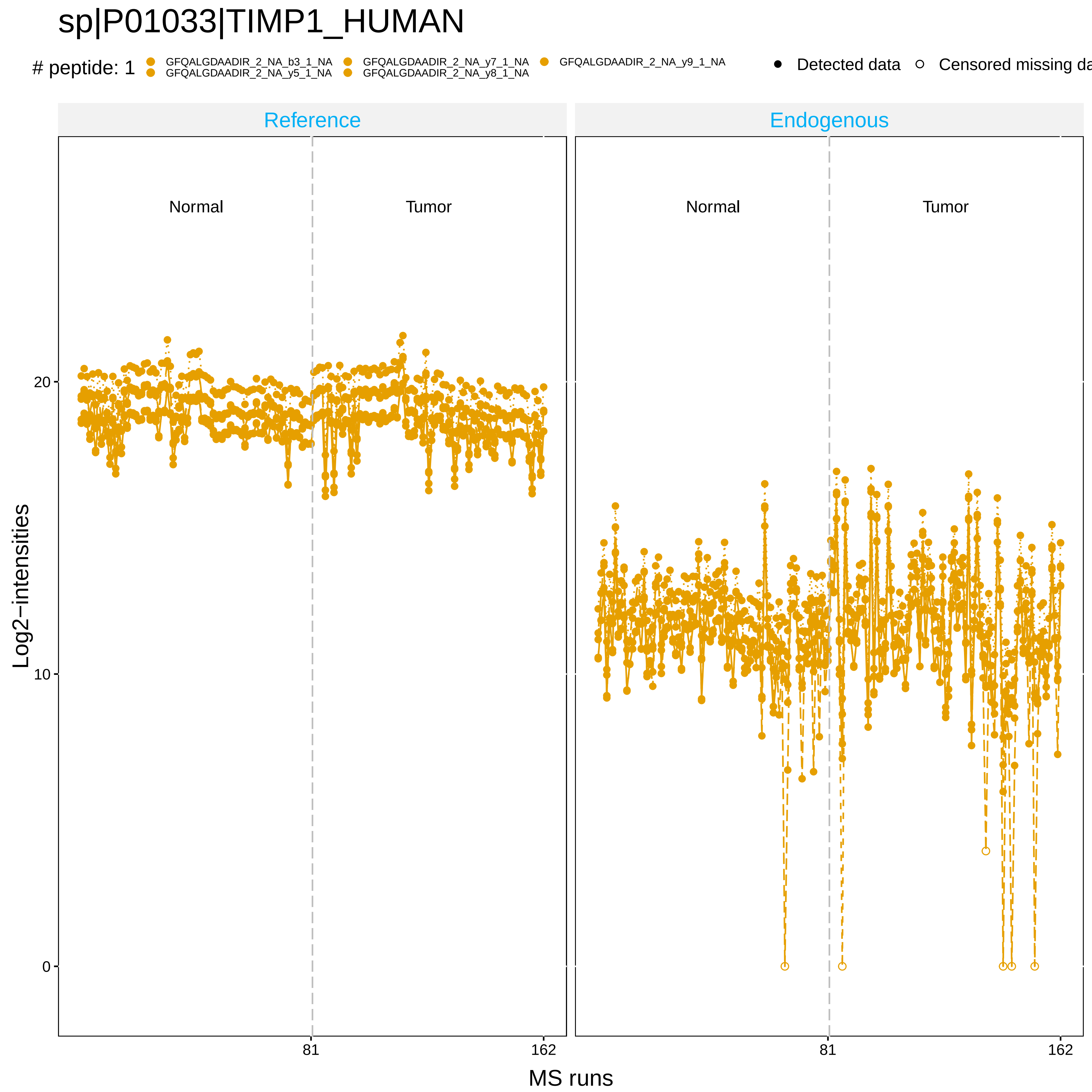

sp|P01033|TIMP1_HUMAN
peptide: 1
GFQALGDAADIR_2_NA_b3_1_NA
GFQALGDAADIR_2_NA_y5_1_NA
GFQALGDAADIR_2_NA_y7_1_NA
GFQALGDAADIR_2_NA_y8_1_NA
GFQALGDAADIR_2_NA_y9_1_NA
Detected data
Censored missing da
Reference
Endogenous
Normal
Tumor
Normal
Tumor
Log2−intensities
20
10
0
81
162
81
162
MS runs

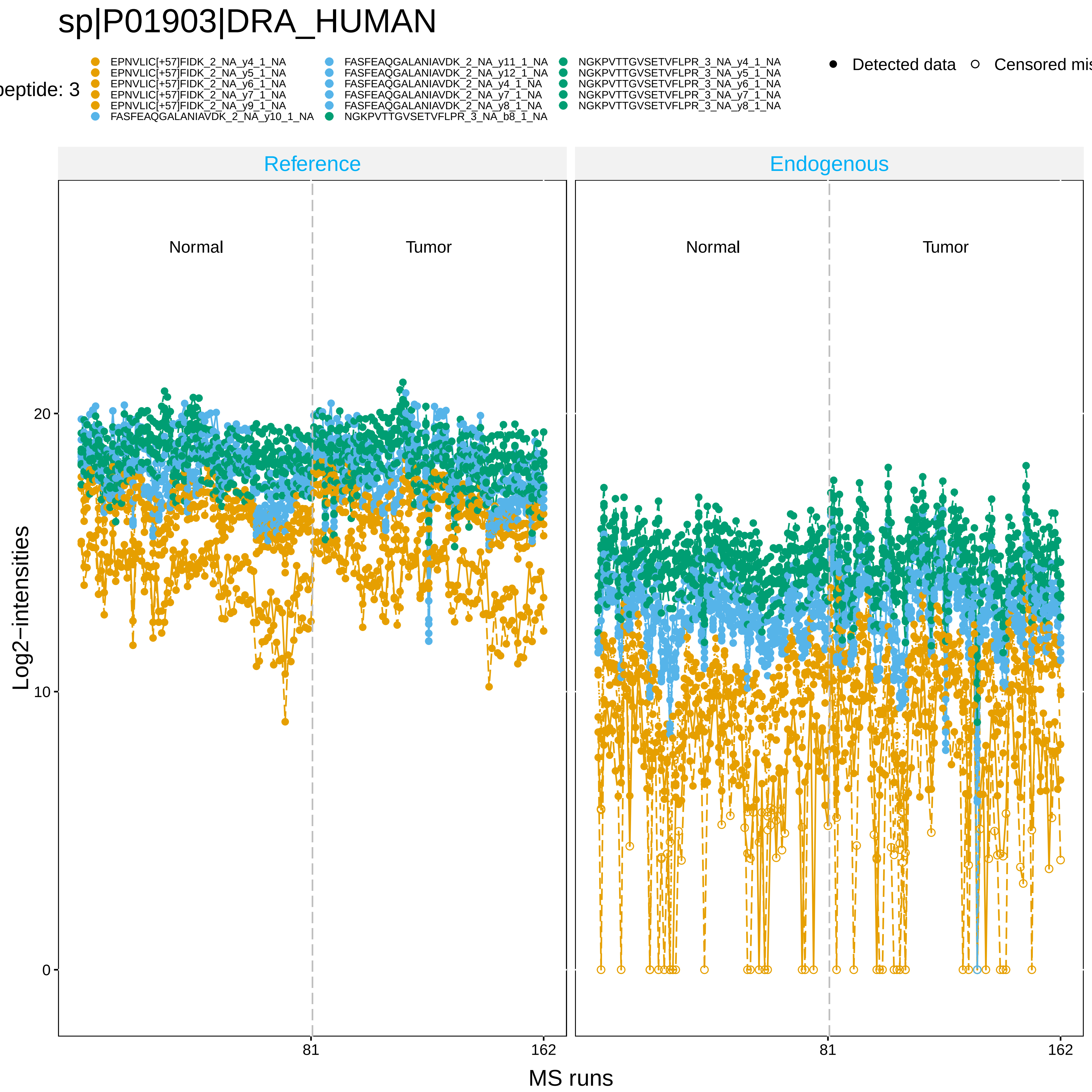
sp|P01903|DRA_HUMAN
peptide: 3
EPNVLIC[+57]FIDK_2_NA_y4_1_NA
EPNVLIC[+57]FIDK_2_NA_y5_1_NA
EPNVLIC[+57]FIDK_2_NA_y6_1_NA
EPNVLIC[+57]FIDK_2_NA_y7_1_NA
EPNVLIC[+57]FIDK_2_NA_y9_1_NA
FASFEAQGALANIAVDK_2_NA_y10_1_NA
FASFEAQGALANIAVDK_2_NA_y11_1_NA
FASFEAQGALANIAVDK_2_NA_y12_1_NA
FASFEAQGALANIAVDK_2_NA_y4_1_NA
FASFEAQGALANIAVDK_2_NA_y7_1_NA
FASFEAQGALANIAVDK_2_NA_y8_1_NA
NGKPVTTGVSETVFLPR_3_NA_b8_1_NA
NGKPVTTGVSETVFLPR_3_NA_y4_1_NA
NGKPVTTGVSETVFLPR_3_NA_y5_1_NA
NGKPVTTGVSETVFLPR_3_NA_y6_1_NA
NGKPVTTGVSETVFLPR_3_NA_y7_1_NA
NGKPVTTGVSETVFLPR_3_NA_y8_1_NA
Detected data
Censored mis
Reference
Endogenous
Normal
Tumor
Normal
Tumor
Log2−intensities
20
10
0
81
162
81
162
MS runs

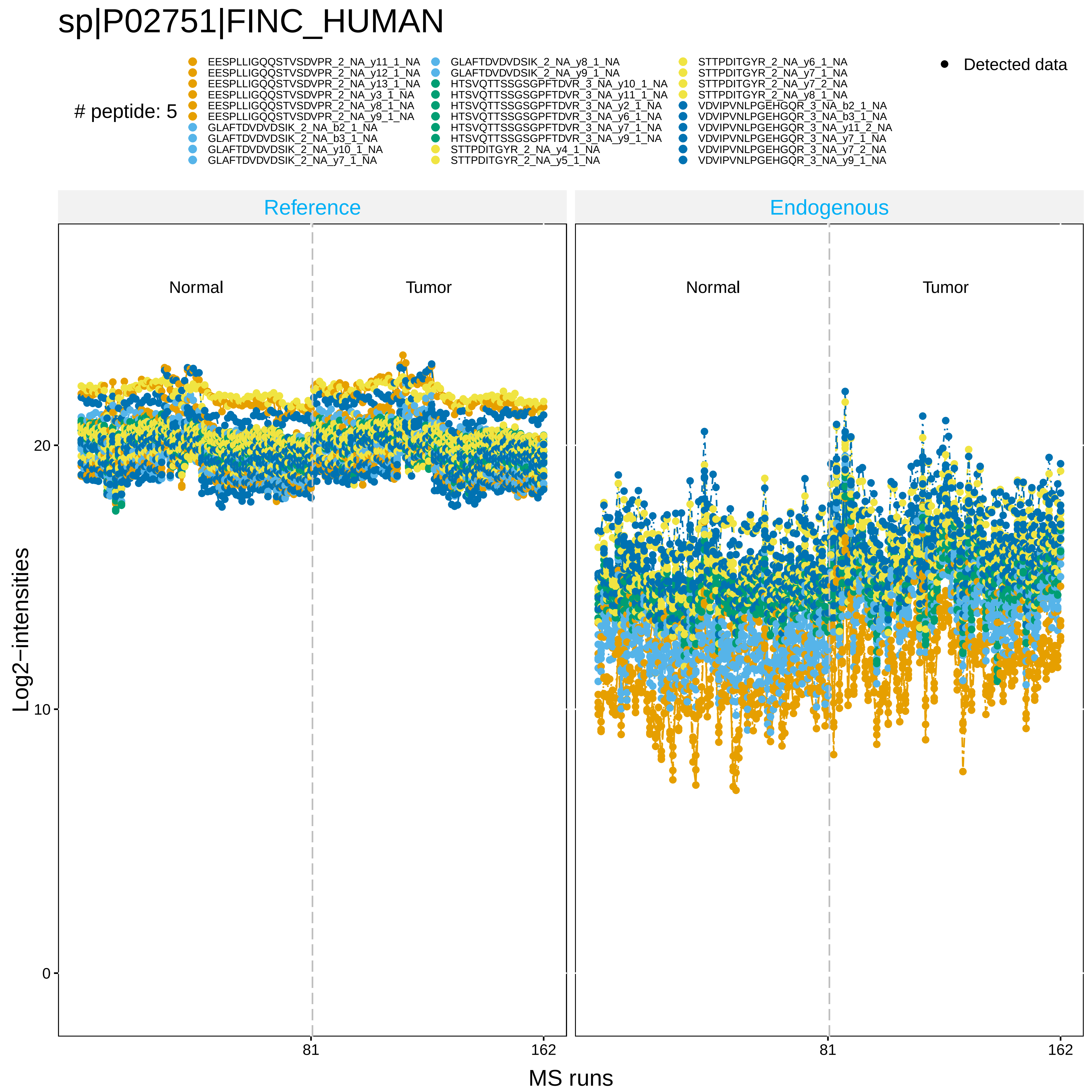

sp|P02751|FINC_HUMAN
peptide: 5
EESPLLIGQQSTVSDVPR_2_NA_y11_1_NA
EESPLLIGQQSTVSDVPR_2_NA_y12_1_NA
EESPLLIGQQSTVSDVPR_2_NA_y13_1_NA
EESPLLIGQQSTVSDVPR_2_NA_y3_1_NA
EESPLLIGQQSTVSDVPR_2_NA_y8_1_NA
EESPLLIGQQSTVSDVPR_2_NA_y9_1_NA
GLAFTDVDVDSIK_2_NA_b2_1_NA
GLAFTDVDVDSIK_2_NA_b3_1_NA
GLAFTDVDVDSIK_2_NA_y10_1_NA
GLAFTDVDVDSIK_2_NA_y7_1_NA
GLAFTDVDVDSIK_2_NA_y8_1_NA
GLAFTDVDVDSIK_2_NA_y9_1_NA
HTSVQTTSSGSGPFTDVR_3_NA_y10_1_NA
HTSVQTTSSGSGPFTDVR_3_NA_y11_1_NA
HTSVQTTSSGSGPFTDVR_3_NA_y2_1_NA
HTSVQTTSSGSGPFTDVR_3_NA_y6_1_NA
HTSVQTTSSGSGPFTDVR_3_NA_y7_1_NA
HTSVQTTSSGSGPFTDVR_3_NA_y9_1_NA
STTPDITGYR_2_NA_y4_1_NA
STTPDITGYR_2_NA_y5_1_NA
STTPDITGYR_2_NA_y6_1_NA
STTPDITGYR_2_NA_y7_1_NA
STTPDITGYR_2_NA_y7_2_NA
STTPDITGYR_2_NA_y8_1_NA
VDVIPVNLPGEHGQR_3_NA_b2_1_NA
VDVIPVNLPGEHGQR_3_NA_b3_1_NA
VDVIPVNLPGEHGQR_3_NA_y11_2_NA
VDVIPVNLPGEHGQR_3_NA_y7_1_NA
VDVIPVNLPGEHGQR_3_NA_y7_2_NA
VDVIPVNLPGEHGQR_3_NA_y9_1_NA
Detected data
Reference
Endogenous
Normal
Tumor
Normal
Tumor
Log2−intensities
20
10
0
81
162
81
162
MS runs

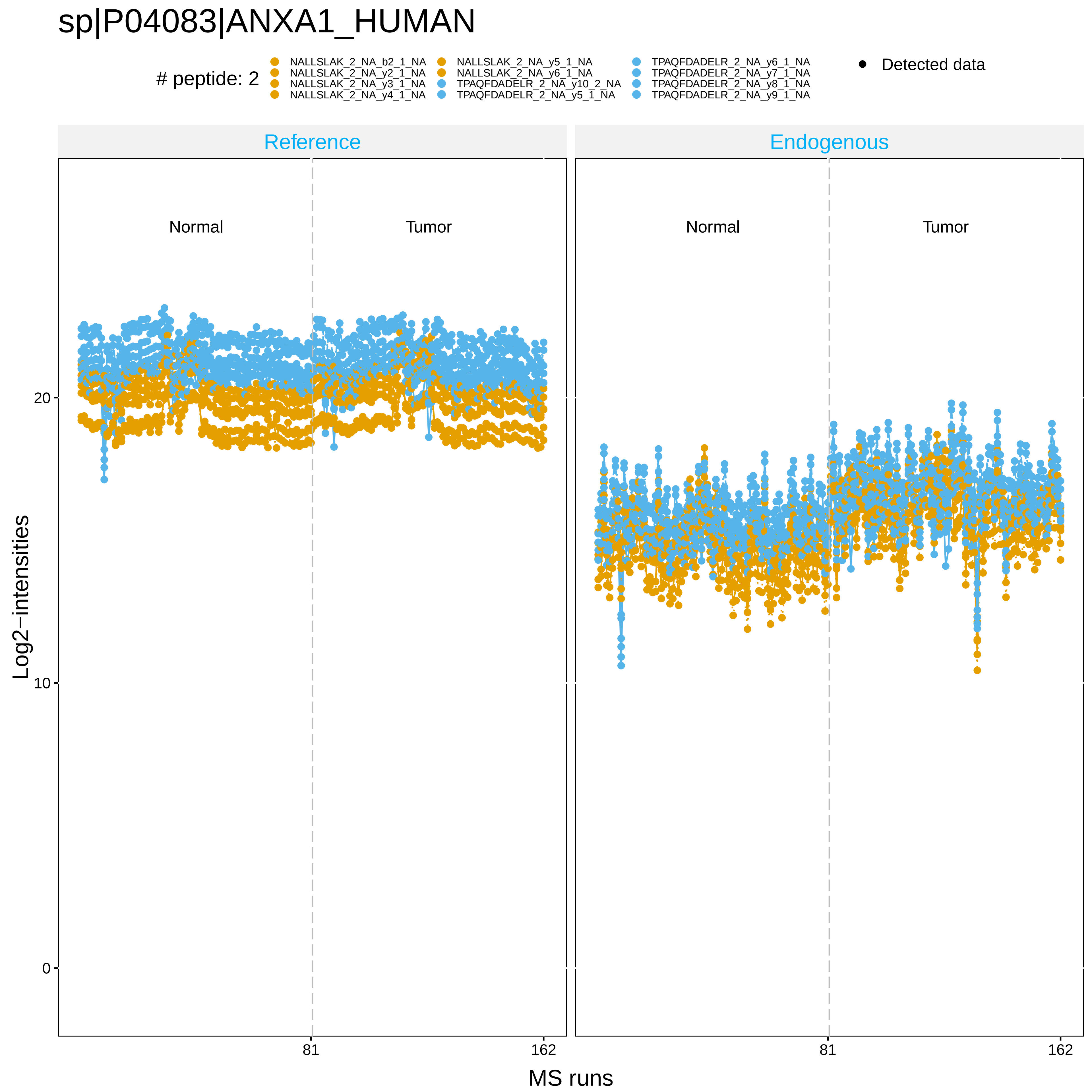
sp|P04083|ANXA1_HUMAN
peptide: 2
NALLSLAK_2_NA_b2_1_NA
NALLSLAK_2_NA_y2_1_NA
NALLSLAK_2_NA_y3_1_NA
NALLSLAK_2_NA_y4_1_NA
NALLSLAK_2_NA_y5_1_NA
NALLSLAK_2_NA_y6_1_NA
TPAQFDADELR_2_NA_y10_2_NA
TPAQFDADELR_2_NA_y5_1_NA
TPAQFDADELR_2_NA_y6_1_NA
TPAQFDADELR_2_NA_y7_1_NA
TPAQFDADELR_2_NA_y8_1_NA
TPAQFDADELR_2_NA_y9_1_NA
Detected data
Reference
Endogenous
Normal
Tumor
Normal
Tumor
Log2−intensities
20
10
0
81
162
81
162
MS runs

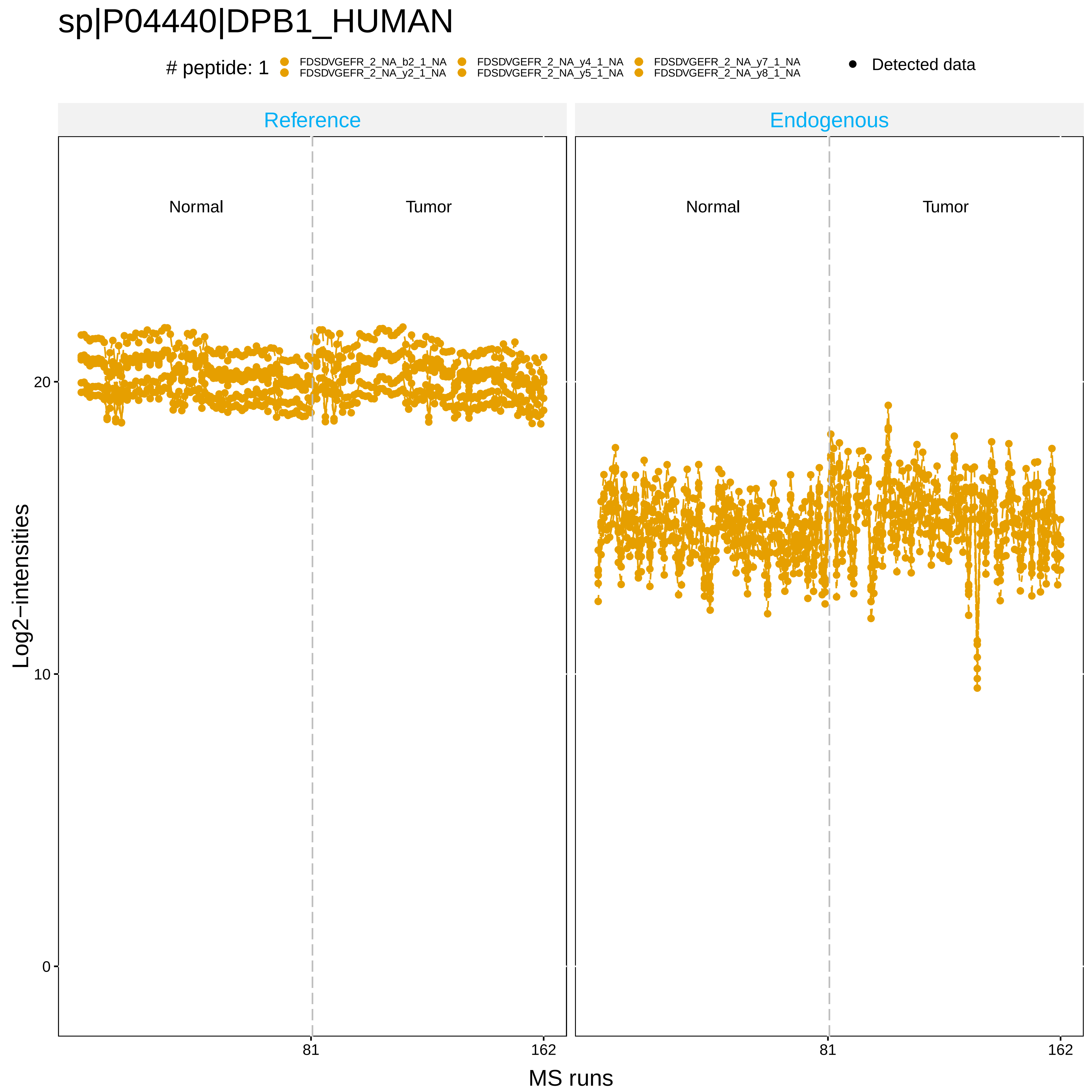

sp|P04440|DPB1_HUMAN
peptide: 1
FDSDVGEFR_2_NA_b2_1_NA
FDSDVGEFR_2_NA_y2_1_NA
FDSDVGEFR_2_NA_y4_1_NA
FDSDVGEFR_2_NA_y5_1_NA
FDSDVGEFR_2_NA_y7_1_NA
FDSDVGEFR_2_NA_y8_1_NA
Detected data
Reference
Endogenous
Normal
Tumor
Normal
Tumor
Log2−intensities
20
10
0
81
162
81
162
MS runs

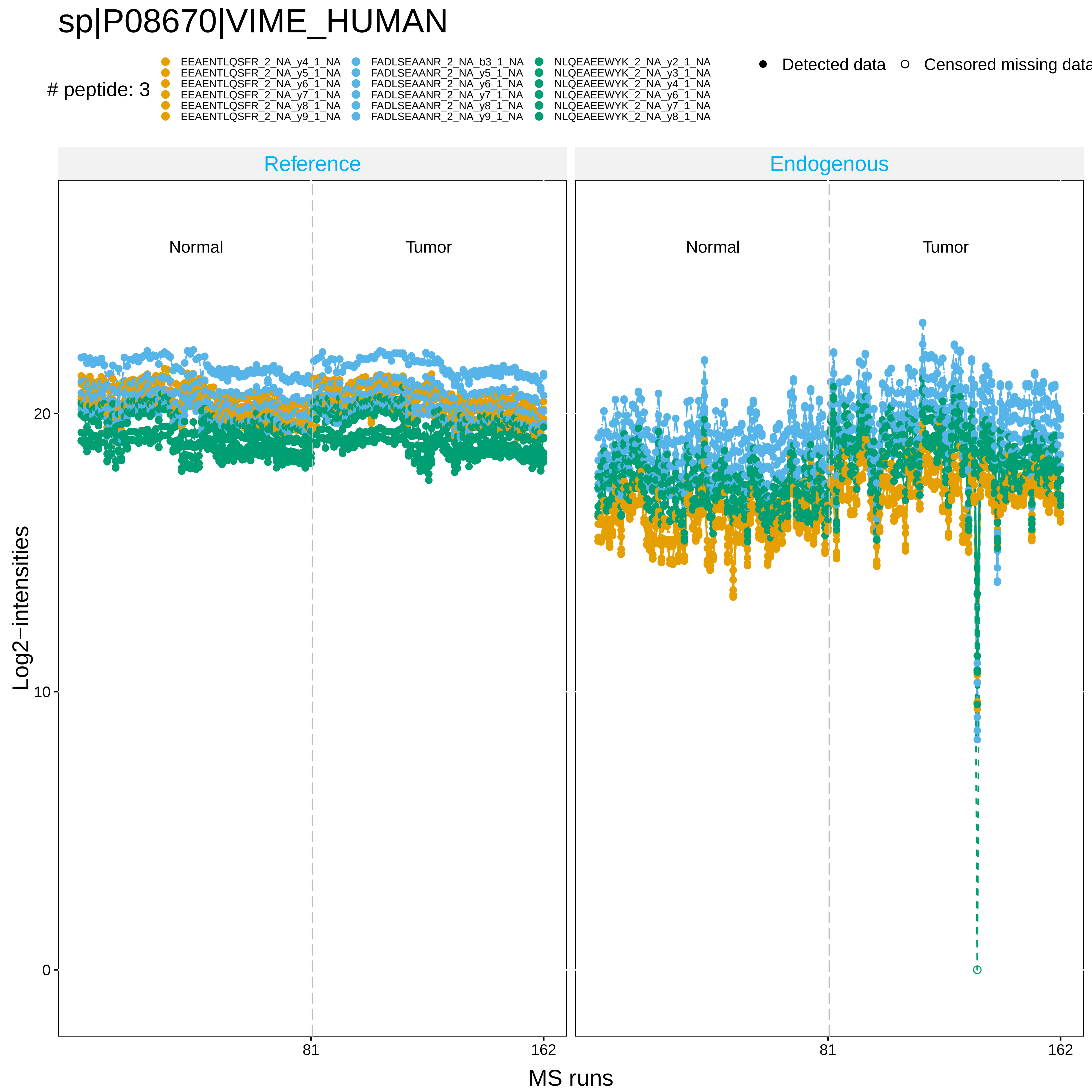

sp|P08670|VIME_HUMAN
peptide: 3
EEAENTLQSFR_2_NA_y4_1_NA
EEAENTLQSFR_2_NA_y5_1_NA
EEAENTLQSFR_2_NA_y6_1_NA
EEAENTLQSFR_2_NA_y7_1_NA
EEAENTLQSFR_2_NA_y8_1_NA
EEAENTLQSFR_2_NA_y9_1_NA
FADLSEAANR_2_NA_b3_1_NA
FADLSEAANR_2_NA_y5_1_NA
FADLSEAANR_2_NA_y6_1_NA
FADLSEAANR_2_NA_y7_1_NA
FADLSEAANR_2_NA_y8_1_NA
FADLSEAANR_2_NA_y9_1_NA
NLQEAEEWYK_2_NA_y2_1_NA
NLQEAEEWYK_2_NA_y3_1_NA
NLQEAEEWYK_2_NA_y4_1_NA
NLQEAEEWYK_2_NA_y6_1_NA
NLQEAEEWYK_2_NA_y7_1_NA
NLQEAEEWYK_2_NA_y8_1_NA
Detected data
Censored missing data
Reference
Endogenous
Normal
Tumor
Normal
Tumor
Log2−intensities
20
10
0
81
162
81
162
MS runs

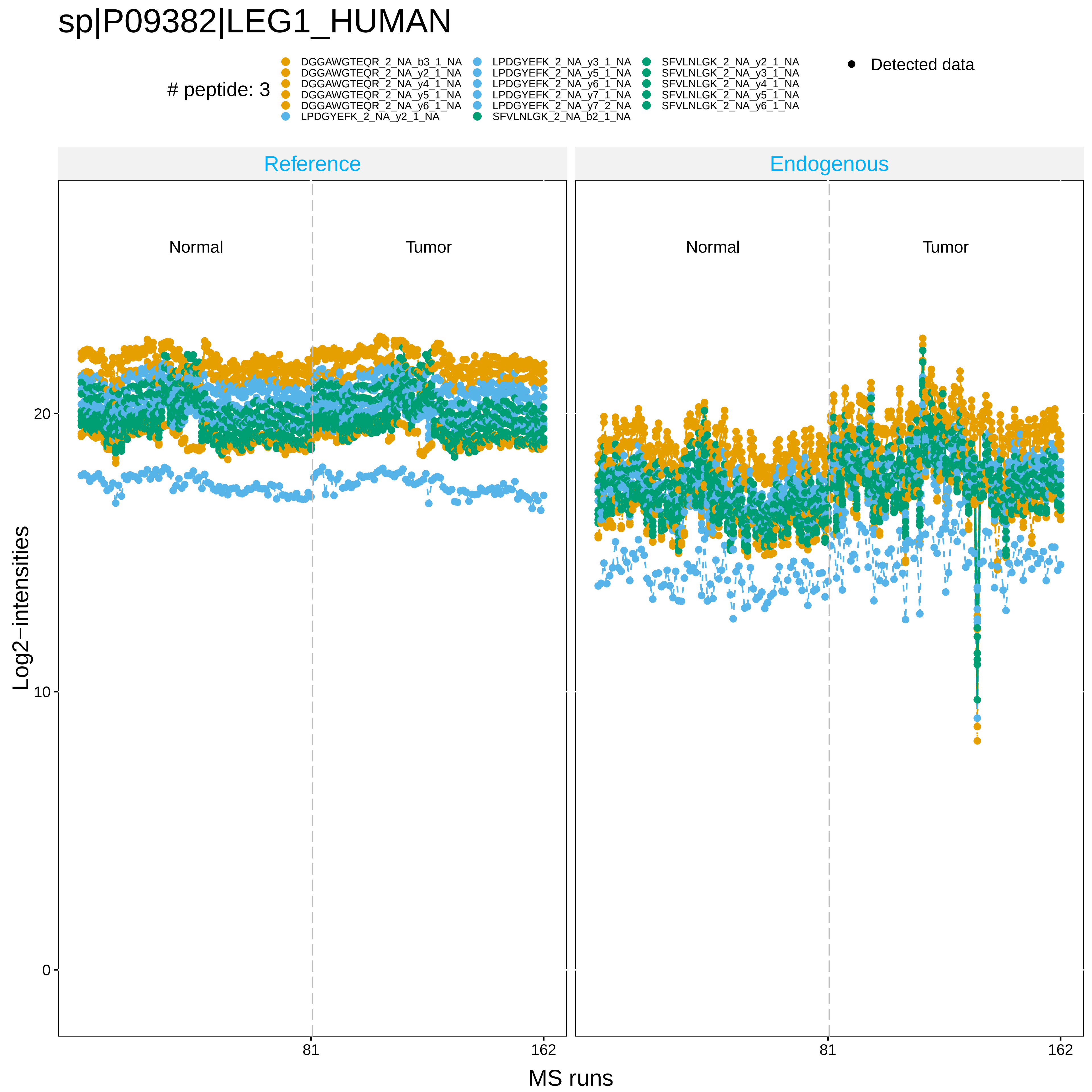

sp|P09382|LEG1_HUMAN
peptide: 3
DGGAWGTEQR_2_NA_b3_1_NA
DGGAWGTEQR_2_NA_y2_1_NA
DGGAWGTEQR_2_NA_y4_1_NA
DGGAWGTEQR_2_NA_y5_1_NA
DGGAWGTEQR_2_NA_y6_1_NA
LPDGYEFK_2_NA_y2_1_NA
LPDGYEFK_2_NA_y3_1_NA
LPDGYEFK_2_NA_y5_1_NA
LPDGYEFK_2_NA_y6_1_NA
LPDGYEFK_2_NA_y7_1_NA
LPDGYEFK_2_NA_y7_2_NA
SFVLNLGK_2_NA_b2_1_NA
SFVLNLGK_2_NA_y2_1_NA
SFVLNLGK_2_NA_y3_1_NA
SFVLNLGK_2_NA_y4_1_NA
SFVLNLGK_2_NA_y5_1_NA
SFVLNLGK_2_NA_y6_1_NA
Detected data
Reference
Endogenous
Normal
Tumor
Normal
Tumor
Log2−intensities
20
10
0
81
162
81
162
MS runs

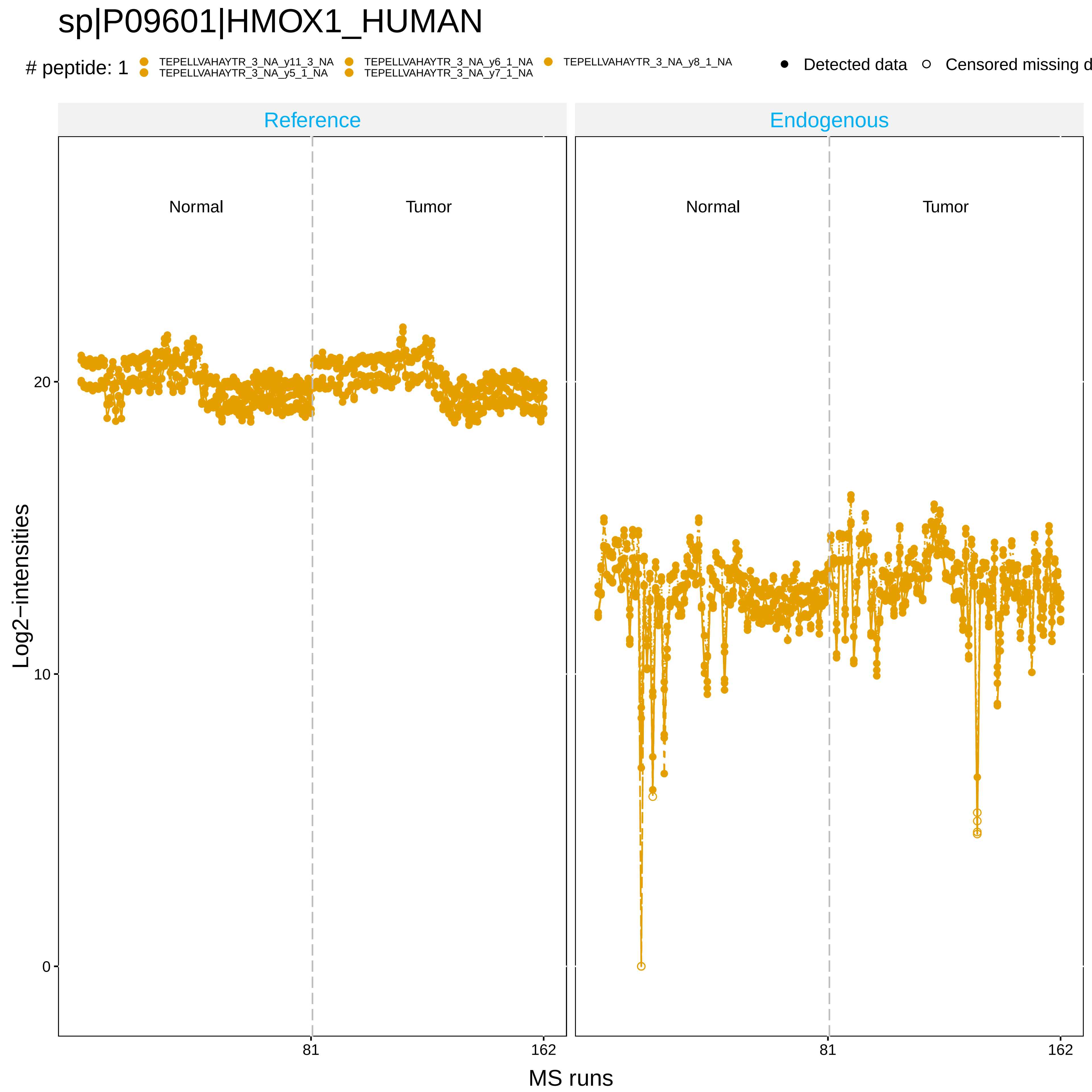
sp|P09601|HMOX1_HUMAN
peptide: 1
TEPELLVAHAYTR_3_NA_y11_3_NA
TEPELLVAHAYTR_3_NA_y5_1_NA
TEPELLVAHAYTR_3_NA_y6_1_NA
TEPELLVAHAYTR_3_NA_y7_1_NA
TEPELLVAHAYTR_3_NA_y8_1_NA
Detected data
Censored missing d
Reference
Endogenous
Normal
Tumor
Log2−intensities
MS runs
0
10
20
81
162

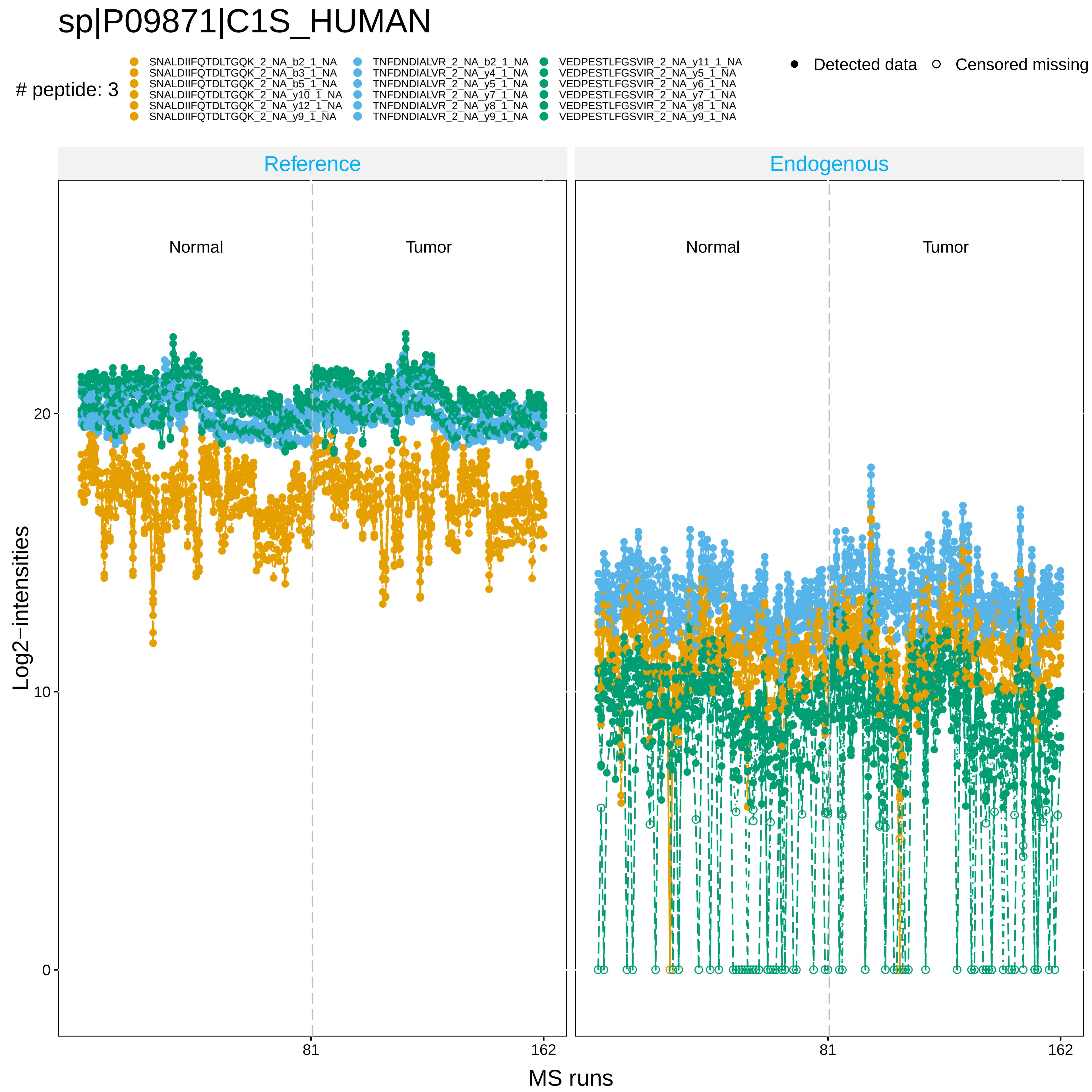
sp|P09871|C1S_HUMAN
peptide: 3
SNALDIIFQTDLTGQK_2_NA_b2_1_NA
SNALDIIFQTDLTGQK_2_NA_b3_1_NA
SNALDIIFQTDLTGQK_2_NA_b5_1_NA
SNALDIIFQTDLTGQK_2_NA_y10_1_NA
SNALDIIFQTDLTGQK_2_NA_y12_1_NA
SNALDIIFQTDLTGQK_2_NA_y9_1_NA
TNFDNDIALVR_2_NA_b2_1_NA
TNFDNDIALVR_2_NA_y4_1_NA
TNFDNDIALVR_2_NA_y5_1_NA
TNFDNDIALVR_2_NA_y7_1_NA
TNFDNDIALVR_2_NA_y8_1_NA
TNFDNDIALVR_2_NA_y9_1_NA
VEDPESTLFGSVIR_2_NA_y11_1_NA
VEDPESTLFGSVIR_2_NA_y5_1_NA
VEDPESTLFGSVIR_2_NA_y6_1_NA
VEDPESTLFGSVIR_2_NA_y7_1_NA
VEDPESTLFGSVIR_2_NA_y8_1_NA
VEDPESTLFGSVIR_2_NA_y9_1_NA
Detected data
Censored missing
Reference
Endogenous
Normal
Tumor
Normal
Tumor
Log2−intensities
20
10
0
81
162
81
162
MS runs

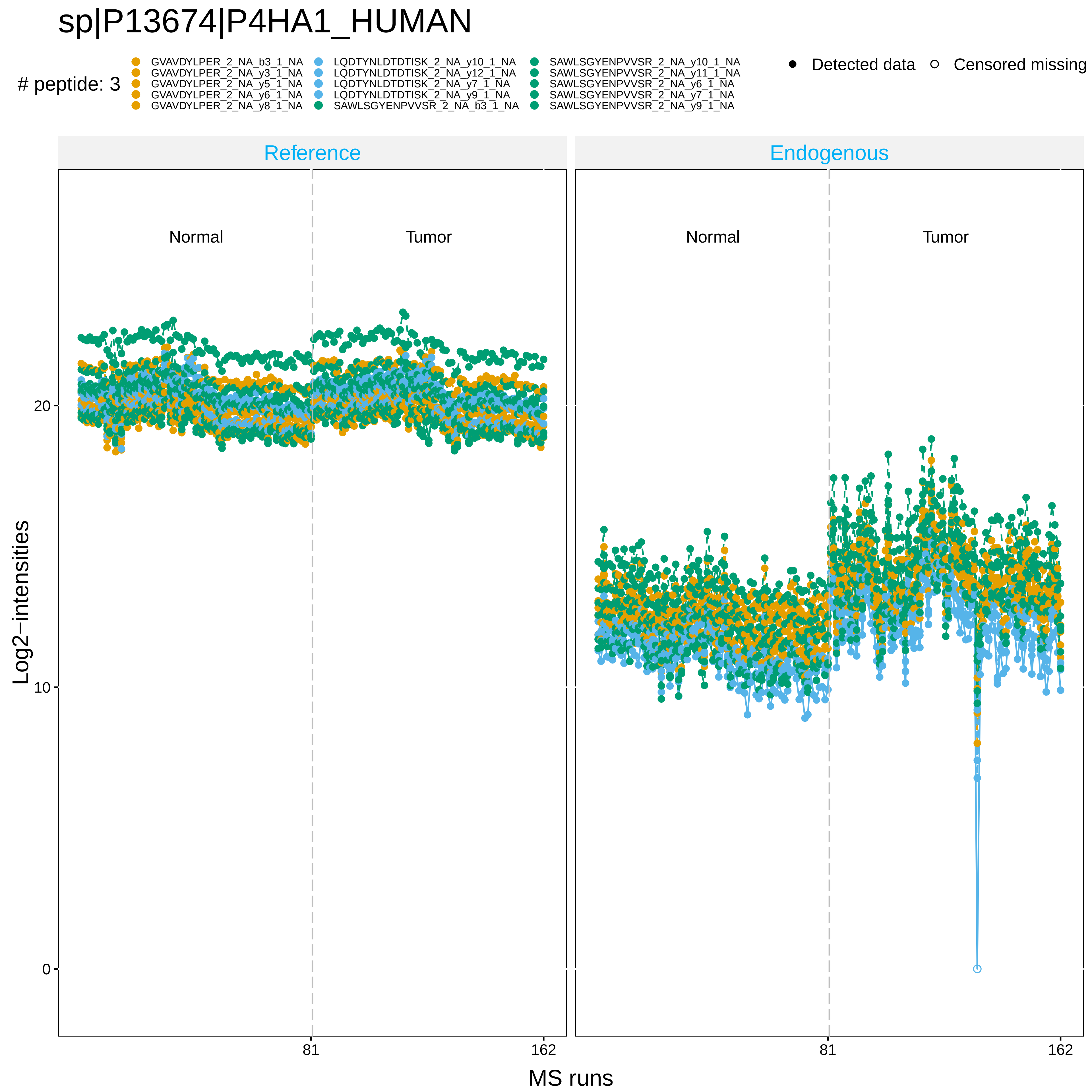

sp|P13674|P4HA1_HUMAN
peptide: 3
GVAVDYLPER_2_NA_b3_1_NA
GVAVDYLPER_2_NA_y3_1_NA
GVAVDYLPER_2_NA_y5_1_NA
GVAVDYLPER_2_NA_y6_1_NA
GVAVDYLPER_2_NA_y8_1_NA
LQDTYNLDTDTISK_2_NA_y10_1_NA
LQDTYNLDTDTISK_2_NA_y12_1_NA
LQDTYNLDTDTISK_2_NA_y7_1_NA
LQDTYNLDTDTISK_2_NA_y9_1_NA
SAWLSGYENPVVSR_2_NA_b3_1_NA
SAWLSGYENPVVSR_2_NA_y10_1_NA
SAWLSGYENPVVSR_2_NA_y11_1_NA
SAWLSGYENPVVSR_2_NA_y6_1_NA
SAWLSGYENPVVSR_2_NA_y7_1_NA
SAWLSGYENPVVSR_2_NA_y9_1_NA
Detected data
Censored missing
Reference
Endogenous
Normal
Tumor
Normal
Tumor
Log2−intensities
20
10
0
81
162
81
162
MS runs

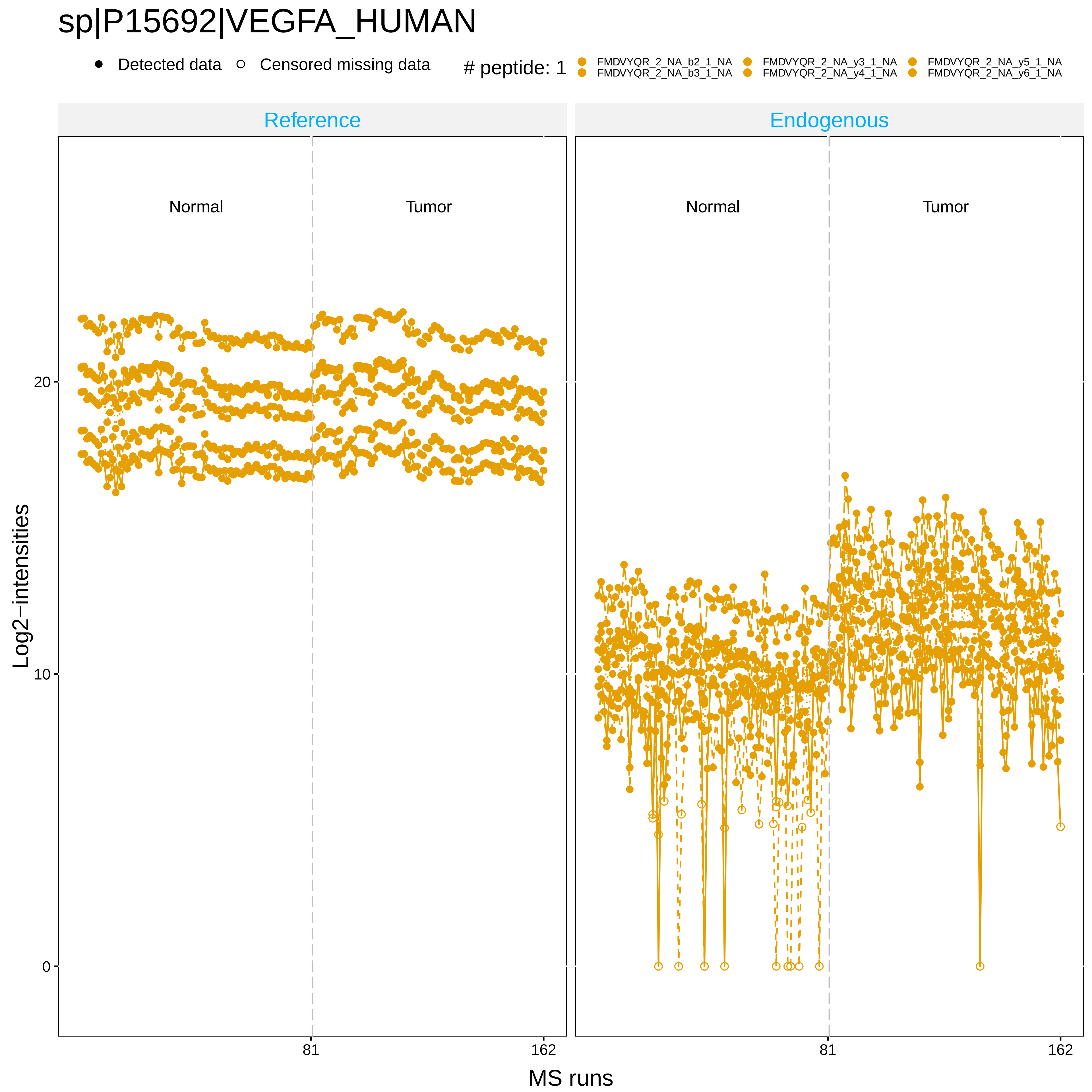

sp|P15692|VEGFA_HUMAN
Detected data
Censored missing data
peptide: 1
FMDVYQR_2_NA_b2_1_NA
FMDVYQR_2_NA_b3_1_NA
FMDVYQR_2_NA_y3_1_NA
FMDVYQR_2_NA_y4_1_NA
FMDVYQR_2_NA_y5_1_NA
FMDVYQR_2_NA_y6_1_NA
Reference
Endogenous
Normal
Tumor
Normal
Tumor
Log2−intensities
20
10
0
81
162
81
162
MS runs

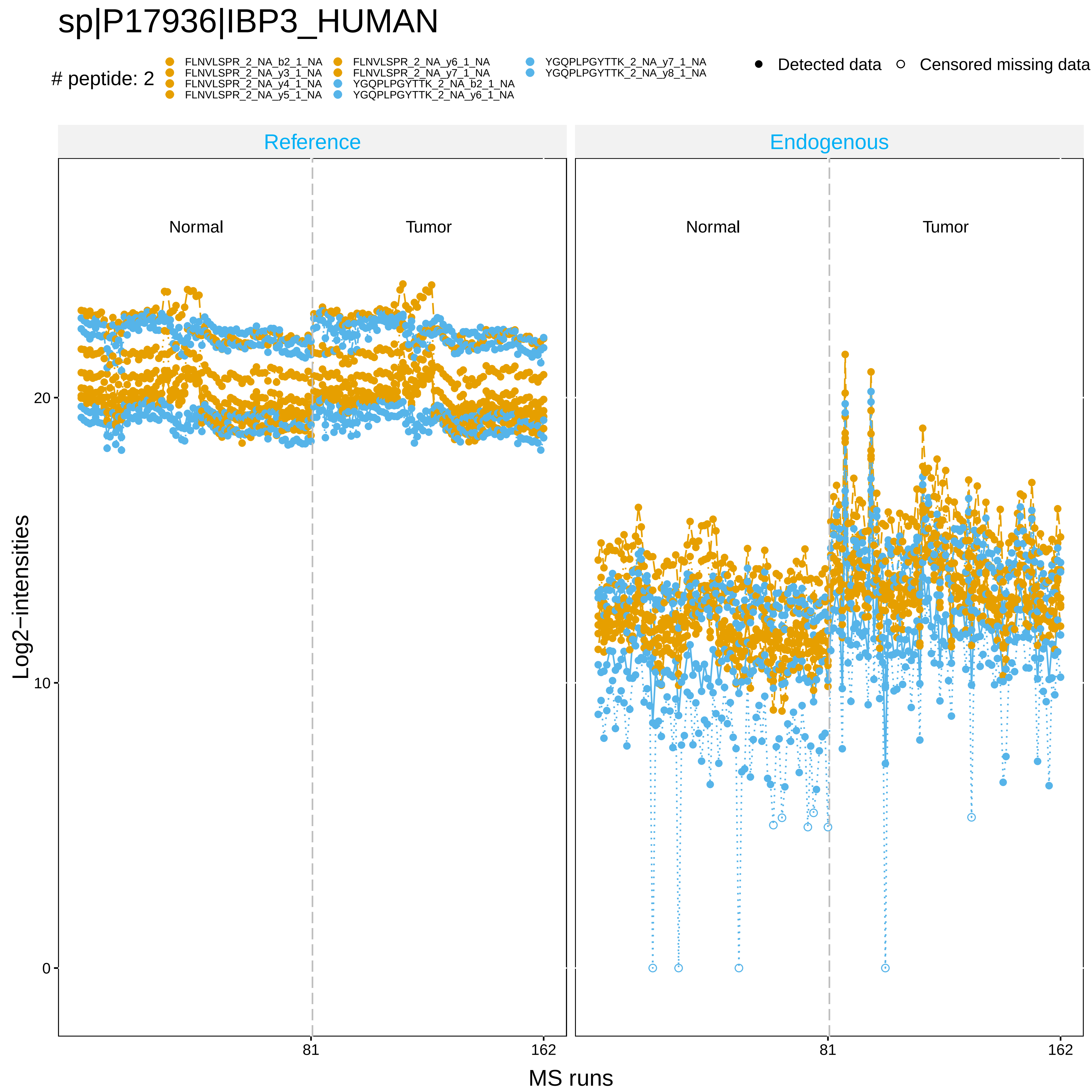

sp|P17936|IBP3_HUMAN
peptide: 2
FLNVLSPR_2_NA_b2_1_NA
FLNVLSPR_2_NA_y3_1_NA
FLNVLSPR_2_NA_y4_1_NA
FLNVLSPR_2_NA_y5_1_NA
FLNVLSPR_2_NA_y6_1_NA
FLNVLSPR_2_NA_y7_1_NA
YGQPLPGYTTK_2_NA_b2_1_NA
YGQPLPGYTTK_2_NA_y6_1_NA
YGQPLPGYTTK_2_NA_y7_1_NA
YGQPLPGYTTK_2_NA_y8_1_NA
Detected data
Censored missing data
Reference
Endogenous
Normal
Tumor
Normal
Tumor
Log2−intensities
20
10
0
81
162
81
162
MS runs

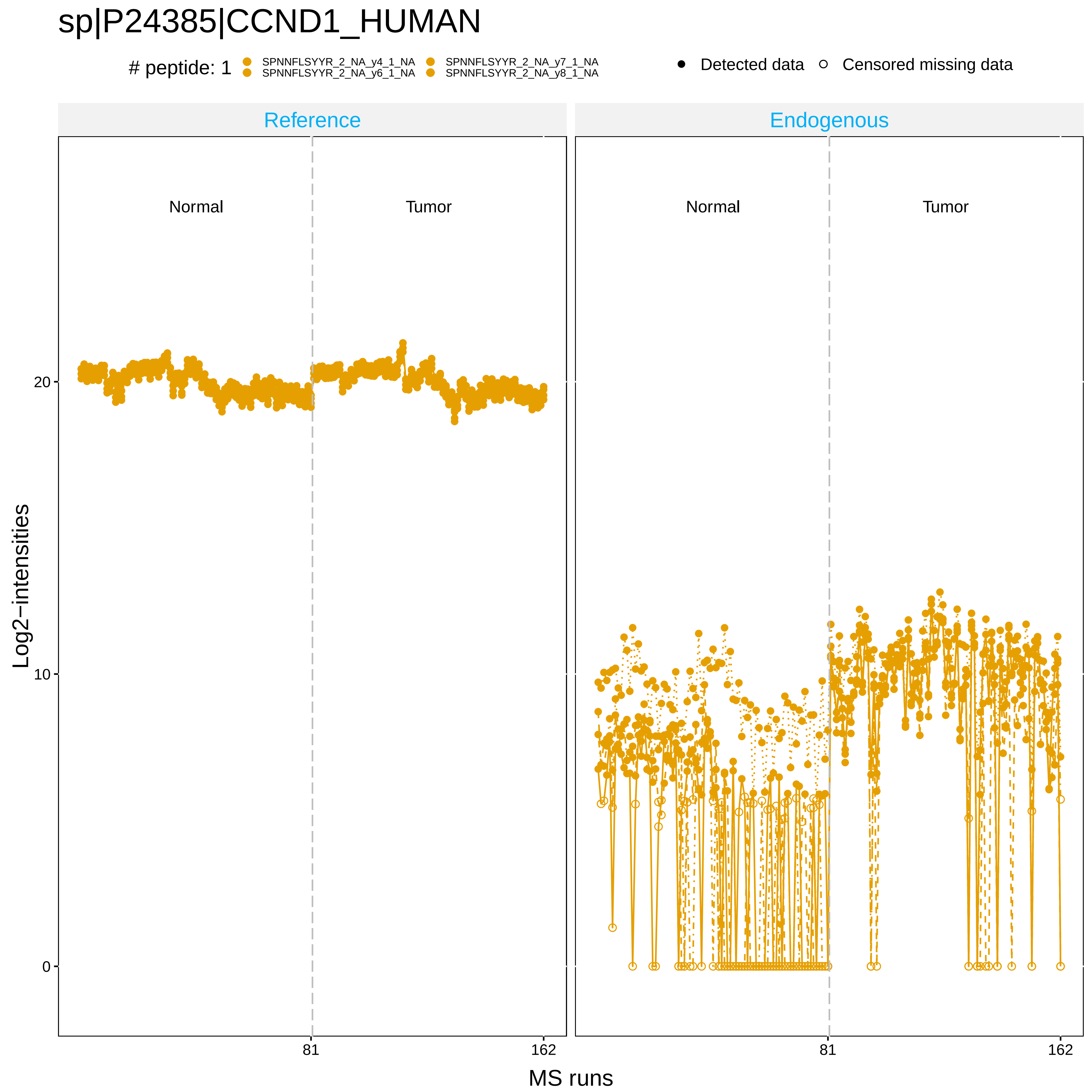

sp|P24385|CCND1_HUMAN
peptide: 1
SPNNFLSYYR_2_NA_y4_1_NA
SPNNFLSYYR_2_NA_y6_1_NA
SPNNFLSYYR_2_NA_y7_1_NA
SPNNFLSYYR_2_NA_y8_1_NA
Detected data
Censored missing data
Reference
Endogenous
Normal
Tumor
Normal
Tumor
Log2−intensities
20
10
0
81
162
81
162
MS runs

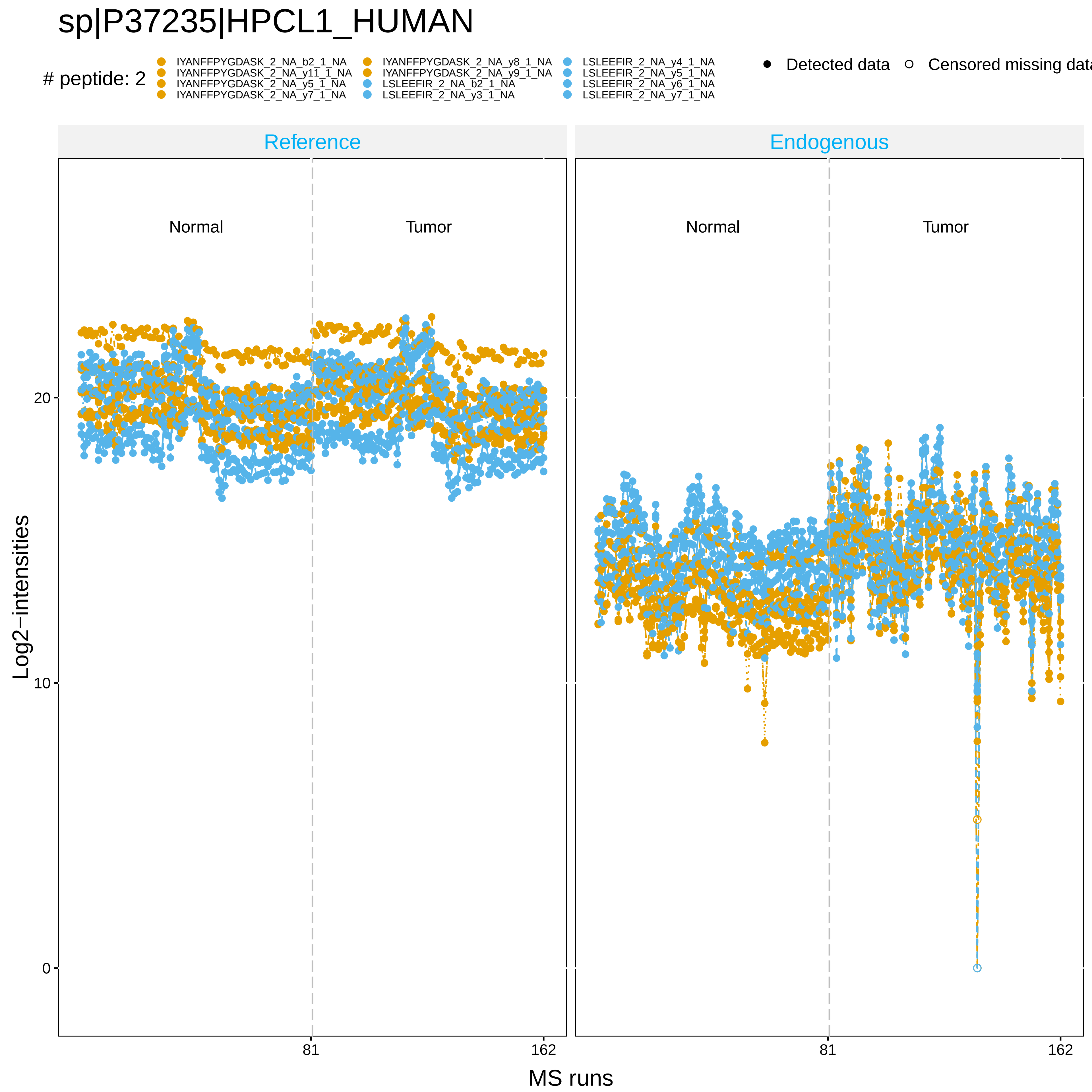
sp|P37235|HPCL1_HUMAN
peptide: 2
IYANFFPYGDASK_2_NA_b2_1_NA
IYANFFPYGDASK_2_NA_y11_1_NA
IYANFFPYGDASK_2_NA_y5_1_NA
IYANFFPYGDASK_2_NA_y7_1_NA
IYANFFPYGDASK_2_NA_y8_1_NA
IYANFFPYGDASK_2_NA_y9_1_NA
LSLEEFIR_2_NA_b2_1_NA
LSLEEFIR_2_NA_y3_1_NA
LSLEEFIR_2_NA_y4_1_NA
LSLEEFIR_2_NA_y5_1_NA
LSLEEFIR_2_NA_y6_1_NA
LSLEEFIR_2_NA_y7_1_NA
Detected data
Censored missing data
Reference
Endogenous
Normal
Tumor
Normal
Tumor
Log2−intensities
20
10
0
81
162
81
162
MS runs

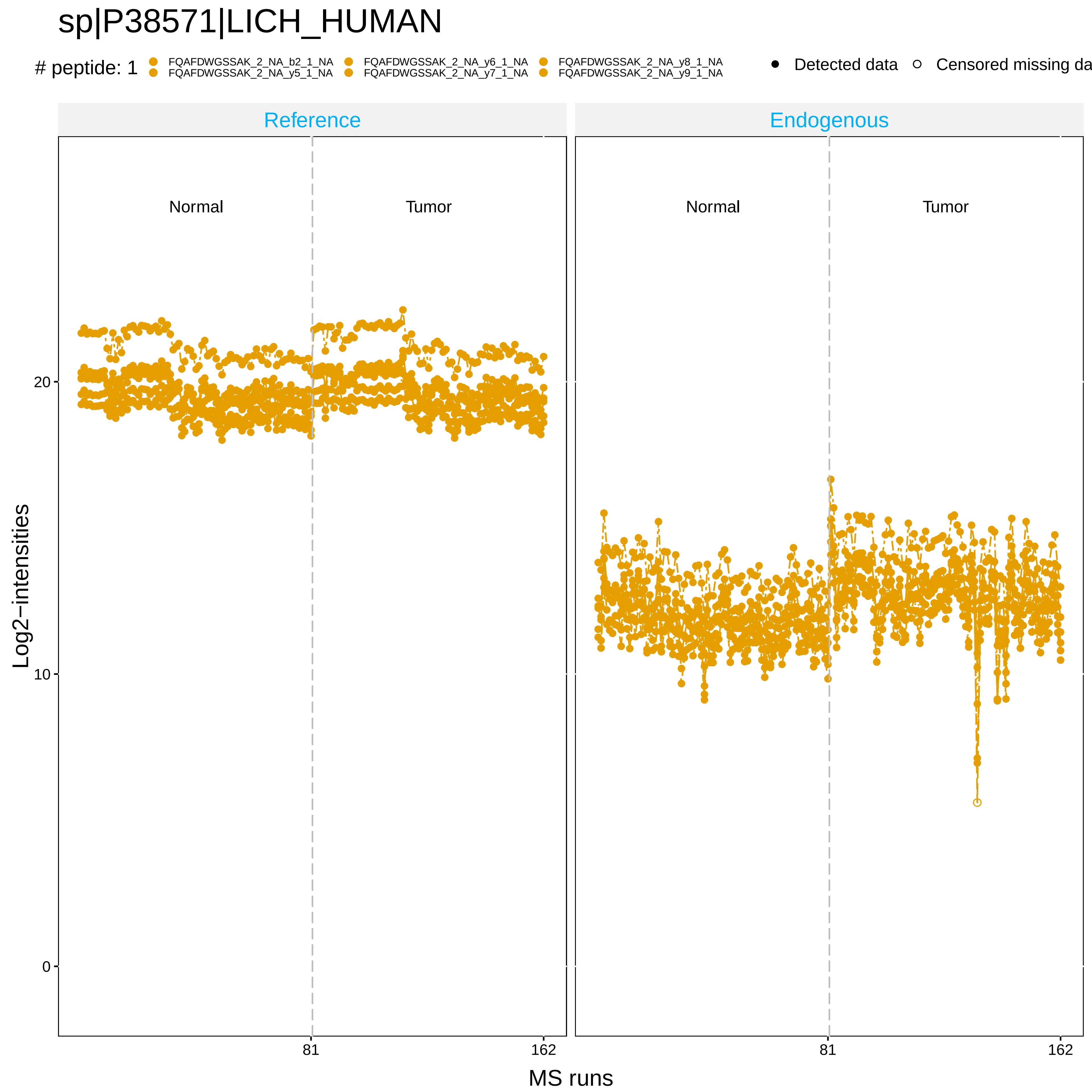

sp|P38571|LICH_HUMAN
peptide: 1
FQAFDWGSSAK_2_NA_b2_1_NA
FQAFDWGSSAK_2_NA_y5_1_NA
FQAFDWGSSAK_2_NA_y6_1_NA
FQAFDWGSSAK_2_NA_y7_1_NA
FQAFDWGSSAK_2_NA_y8_1_NA
FQAFDWGSSAK_2_NA_y9_1_NA
Detected data
Censored missing da
Reference
Endogenous
Normal
Tumor
Normal
Tumor
Log2−intensities
20
10
0
81
162
81
162
MS runs

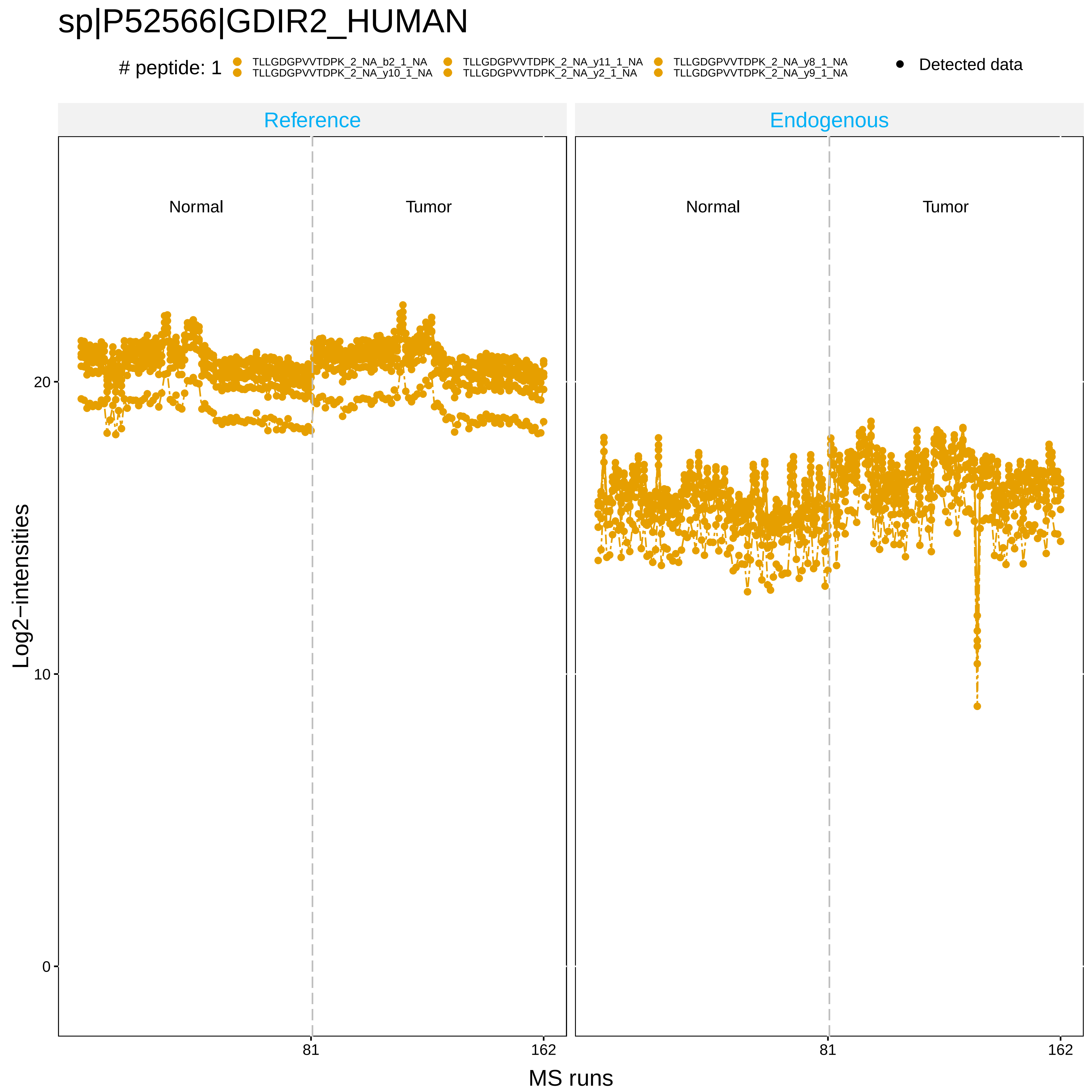
sp|P52566|GDIR2_HUMAN
peptide: 1
TLLGDGPVVTDPK_2_NA_b2_1_NA
TLLGDGPVVTDPK_2_NA_y10_1_NA
TLLGDGPVVTDPK_2_NA_y11_1_NA
TLLGDGPVVTDPK_2_NA_y2_1_NA
TLLGDGPVVTDPK_2_NA_y8_1_NA
TLLGDGPVVTDPK_2_NA_y9_1_NA
Detected data
Reference
Endogenous
Normal
Tumor
Normal
Tumor
Log2−intensities
20
10
0
81
162
81
162
MS runs

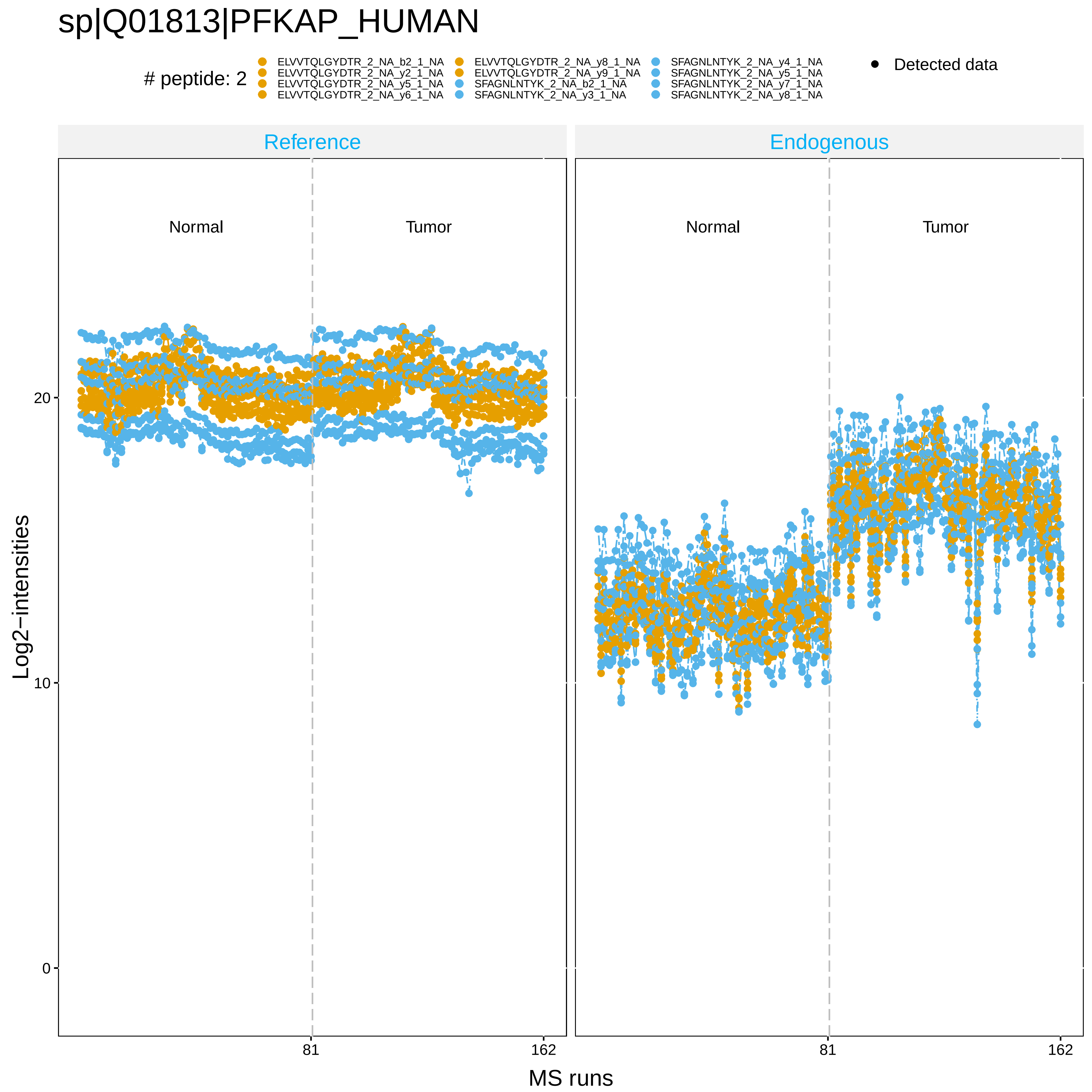

sp|Q01813|PFKAP_HUMAN
peptide: 2
ELVVTQLGYDTR_2_NA_b2_1_NA
ELVVTQLGYDTR_2_NA_y2_1_NA
ELVVTQLGYDTR_2_NA_y5_1_NA
ELVVTQLGYDTR_2_NA_y6_1_NA
ELVVTQLGYDTR_2_NA_y8_1_NA
ELVVTQLGYDTR_2_NA_y9_1_NA
SFAGNLNTYK_2_NA_b2_1_NA
SFAGNLNTYK_2_NA_y3_1_NA
SFAGNLNTYK_2_NA_y4_1_NA
SFAGNLNTYK_2_NA_y5_1_NA
SFAGNLNTYK_2_NA_y7_1_NA
SFAGNLNTYK_2_NA_y8_1_NA
Detected data
Reference
Endogenous
Normal
Tumor
Normal
Tumor
Log2−intensities
20
10
0
81
162
81
162
MS runs

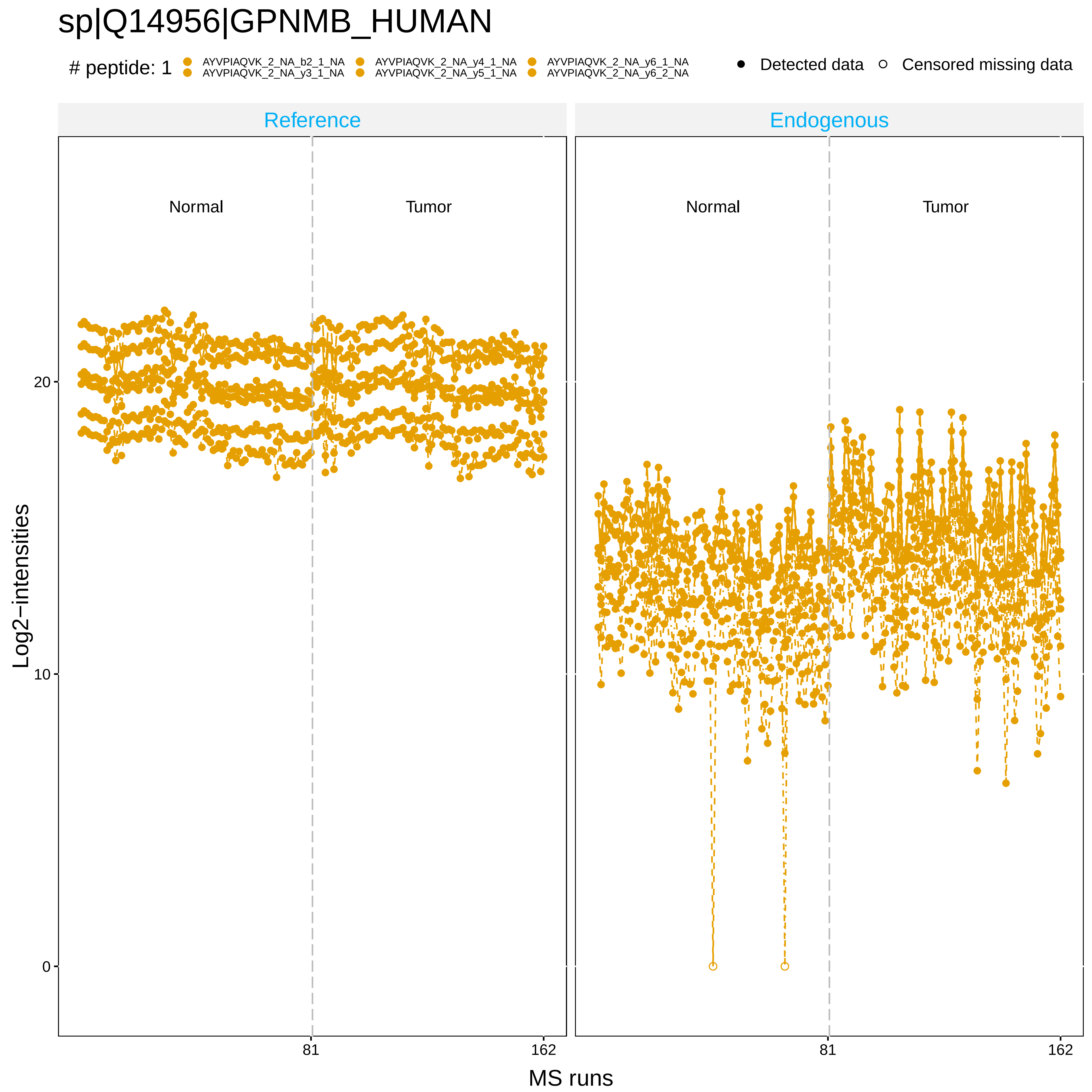

sp|Q14956|GPNMB_HUMAN
peptide: 1
AYVPIAQVK_2_NA_b2_1_NA
AYVPIAQVK_2_NA_y3_1_NA
AYVPIAQVK_2_NA_y4_1_NA
AYVPIAQVK_2_NA_y5_1_NA
AYVPIAQVK_2_NA_y6_1_NA
AYVPIAQVK_2_NA_y6_2_NA
Detected data
Censored missing data
Reference
Endogenous
Normal
Tumor
Normal
Tumor
Log2−intensities
20
10
0
81
162
81
162
MS runs

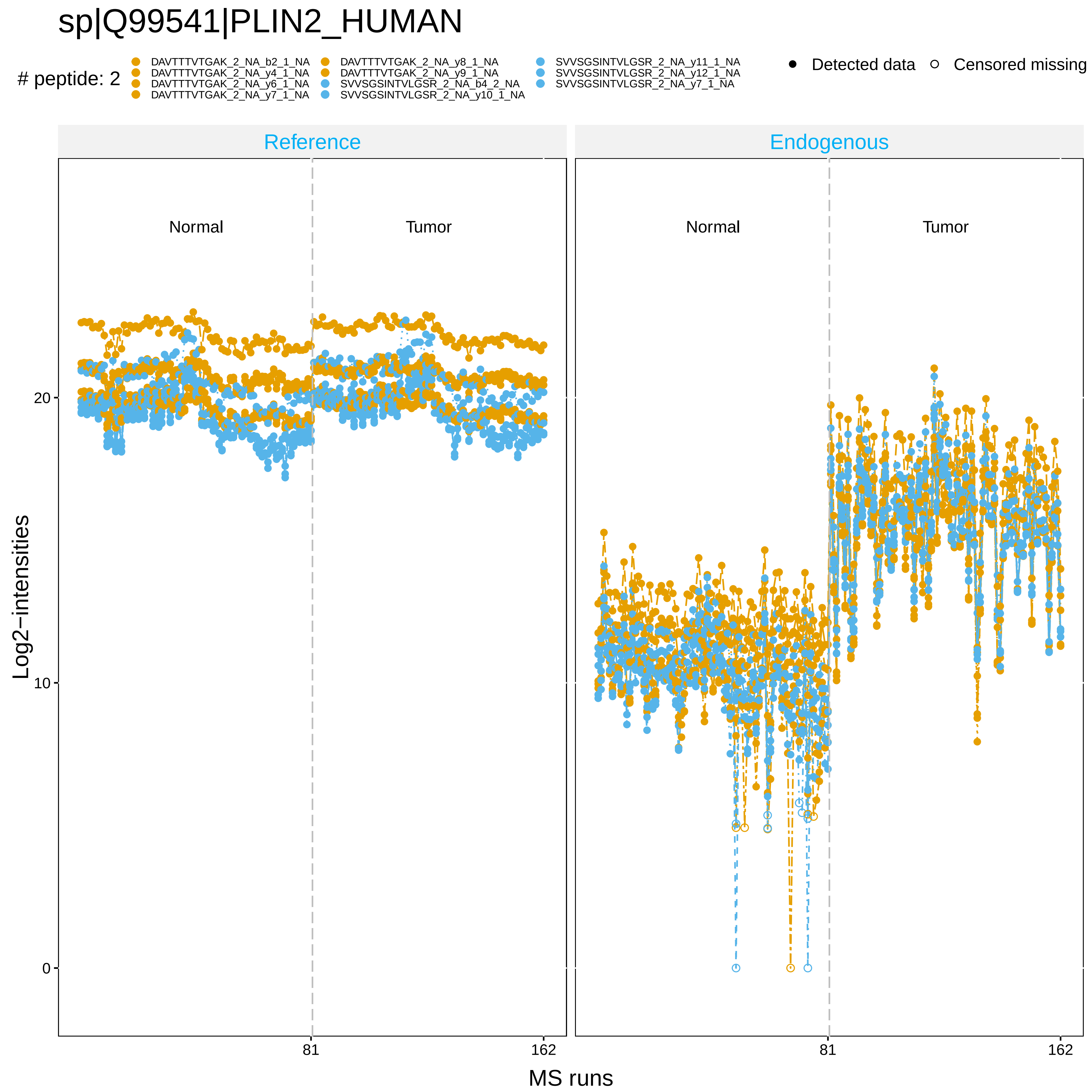
sp|Q99541|PLIN2_HUMAN
peptide: 2
DAVTTTVTGAK_2_NA_b2_1_NA
DAVTTTVTGAK_2_NA_y4_1_NA
DAVTTTVTGAK_2_NA_y6_1_NA
DAVTTTVTGAK_2_NA_y7_1_NA
DAVTTTVTGAK_2_NA_y8_1_NA
DAVTTTVTGAK_2_NA_y9_1_NA
SVVSGSINTVLGSR_2_NA_b4_2_NA
SVVSGSINTVLGSR_2_NA_y10_1_NA
SVVSGSINTVLGSR_2_NA_y11_1_NA
SVVSGSINTVLGSR_2_NA_y12_1_NA
SVVSGSINTVLGSR_2_NA_y7_1_NA
Detected data
Censored missing
Reference
Endogenous
Normal
Tumor
Normal
Tumor
Log2−intensities
20
10
0
81
162
81
162
MS runs

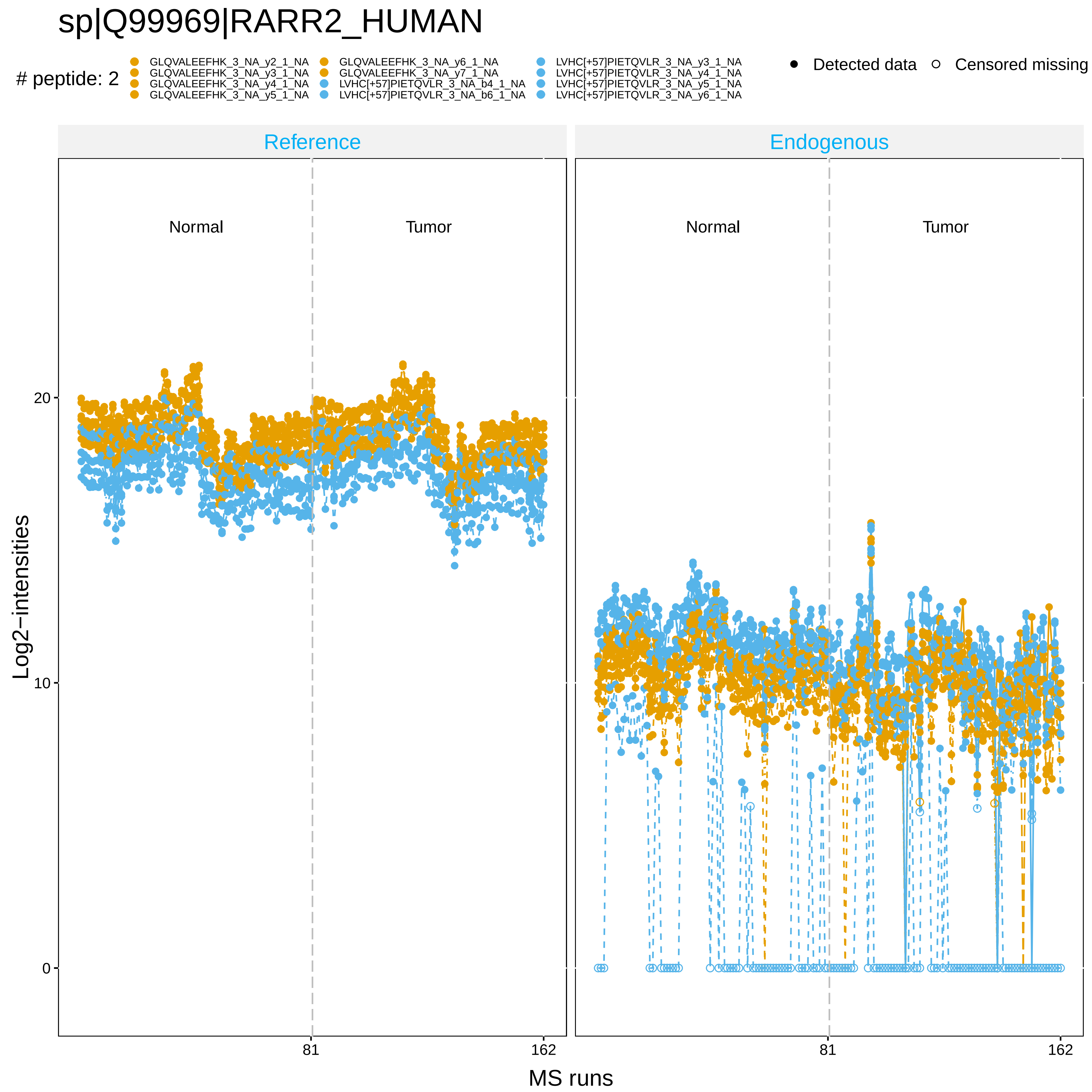

sp|Q99969|RARR2_HUMAN
peptide: 2
GLQVALEEFHK_3_NA_y2_1_NA
GLQVALEEFHK_3_NA_y3_1_NA
GLQVALEEFHK_3_NA_y4_1_NA
GLQVALEEFHK_3_NA_y5_1_NA
GLQVALEEFHK_3_NA_y6_1_NA
GLQVALEEFHK_3_NA_y7_1_NA
LVHC[+57]PIETQVLR_3_NA_b4_1_NA
LVHC[+57]PIETQVLR_3_NA_b6_1_NA
LVHC[+57]PIETQVLR_3_NA_y3_1_NA
LVHC[+57]PIETQVLR_3_NA_y4_1_NA
LVHC[+57]PIETQVLR_3_NA_y5_1_NA
LVHC[+57]PIETQVLR_3_NA_y6_1_NA
Detected data
Censored missing
Reference
Endogenous
Normal
Tumor
Log2−intensities
20
10
0
81
162
MS runs

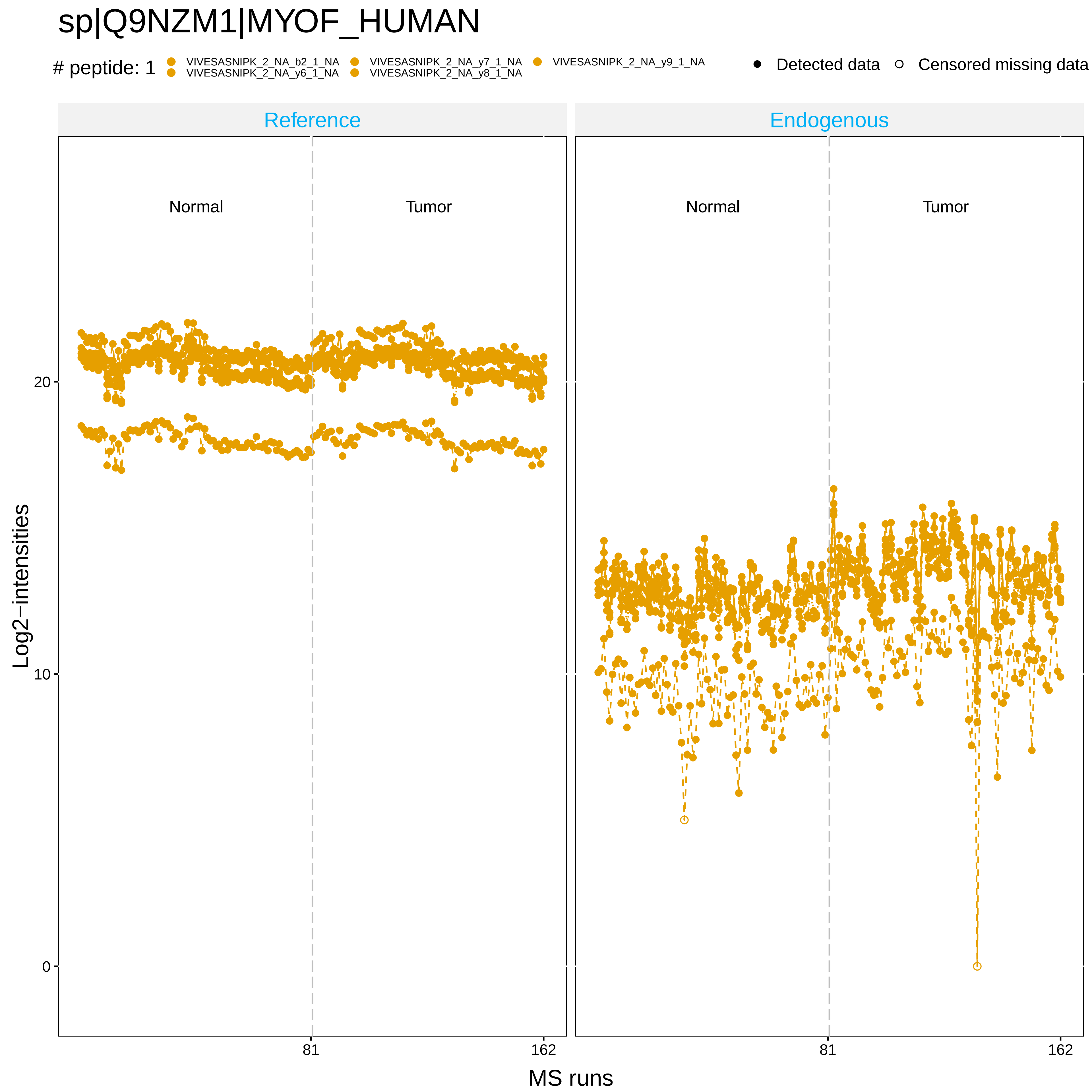

sp|Q9NZM1|MYOF_HUMAN
peptide: 1
VIVESASNIPK_2_NA_b2_1_NA
VIVESASNIPK_2_NA_y6_1_NA
VIVESASNIPK_2_NA_y7_1_NA
VIVESASNIPK_2_NA_y8_1_NA
VIVESASNIPK_2_NA_y9_1_NA
Detected data
Censored missing data
Reference
Endogenous
Normal
Tumor
Normal
Tumor
Log2−intensities
20
10
0
81
162
81
162
MS runs
