# Supplementary material for: Proteotranscriptomic Discrimination of Tumor and Normal Tissues in Renal Cell Carcinoma
Source: Int J Mol Sci. 2023 Feb 24;24(5):4488. doi: 10.3390/ijms24054488 (PMC10003397; doi:10.3390/ijms24054488)
Supplement: Supplementary file 1 [file ijms-24-04488-s001.zip › Results_Supplemental Figure S3.docx]

**Results_Supplemental Figure S3**

Summary dot plot of classification accuracy and Kappa values in the training cohorts by multiple methods (A). The accuracy of SVM (B), KNN (C), RF (D), and LOGIT (E) methods when using different sets of variables.

| **A** | **B** |
| --- | --- |
| **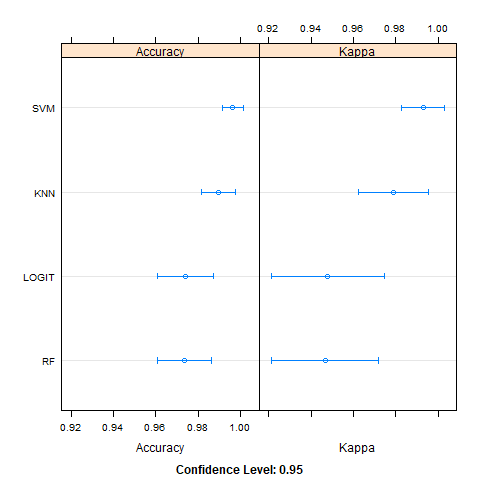** | **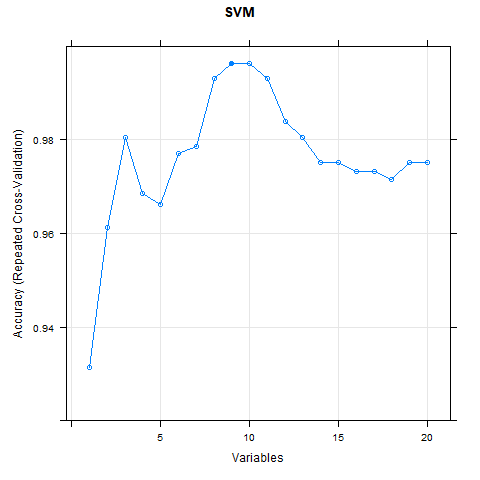** |
| **C** | **D** |
| **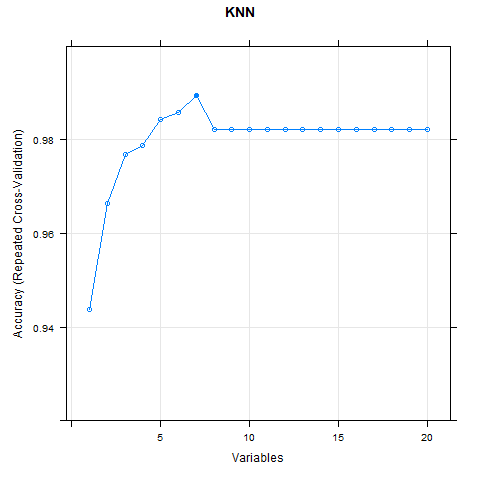** | **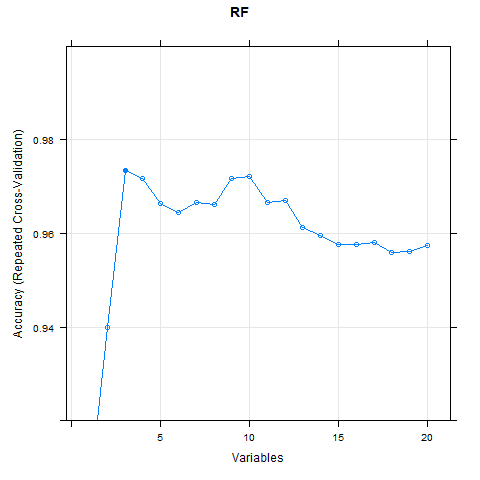** |
| **E** |  |
| **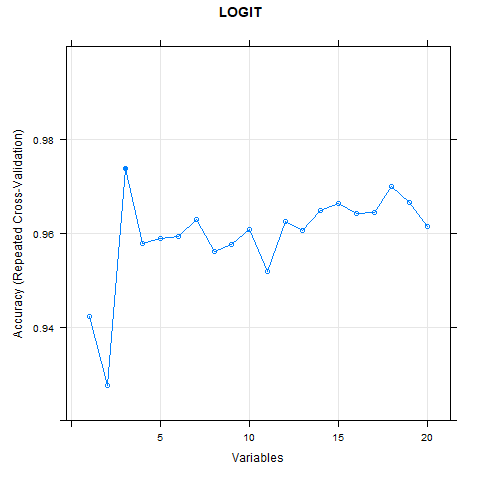** |  |
