# Supplementary figures and images for: Proteotranscriptomic Discrimination of Tumor and Normal Tissues in Renal Cell Carcinoma
Source: Int J Mol Sci. 2023 Feb 24;24(5):4488. doi: 10.3390/ijms24054488 (PMC10003397; doi:10.3390/ijms24054488)

Gender Plots


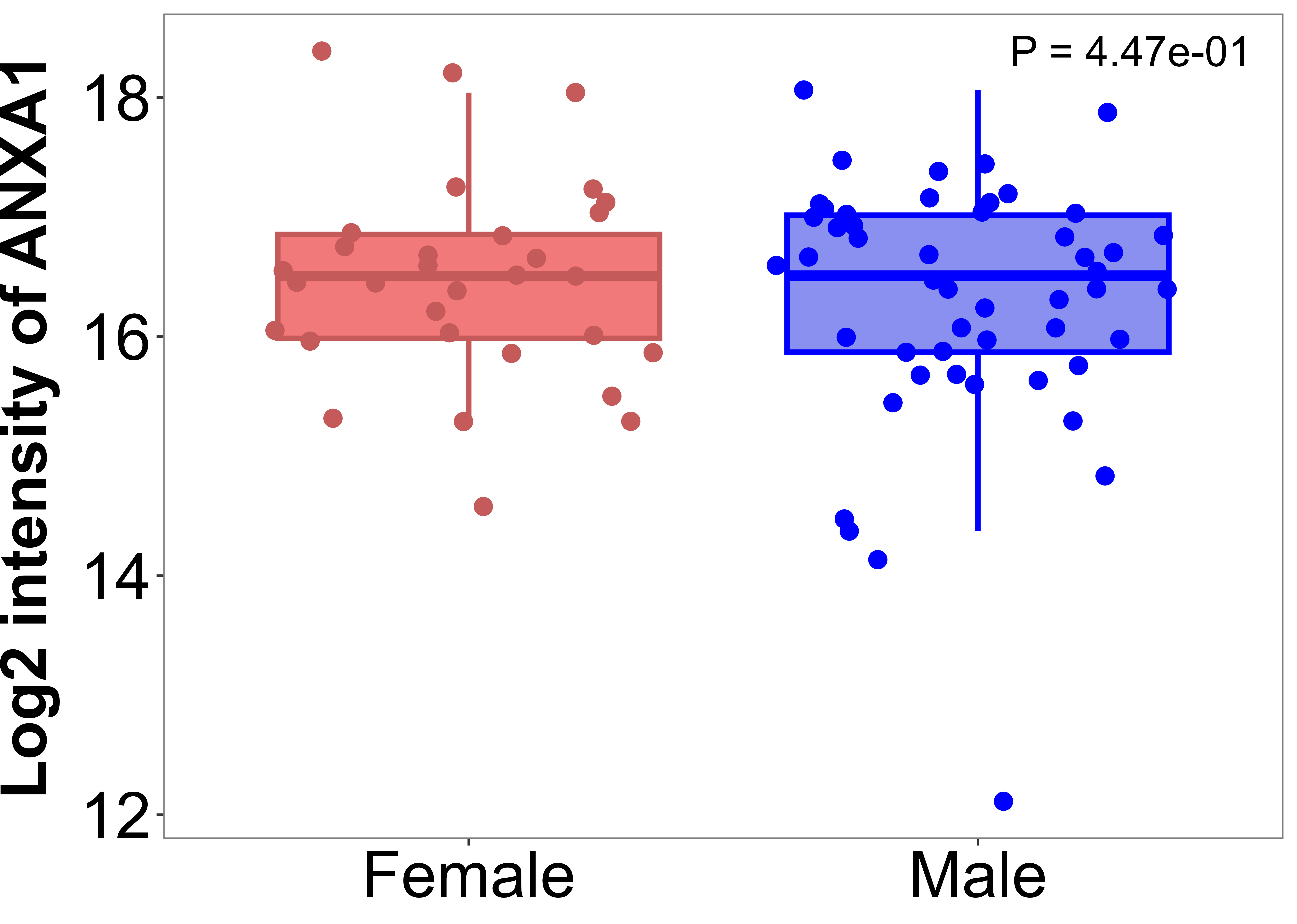

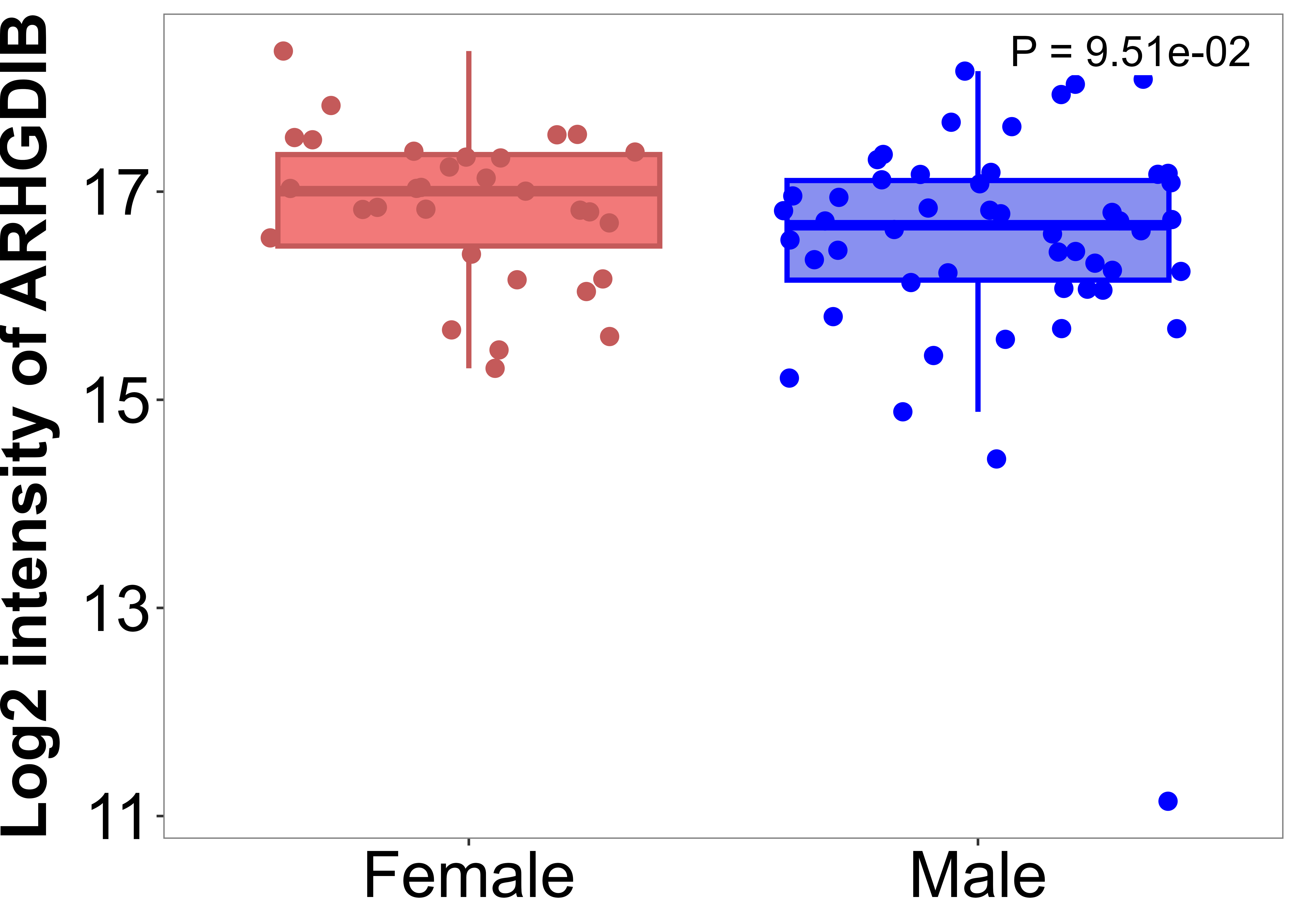

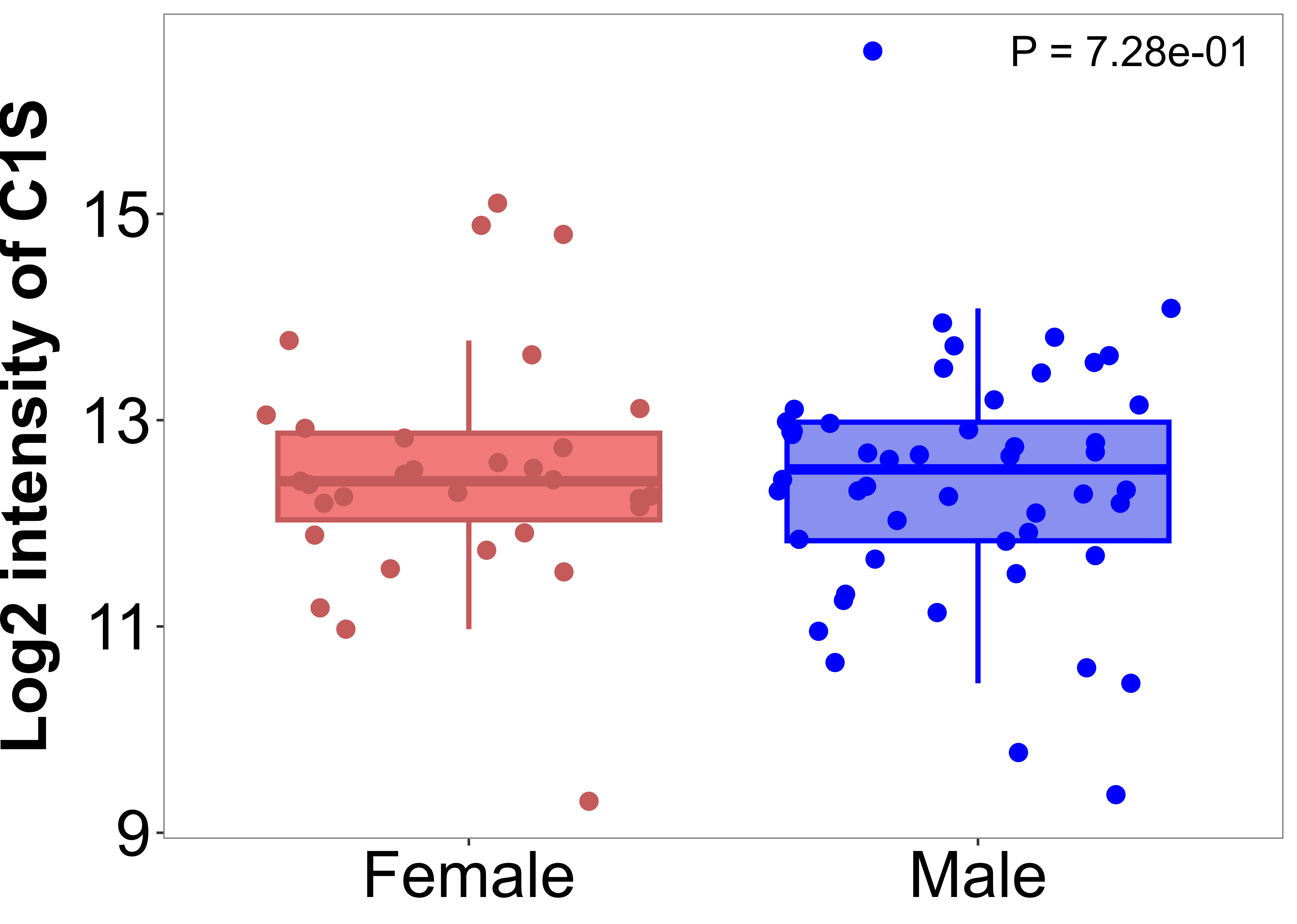

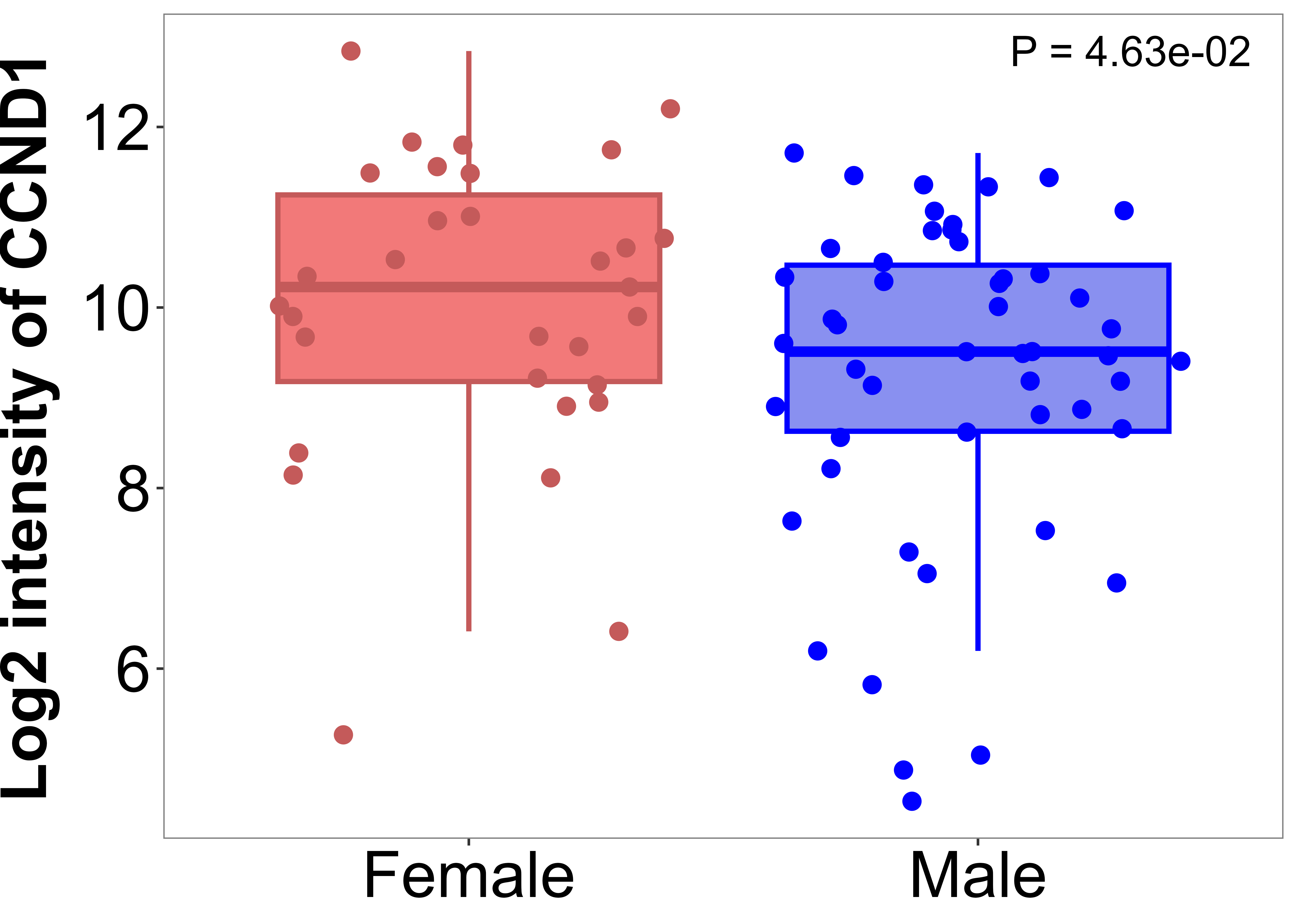

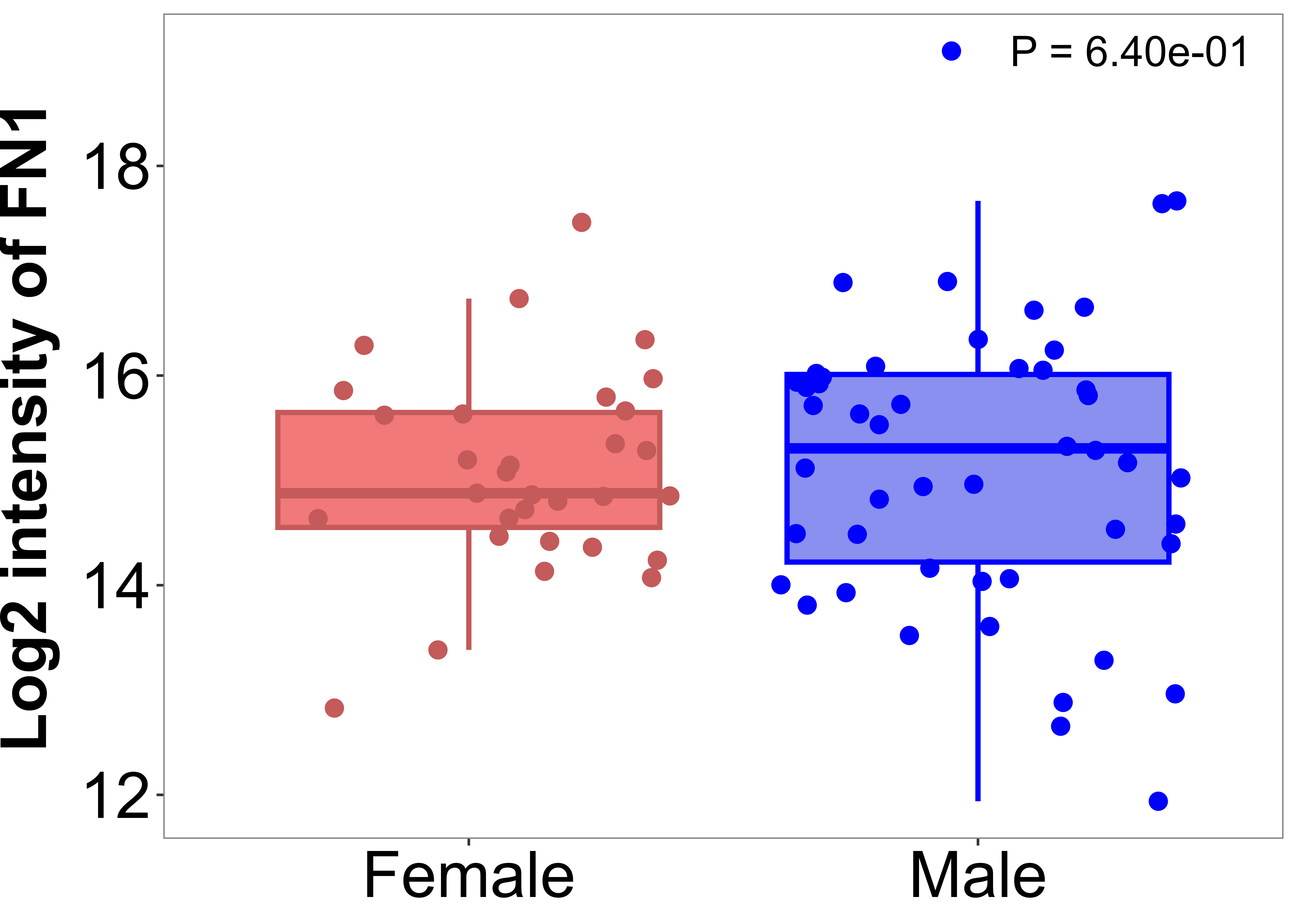

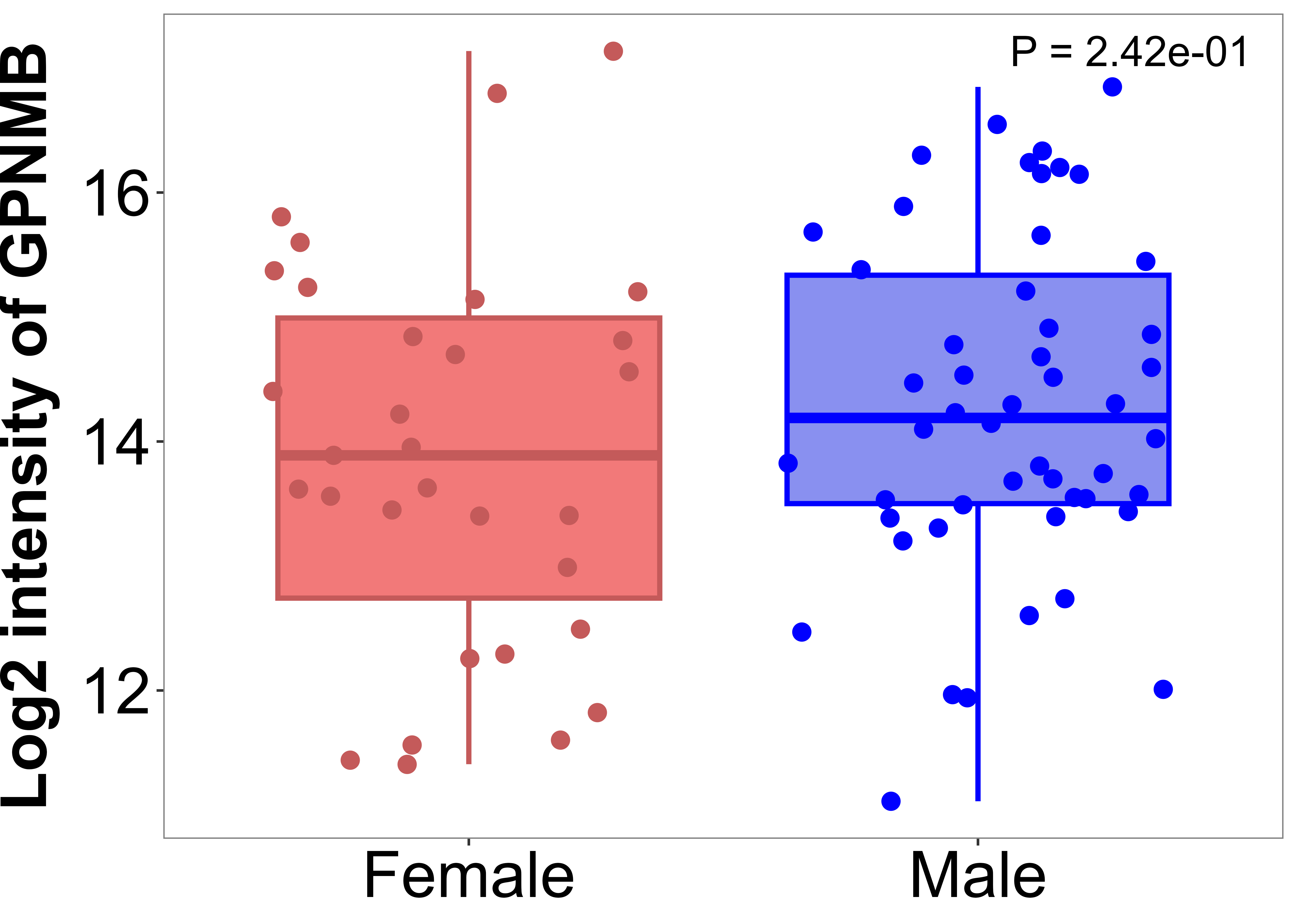

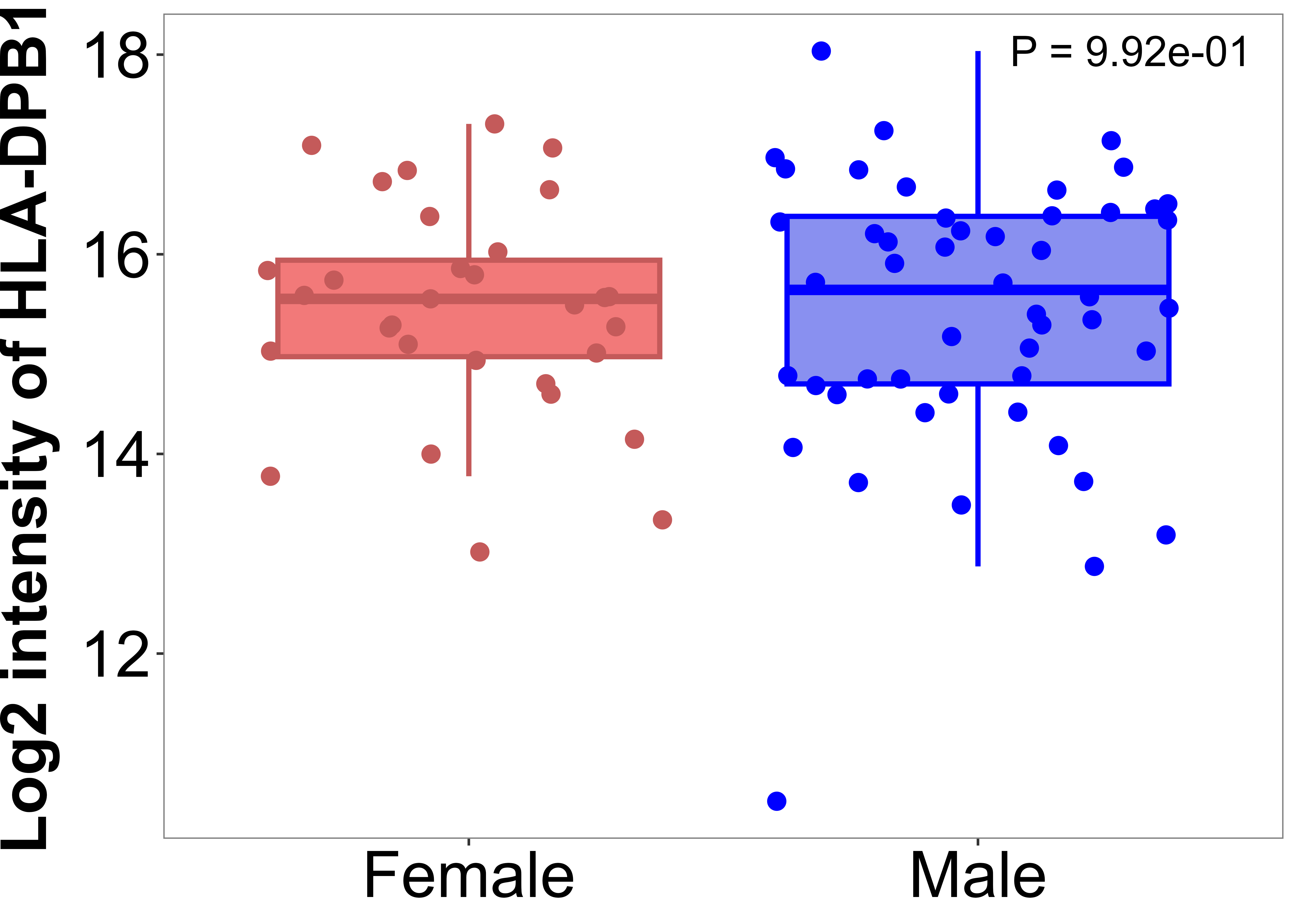

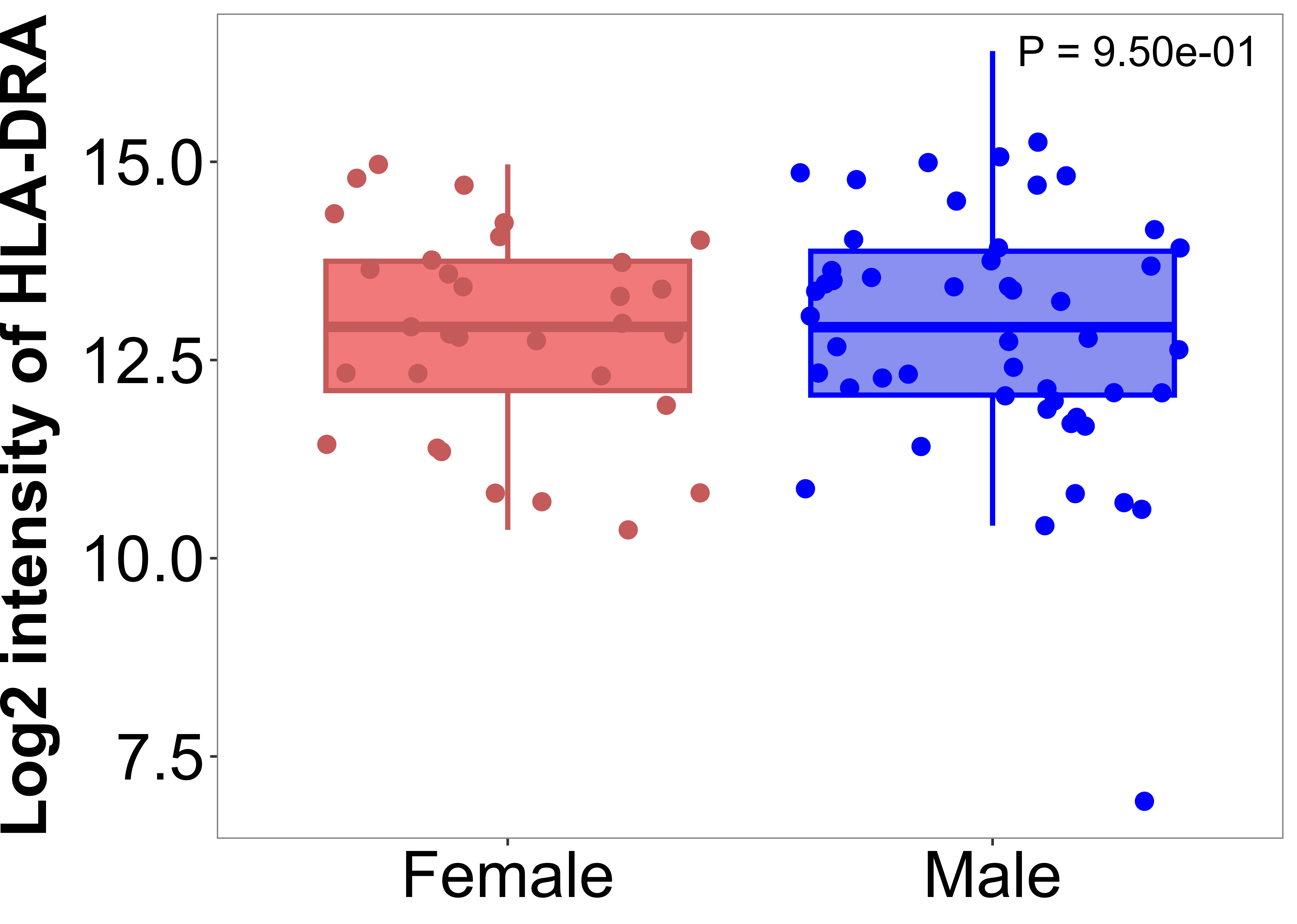

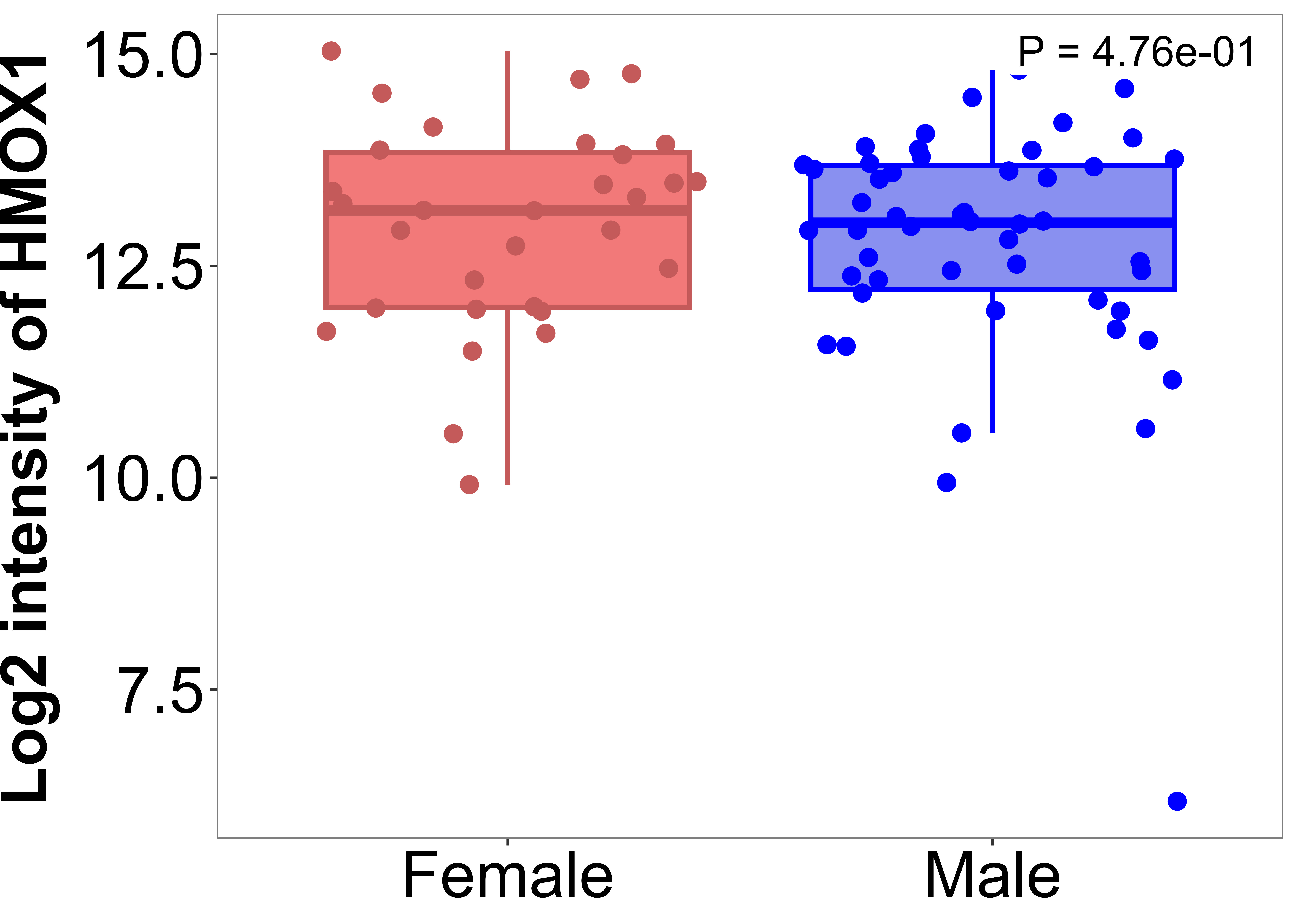

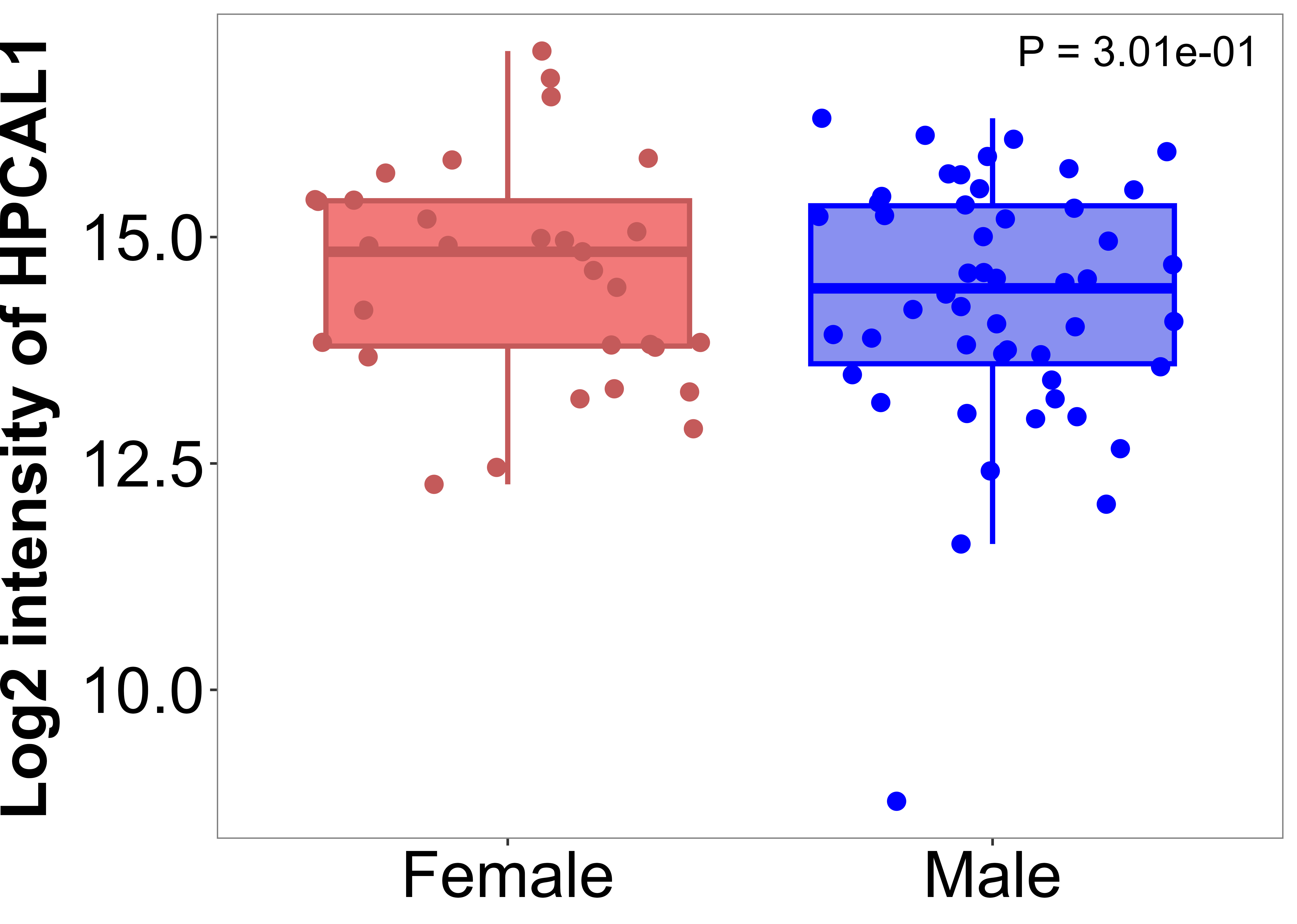

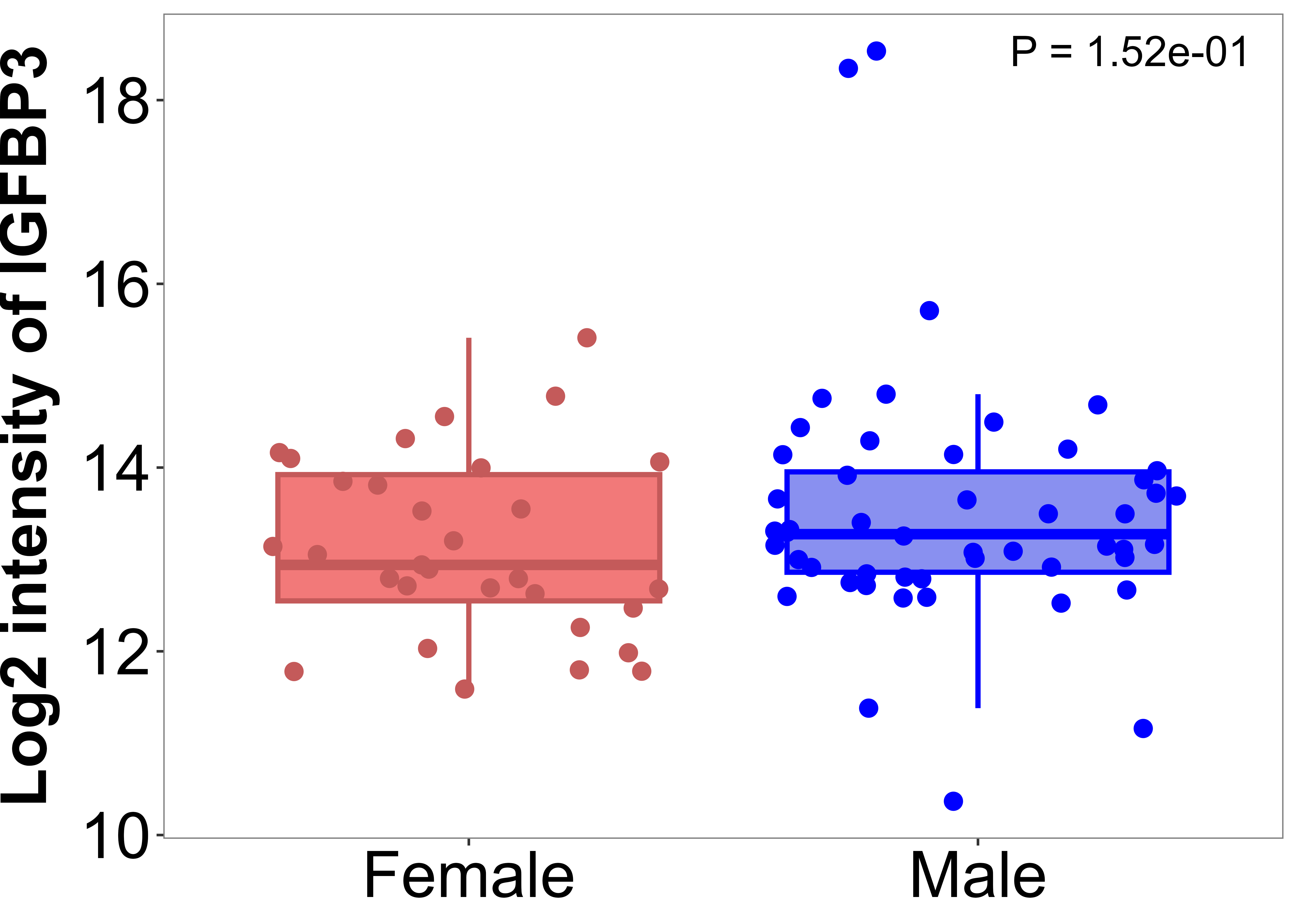

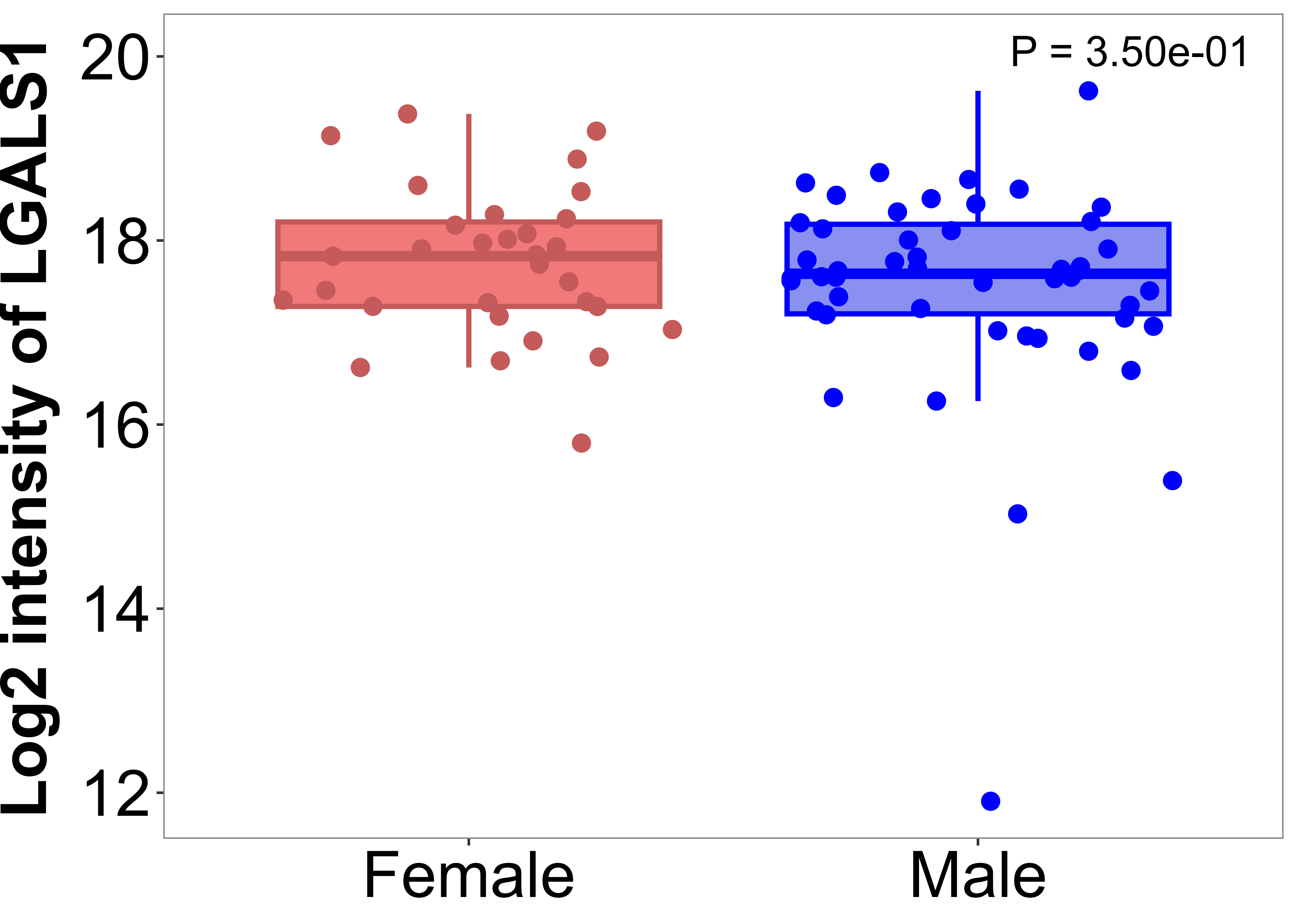

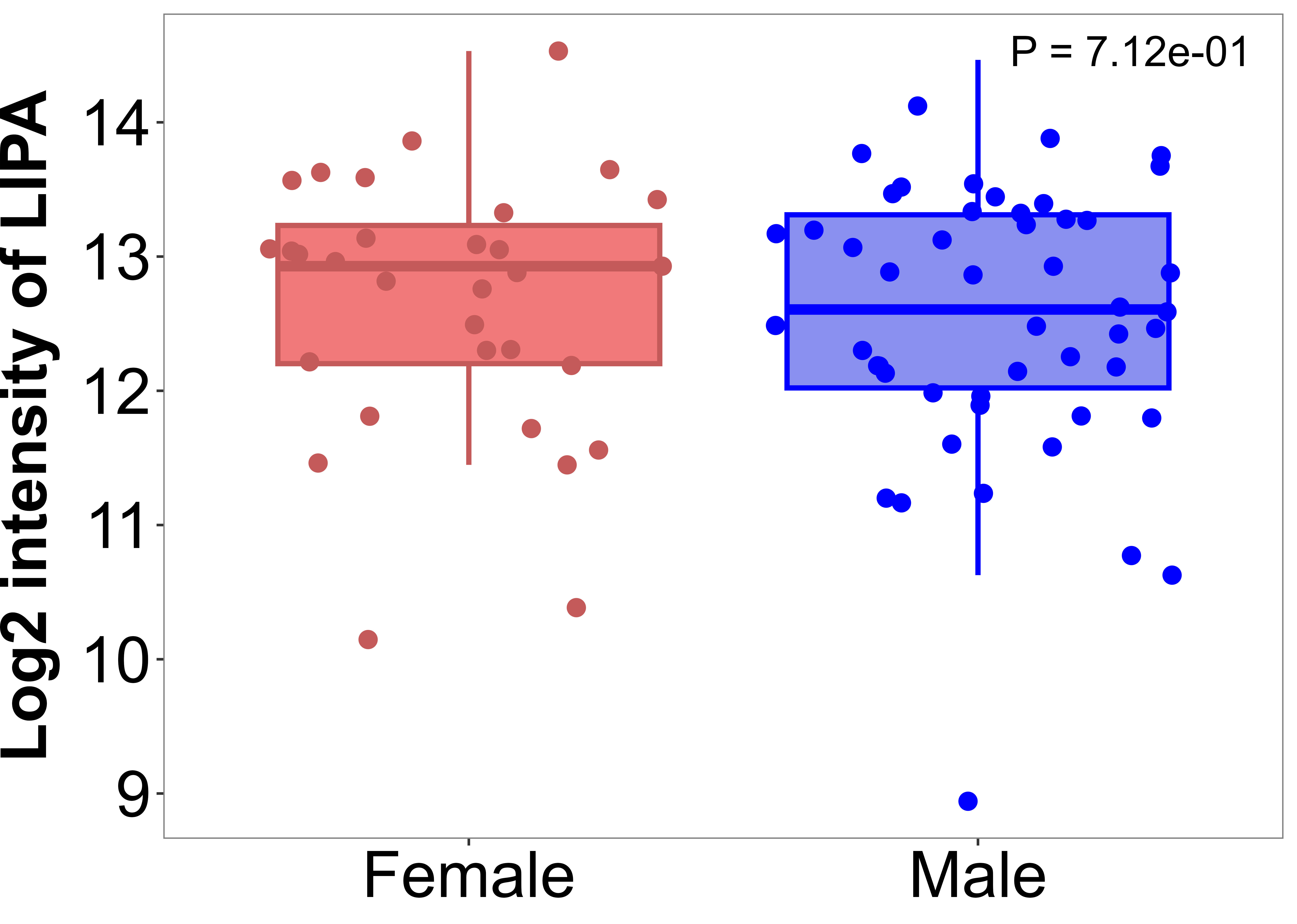

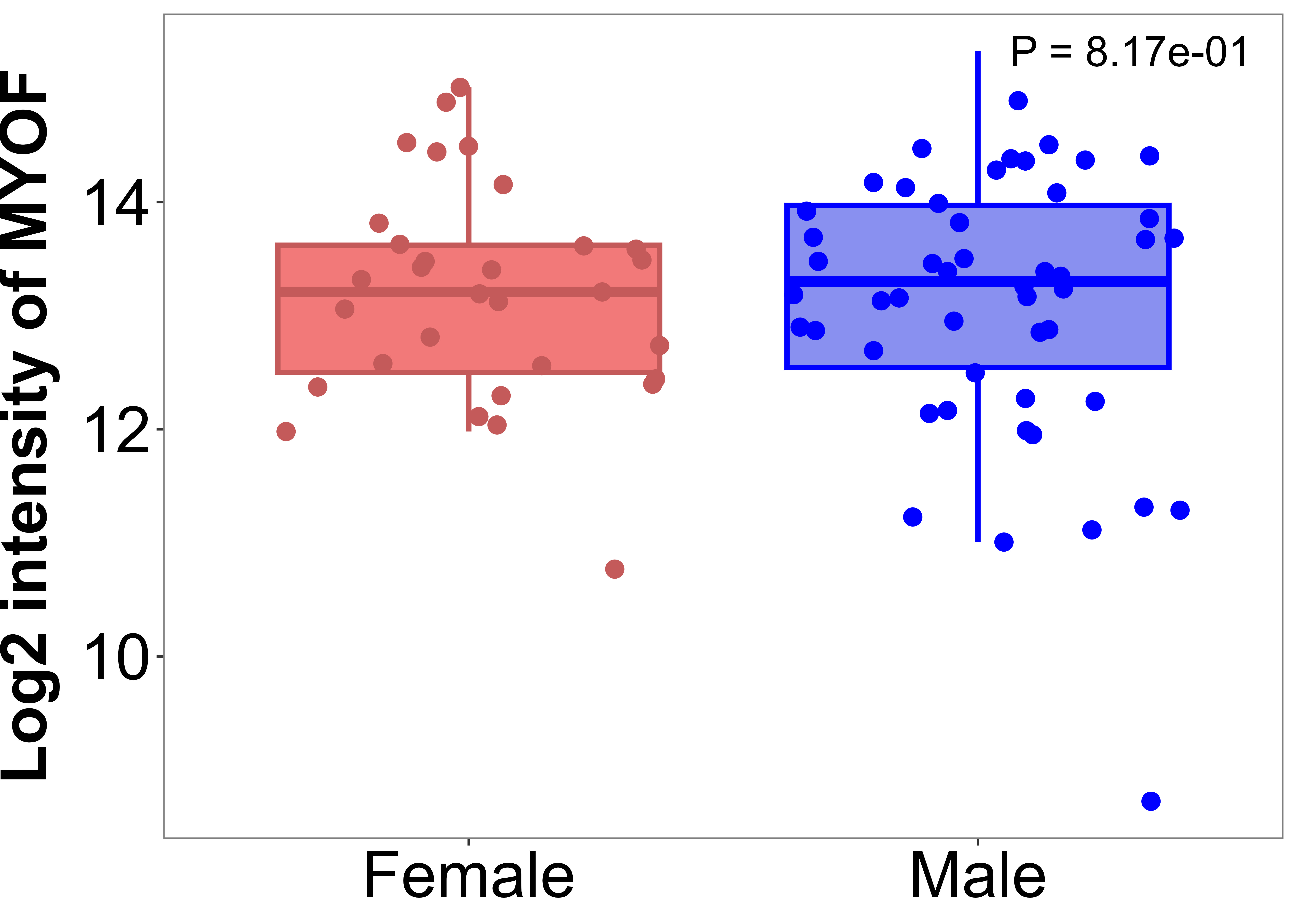

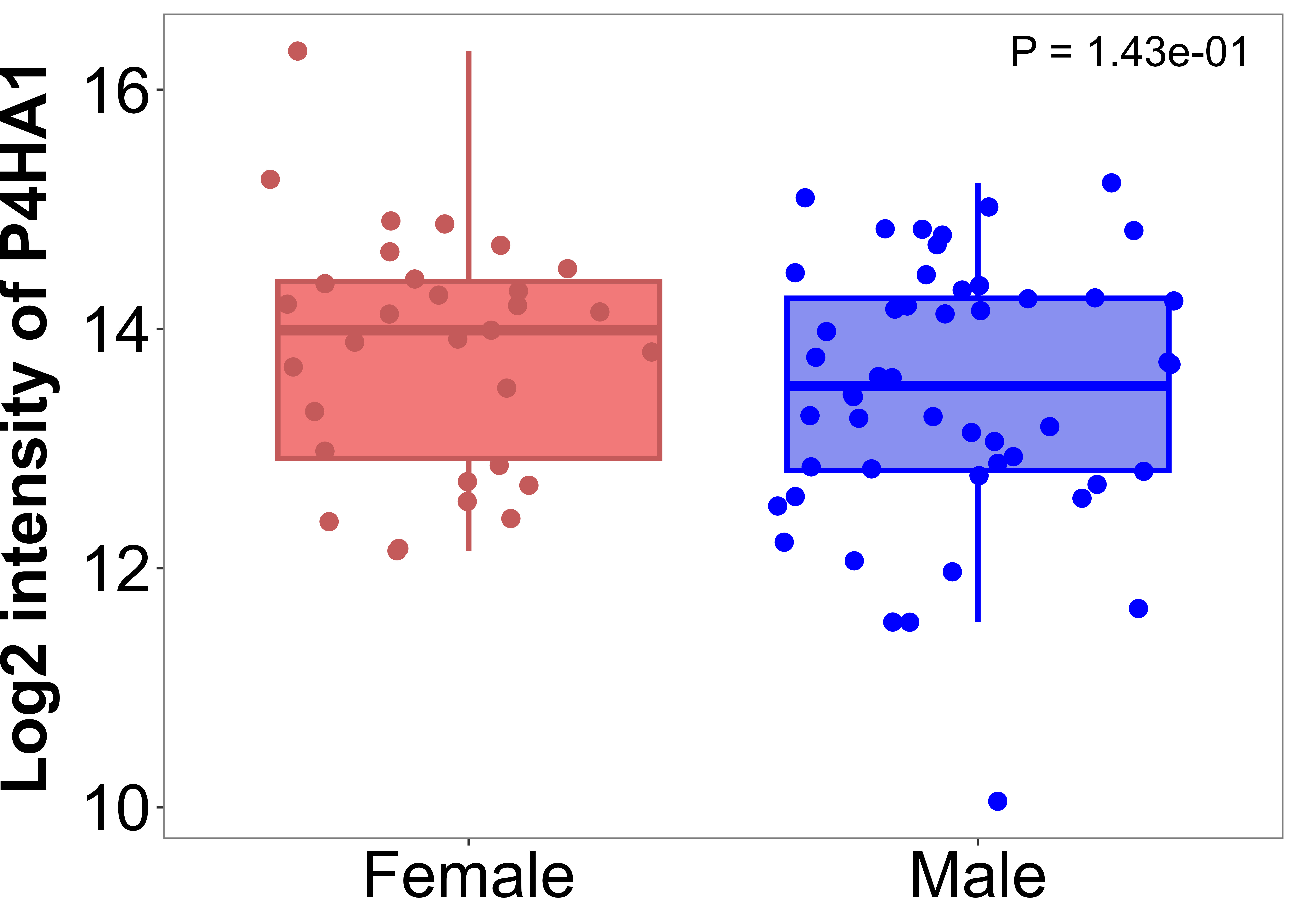

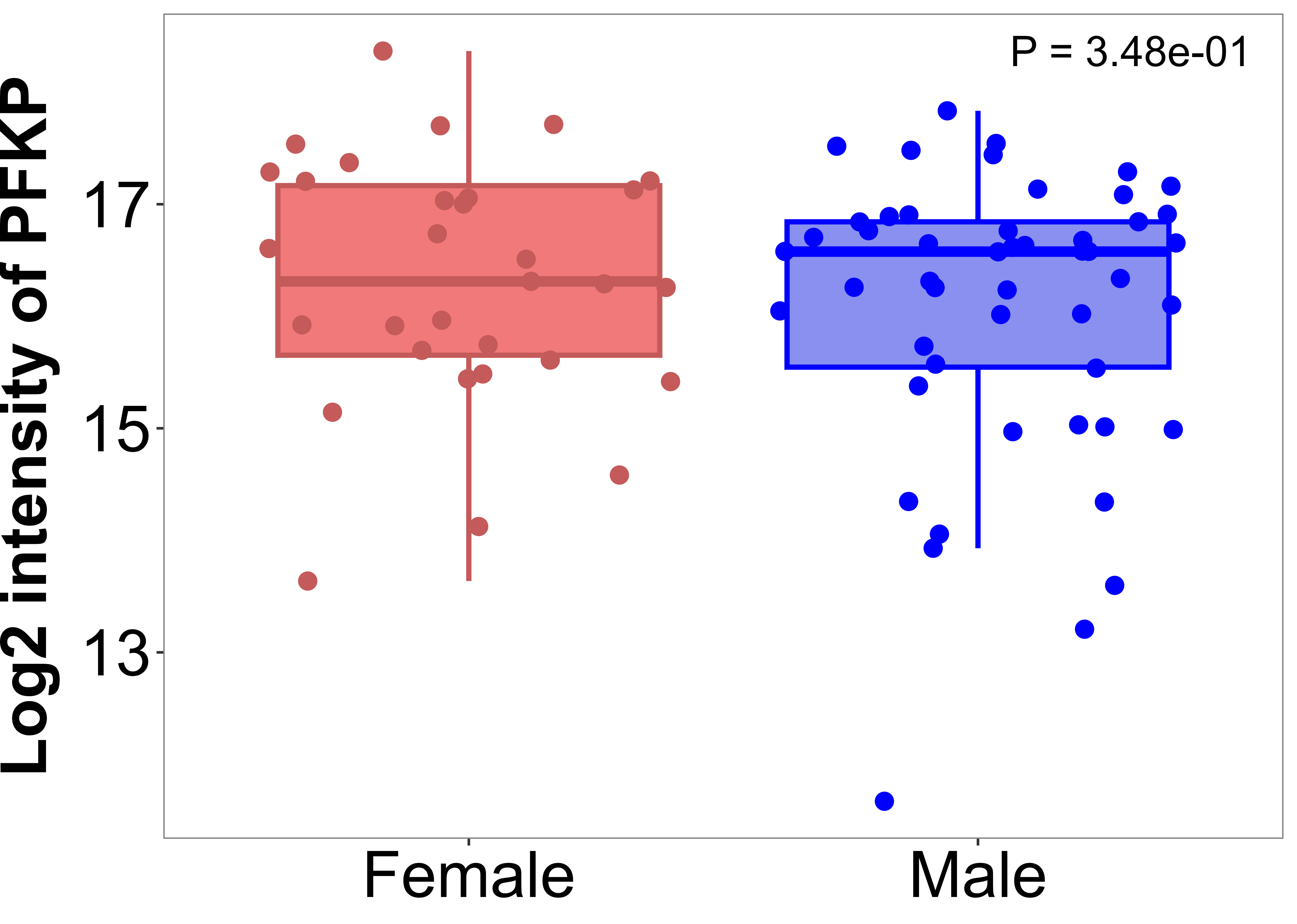

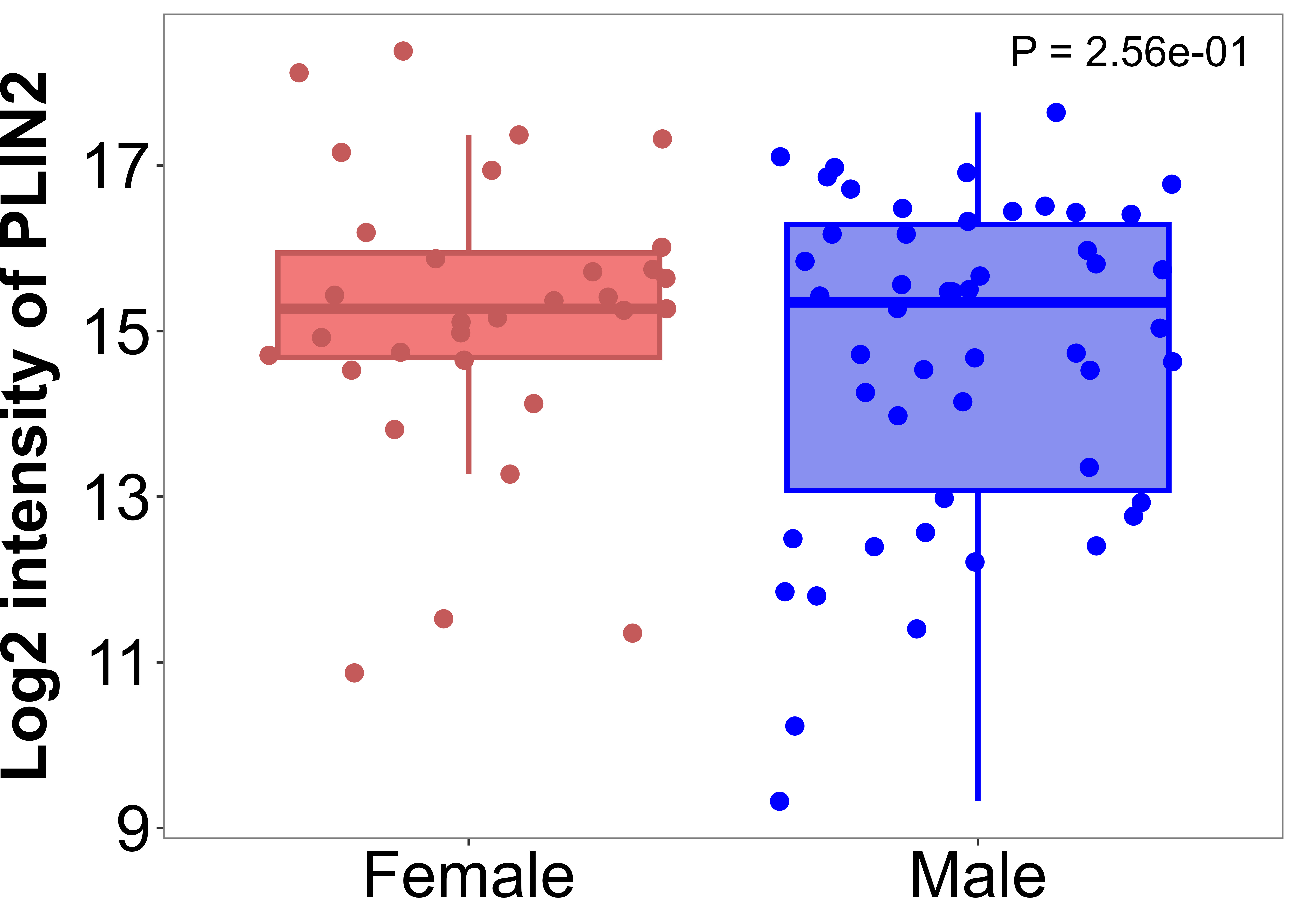

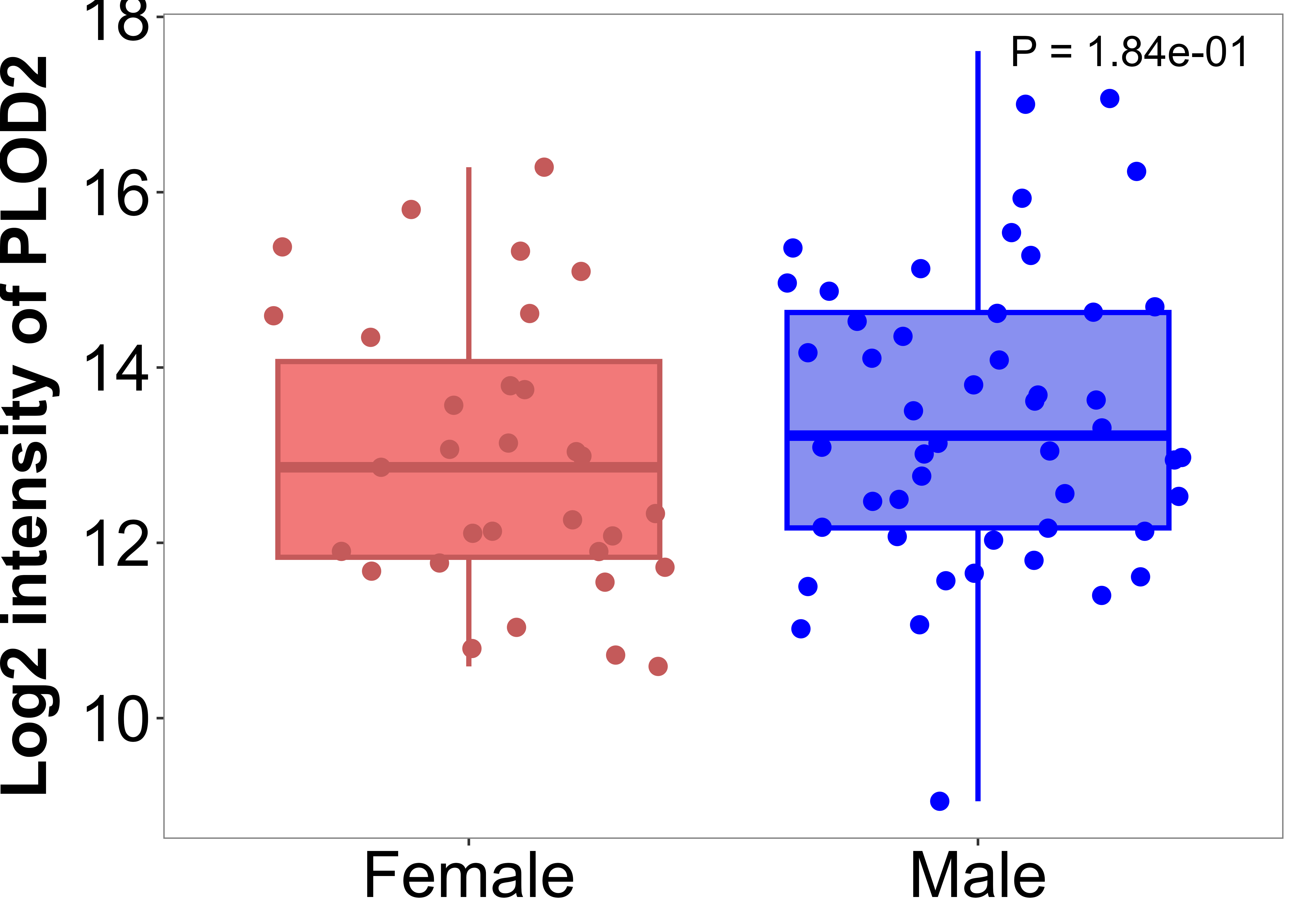

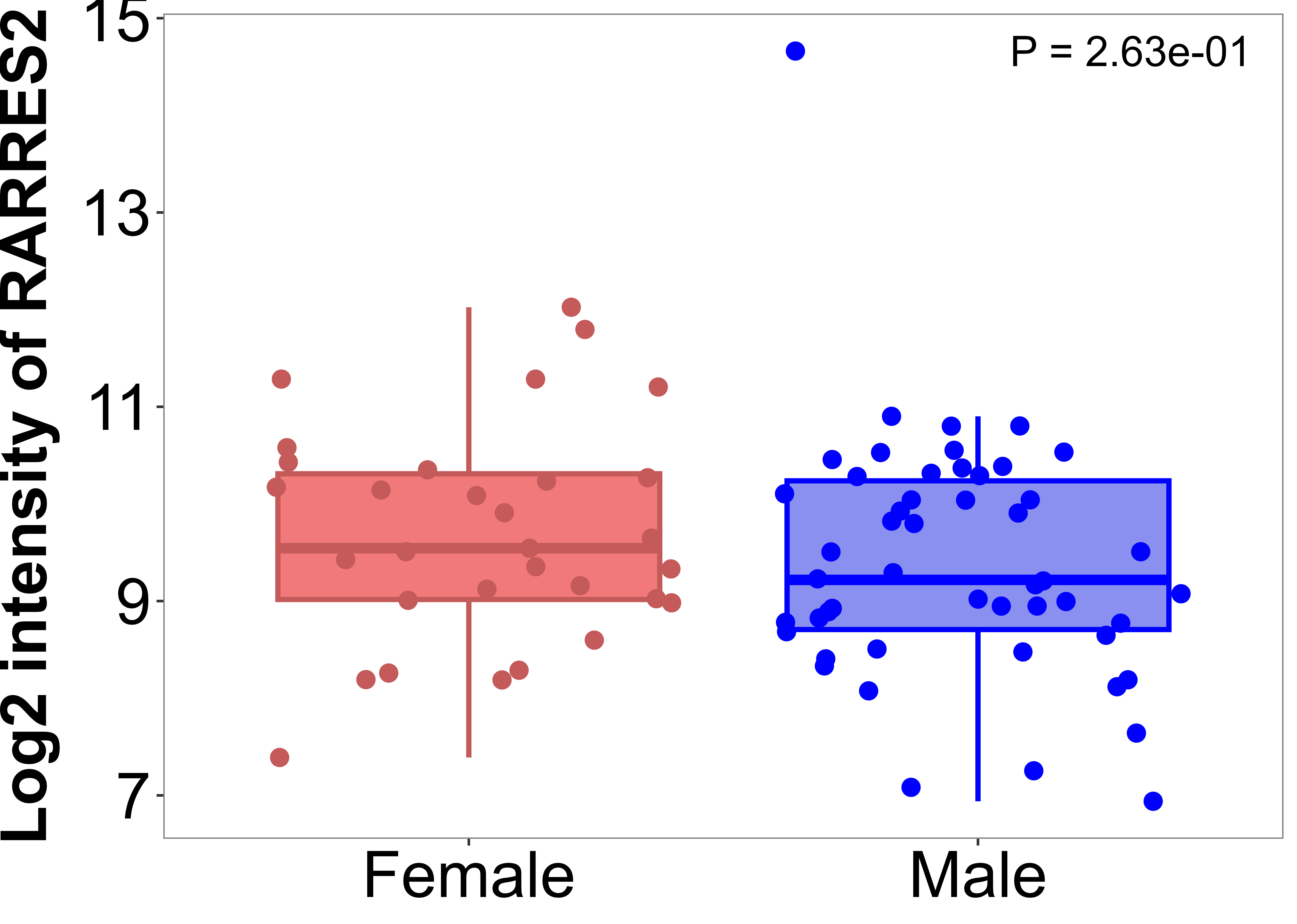

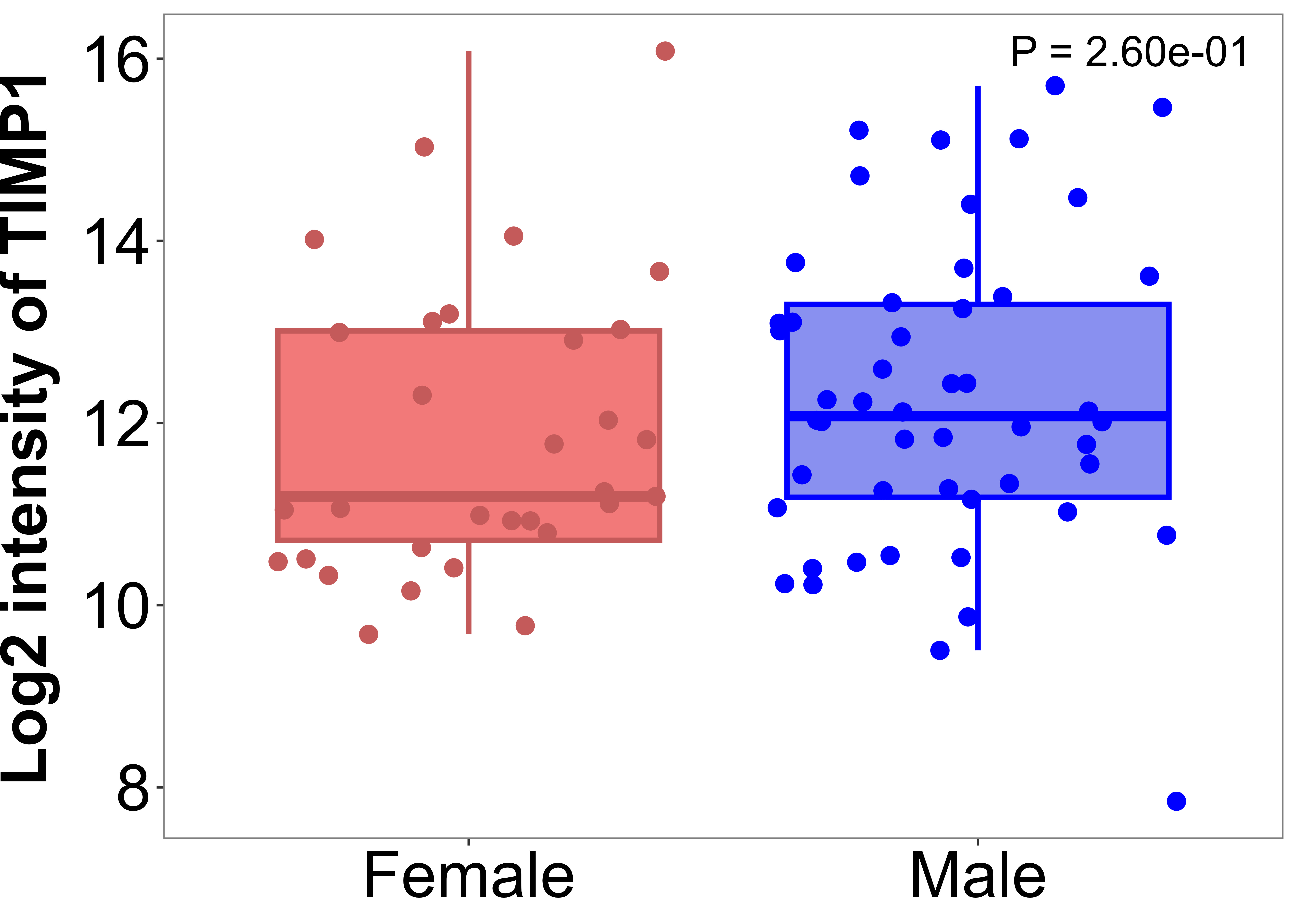


Age Plots


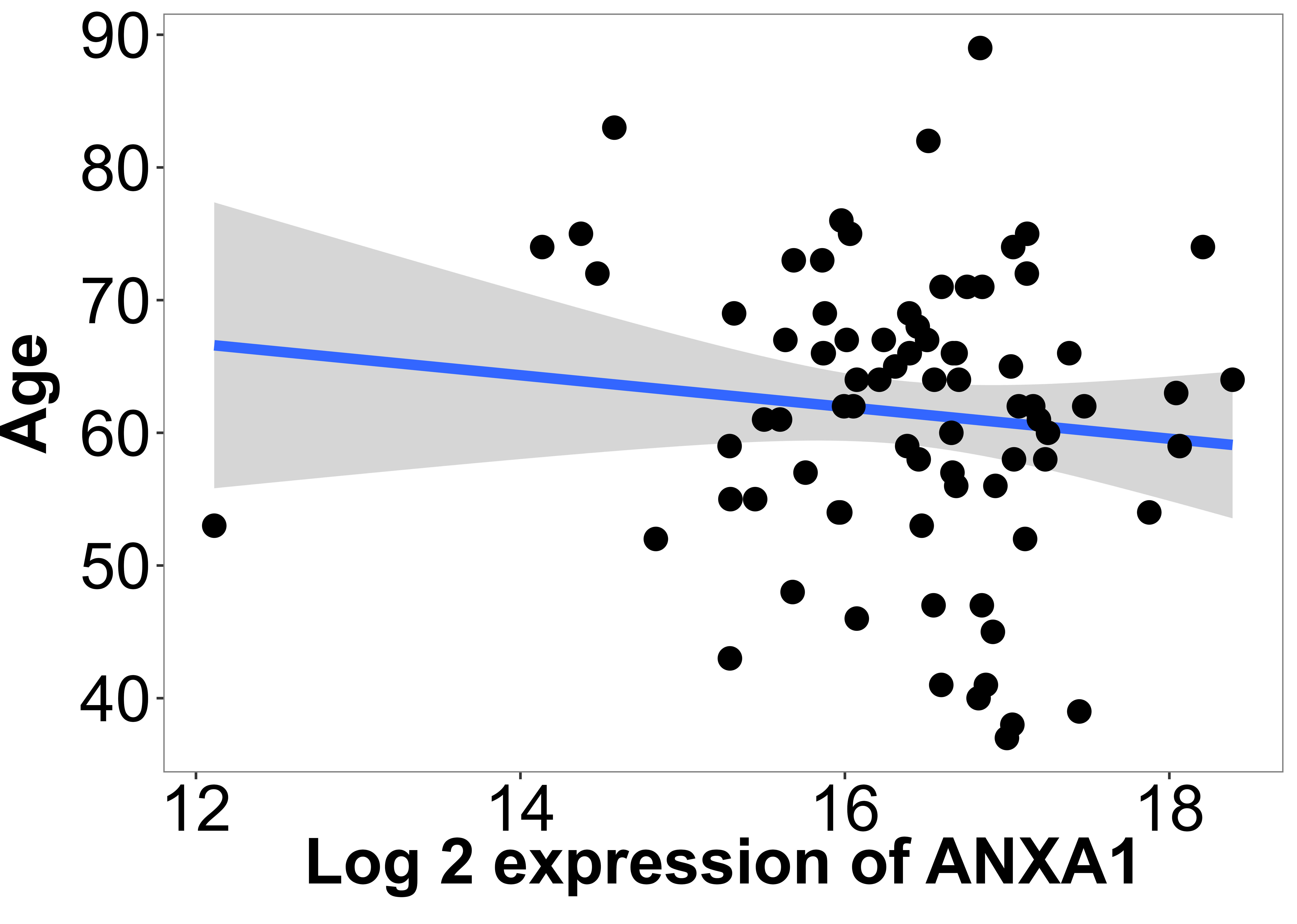

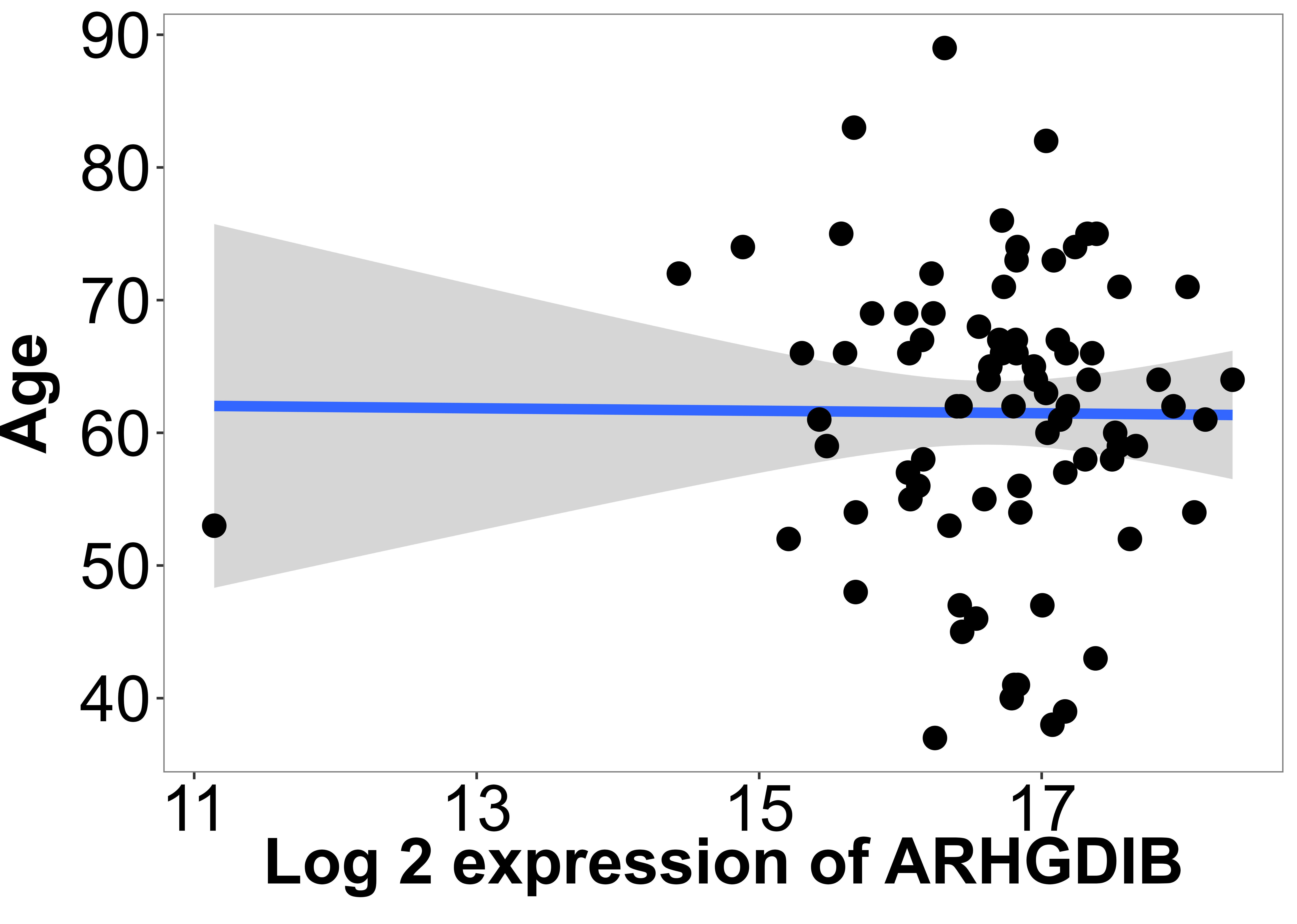

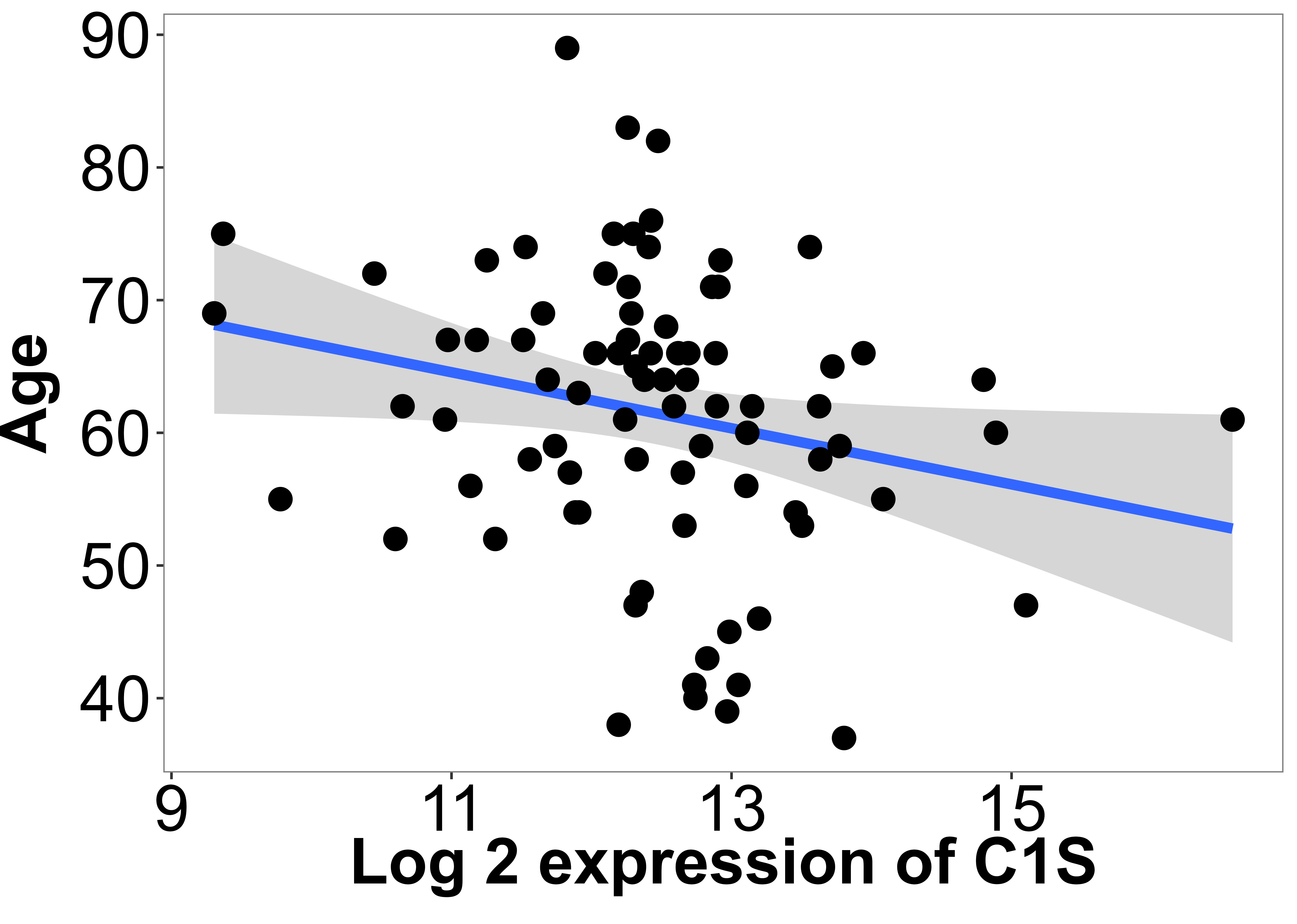

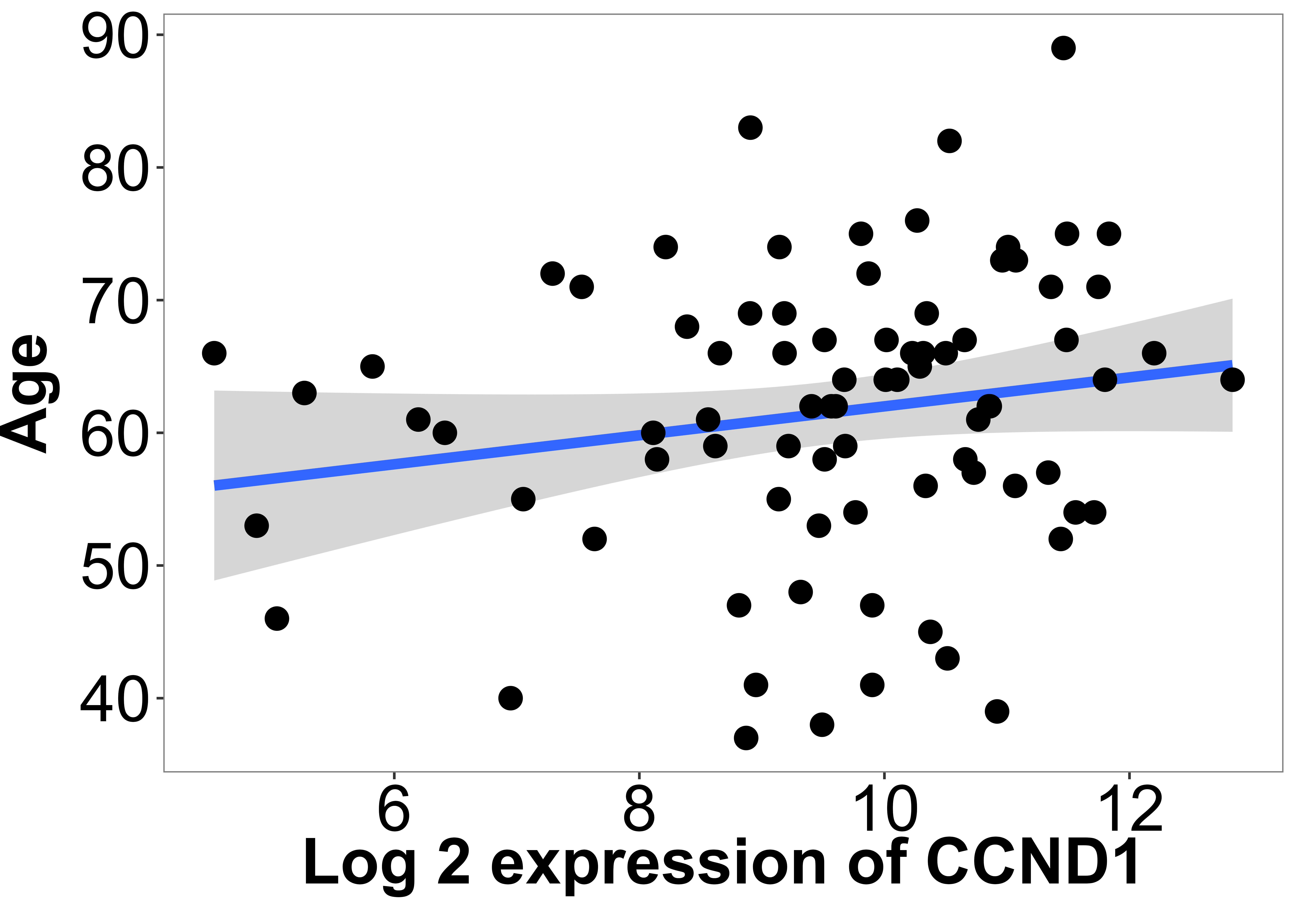

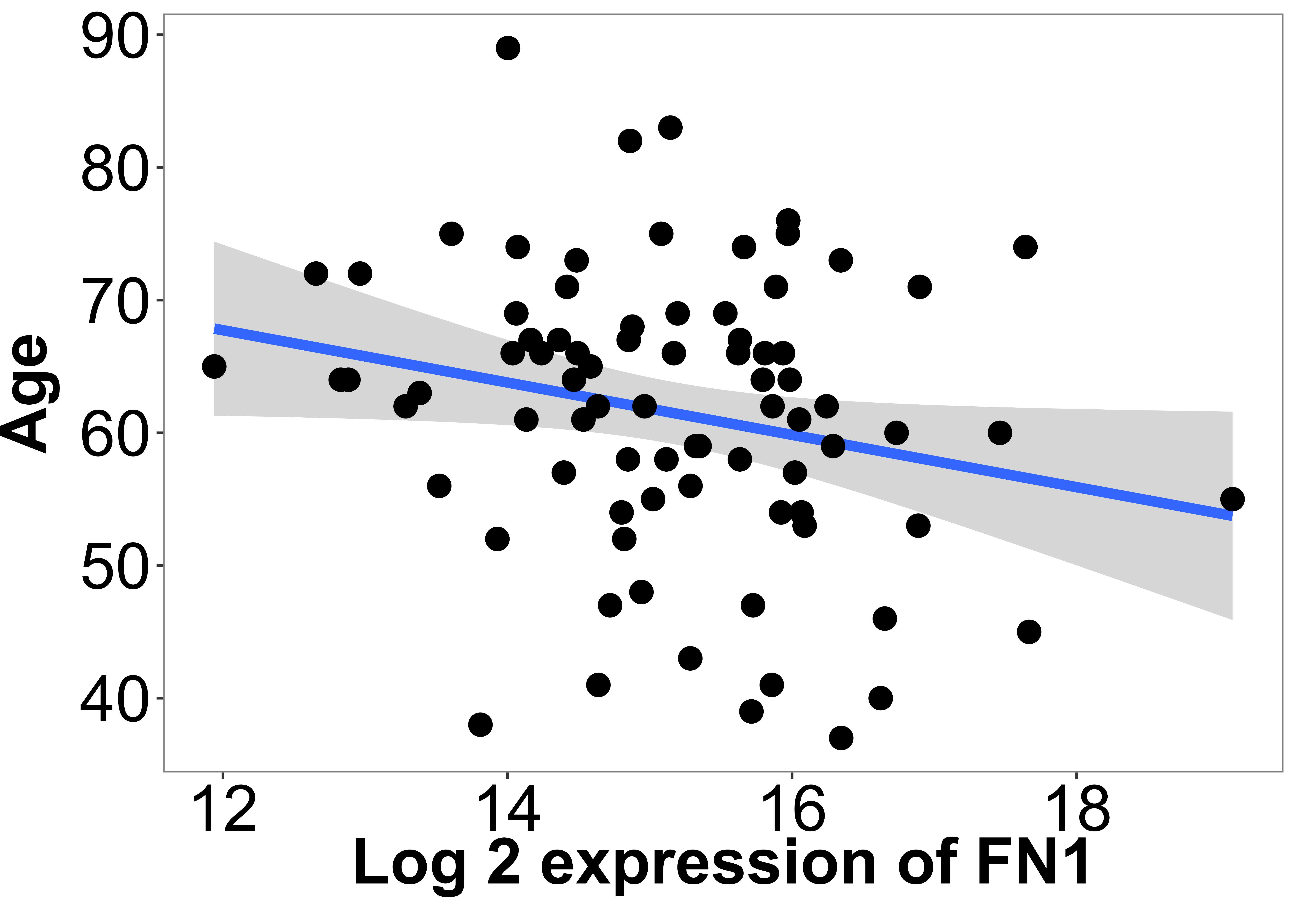

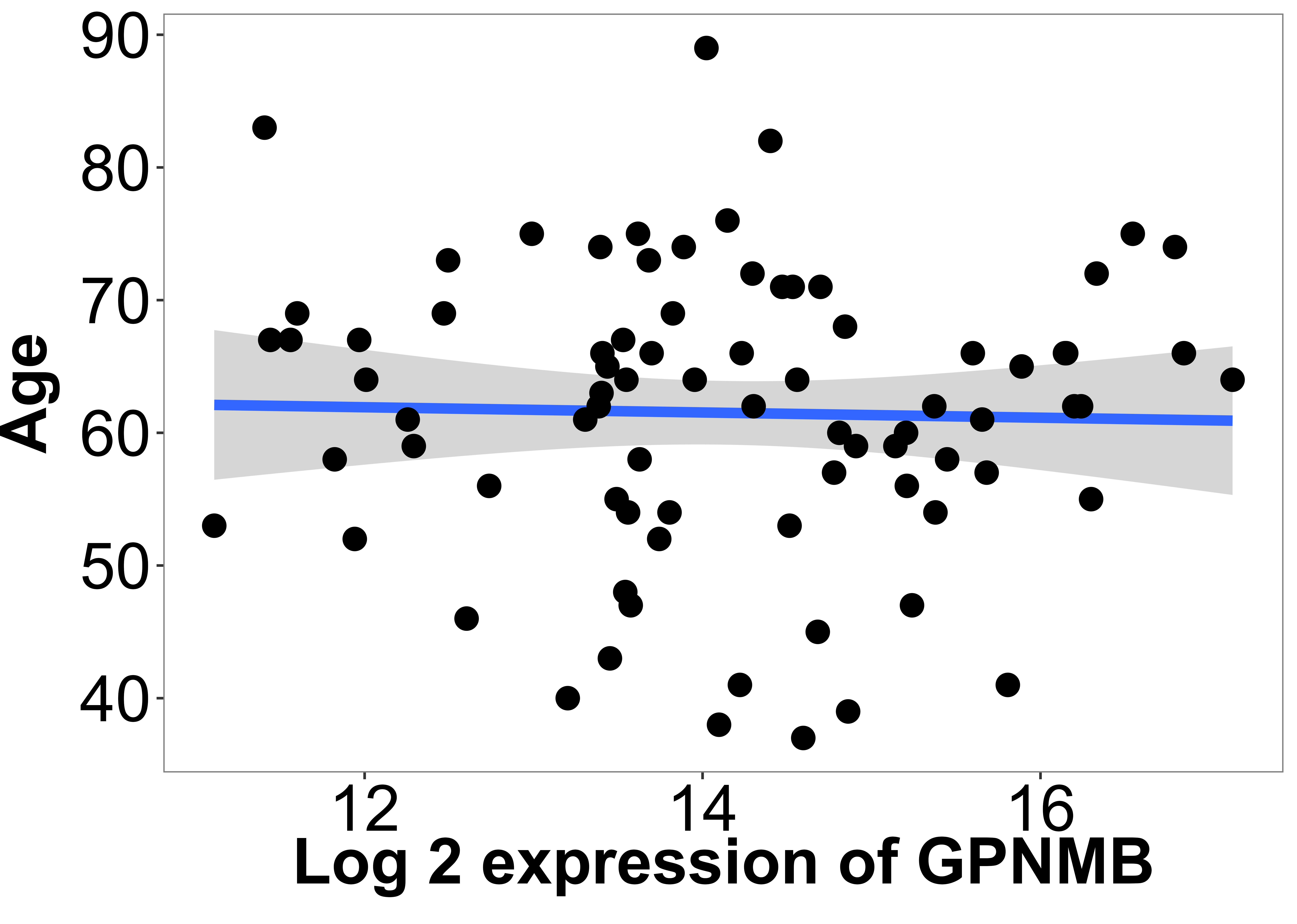

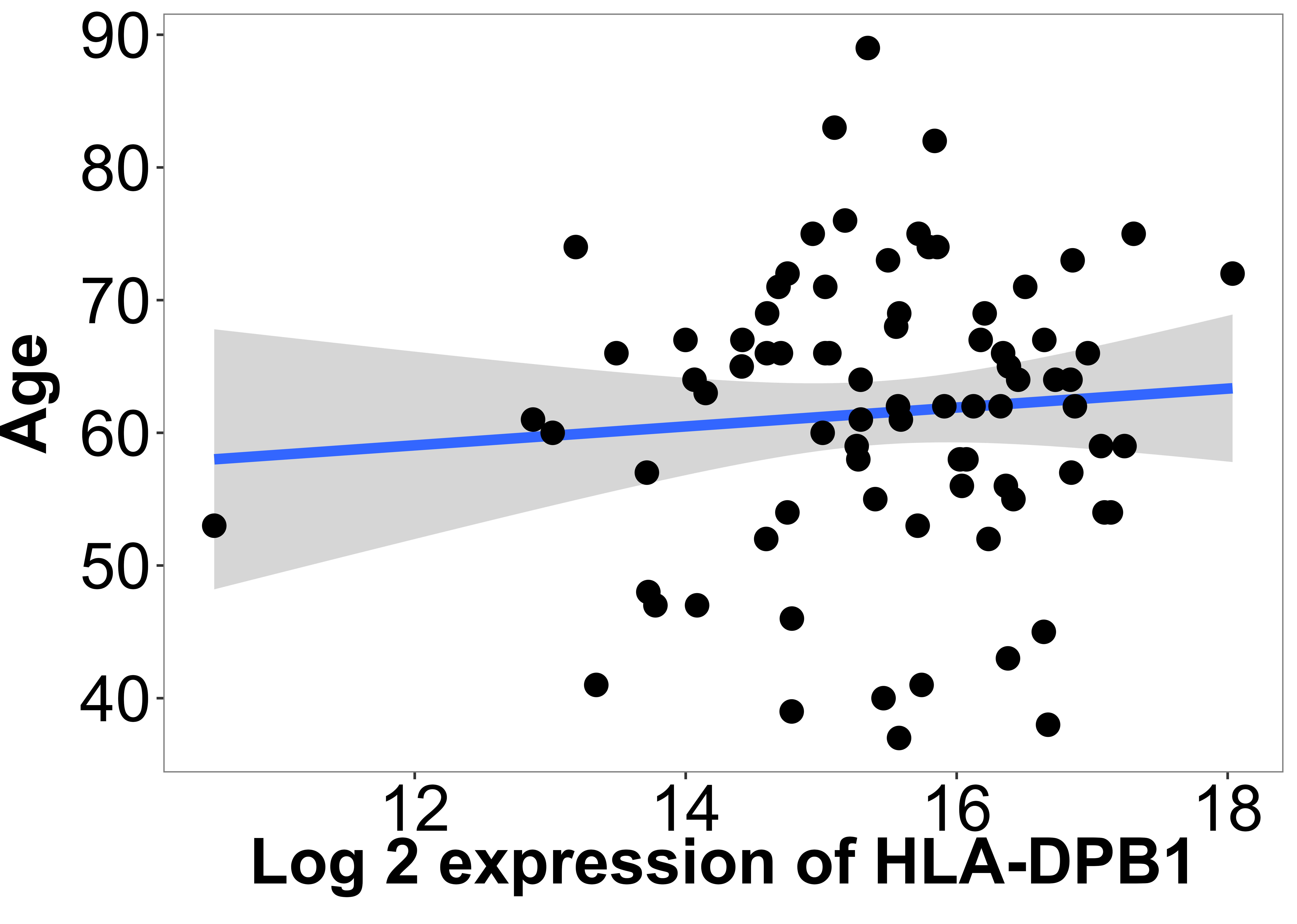

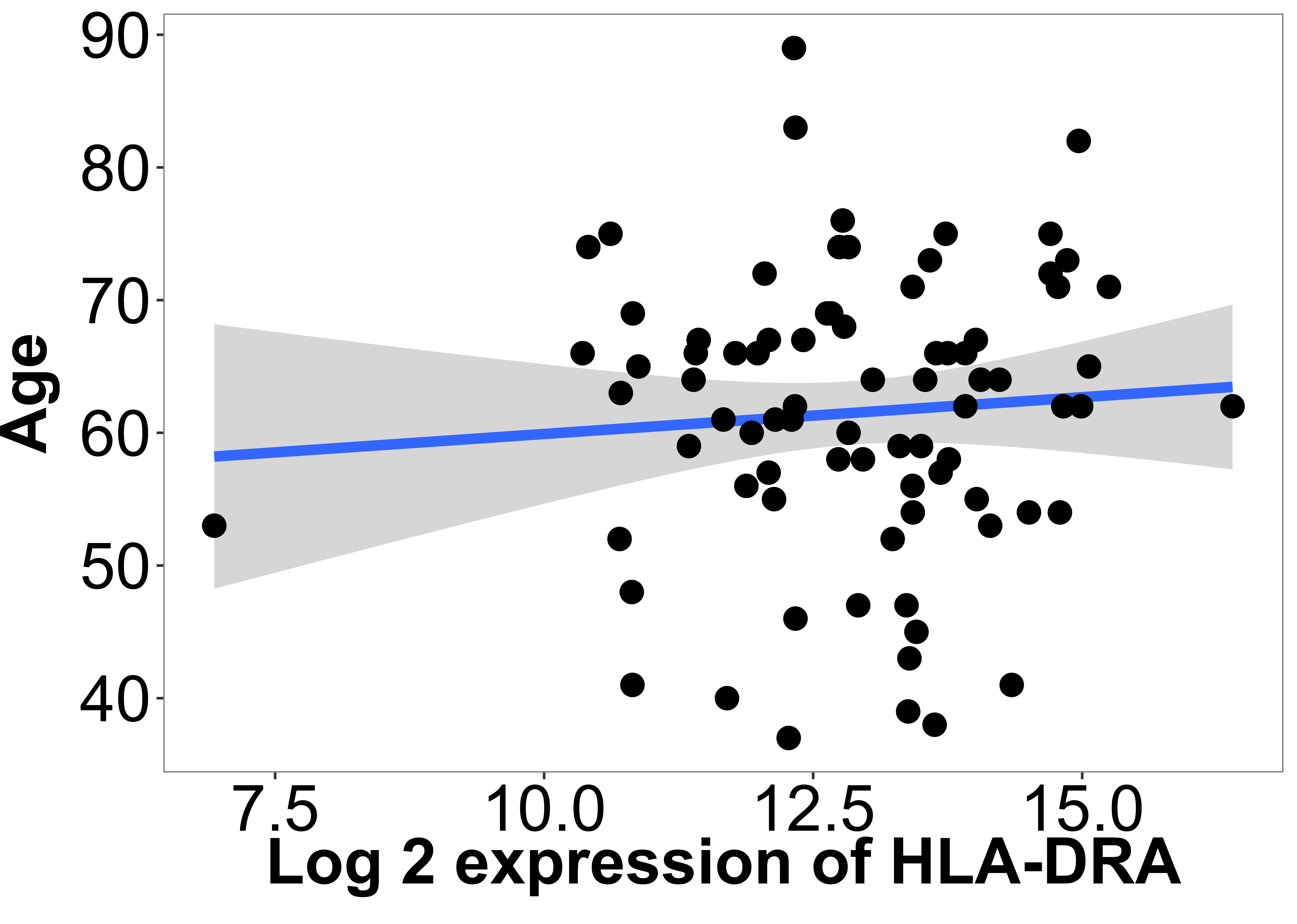

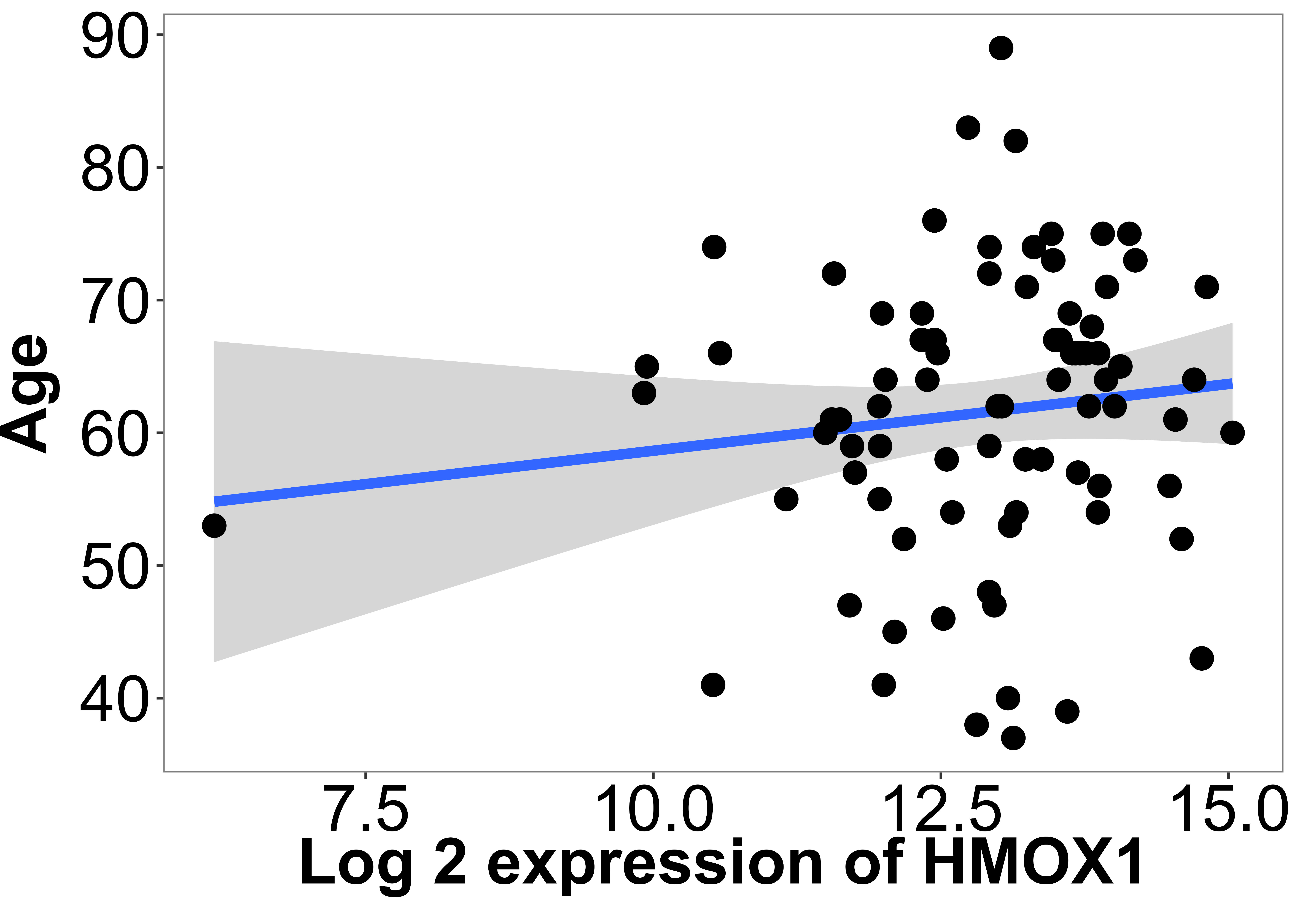

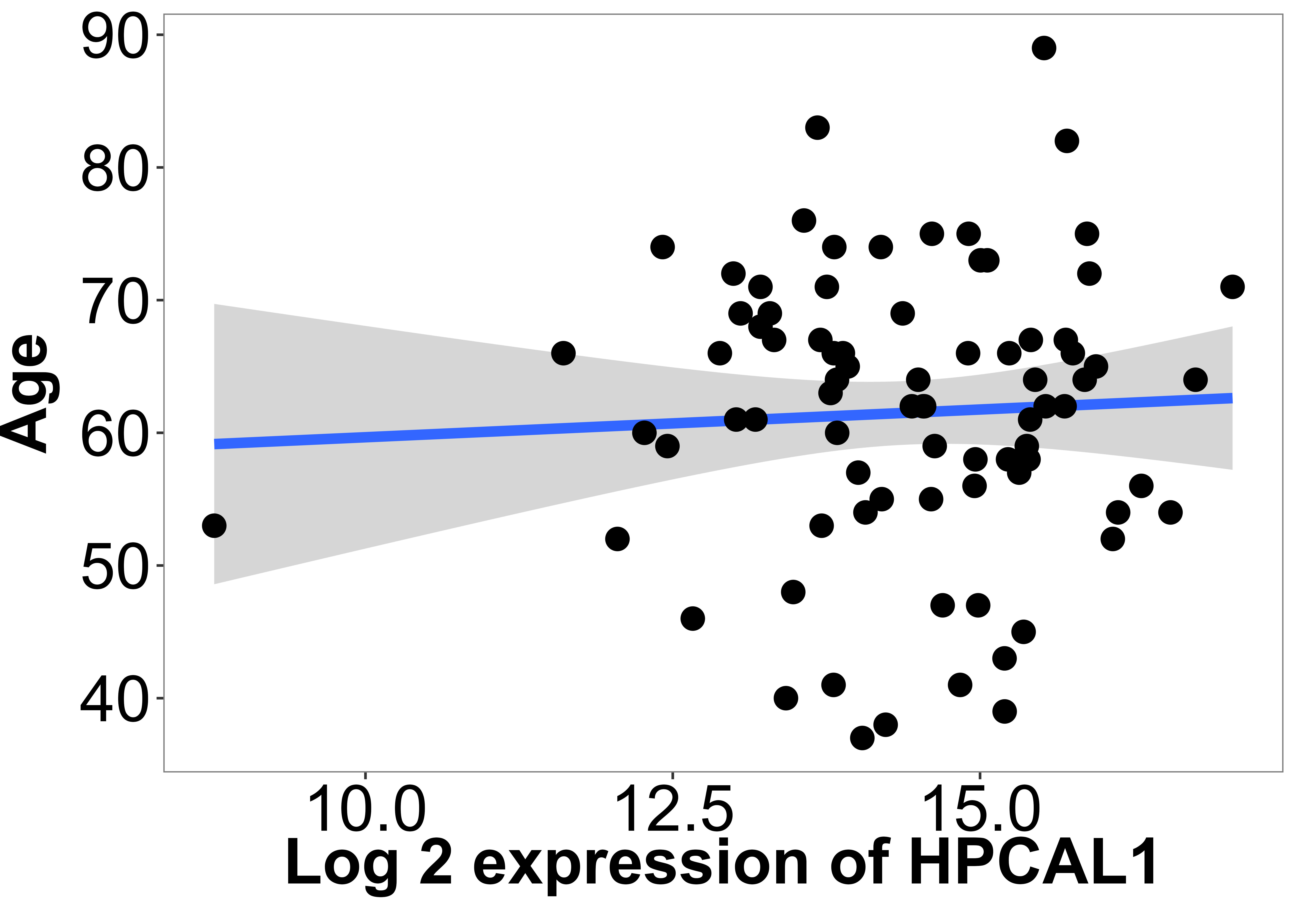

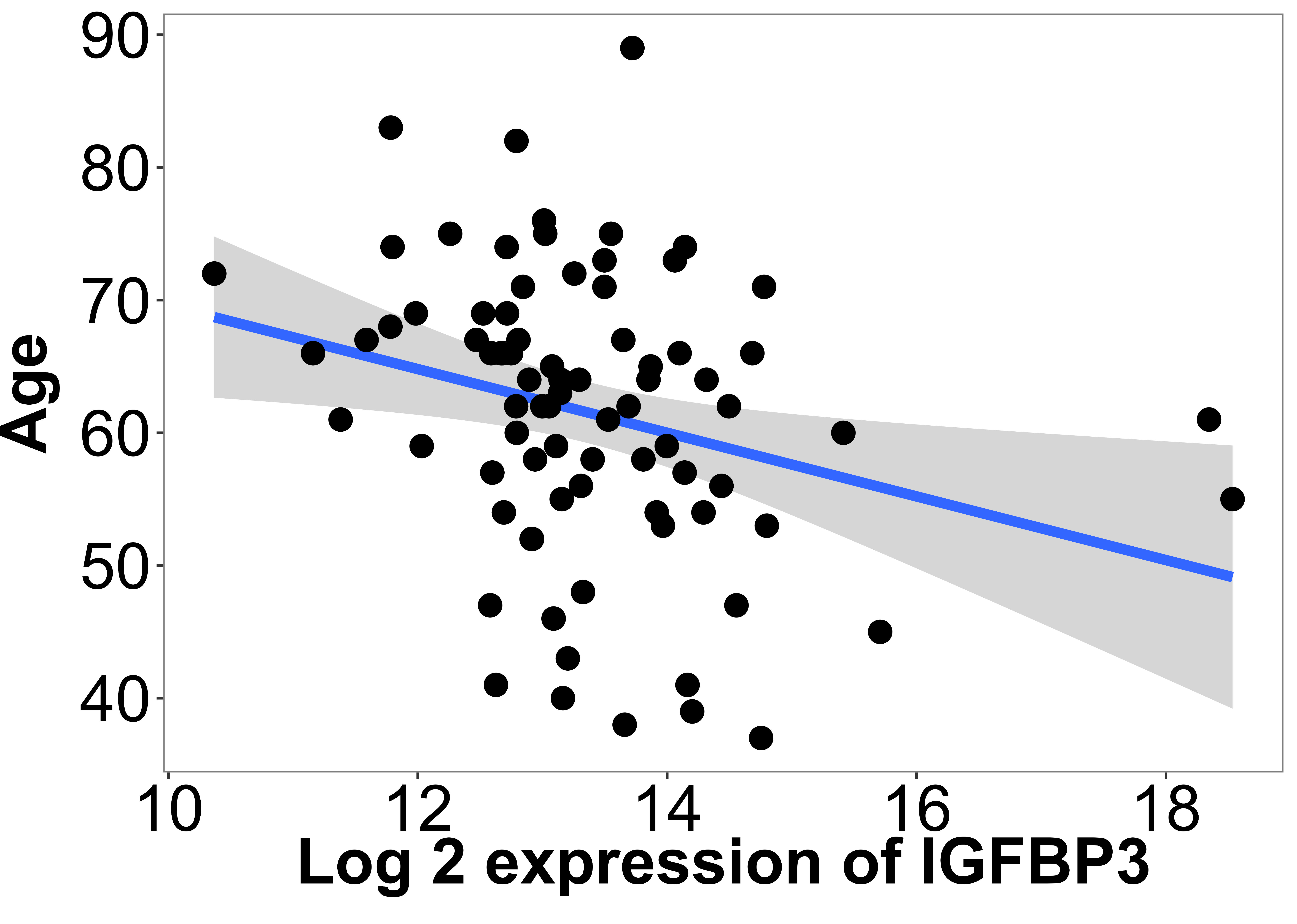

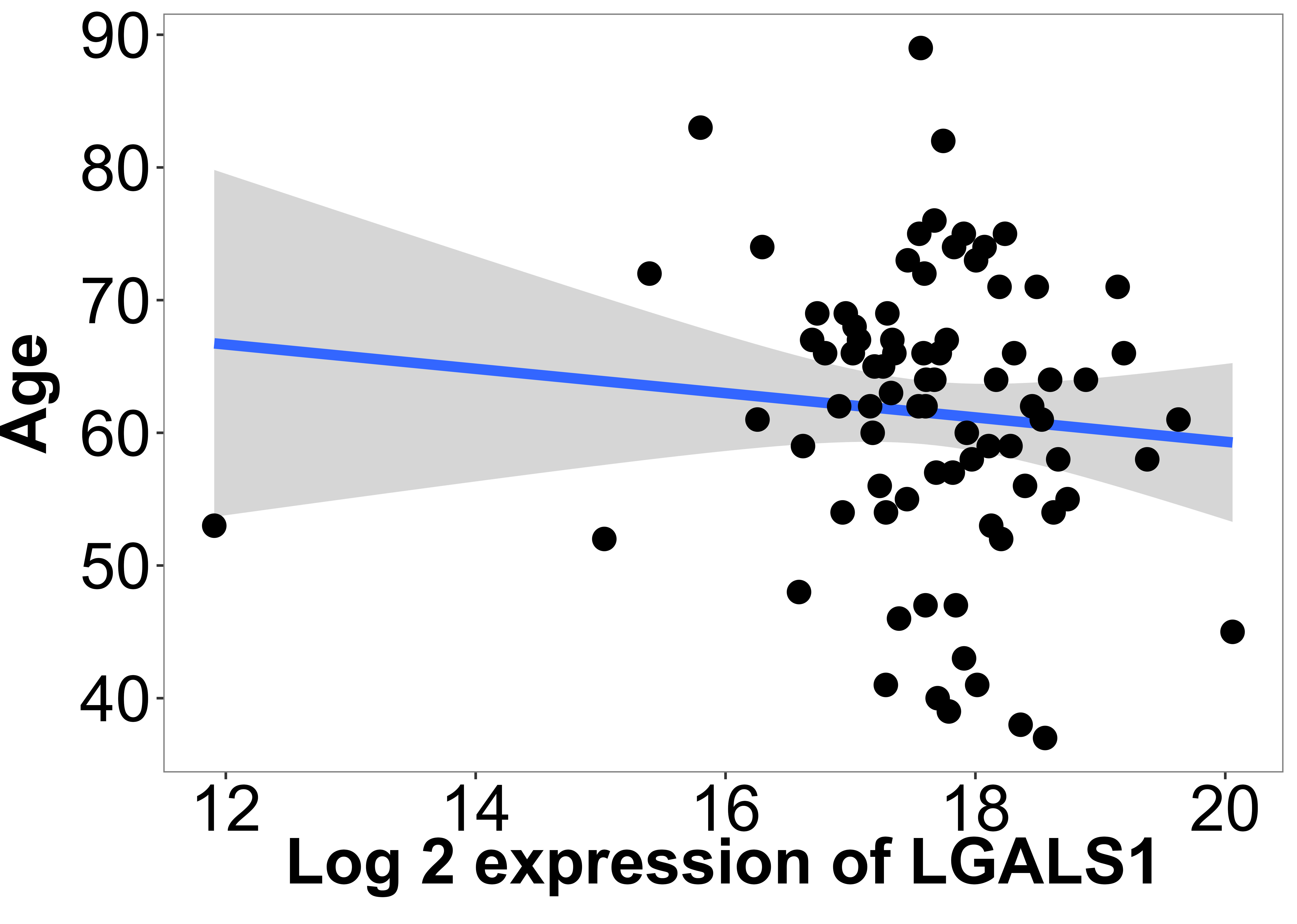

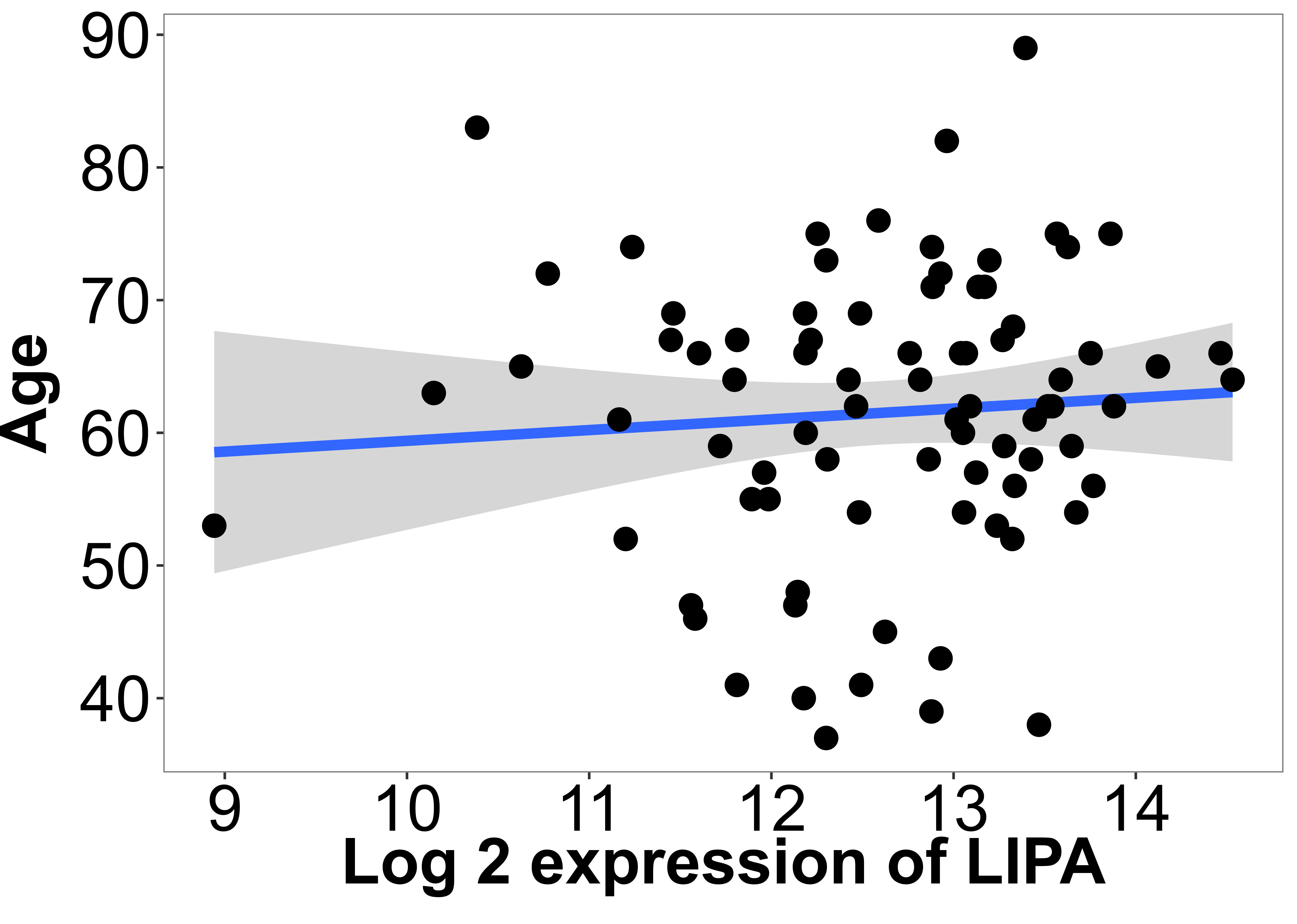

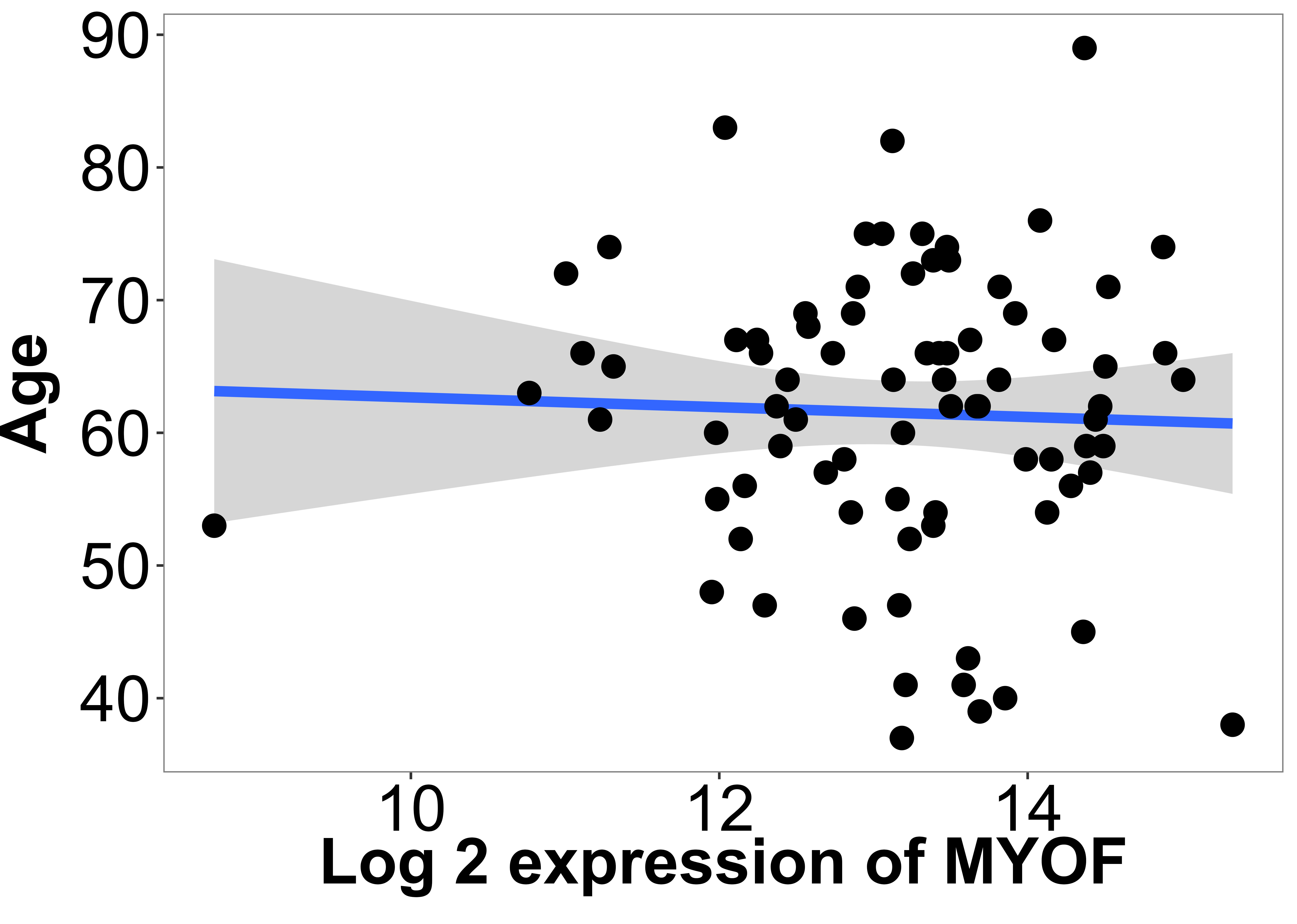

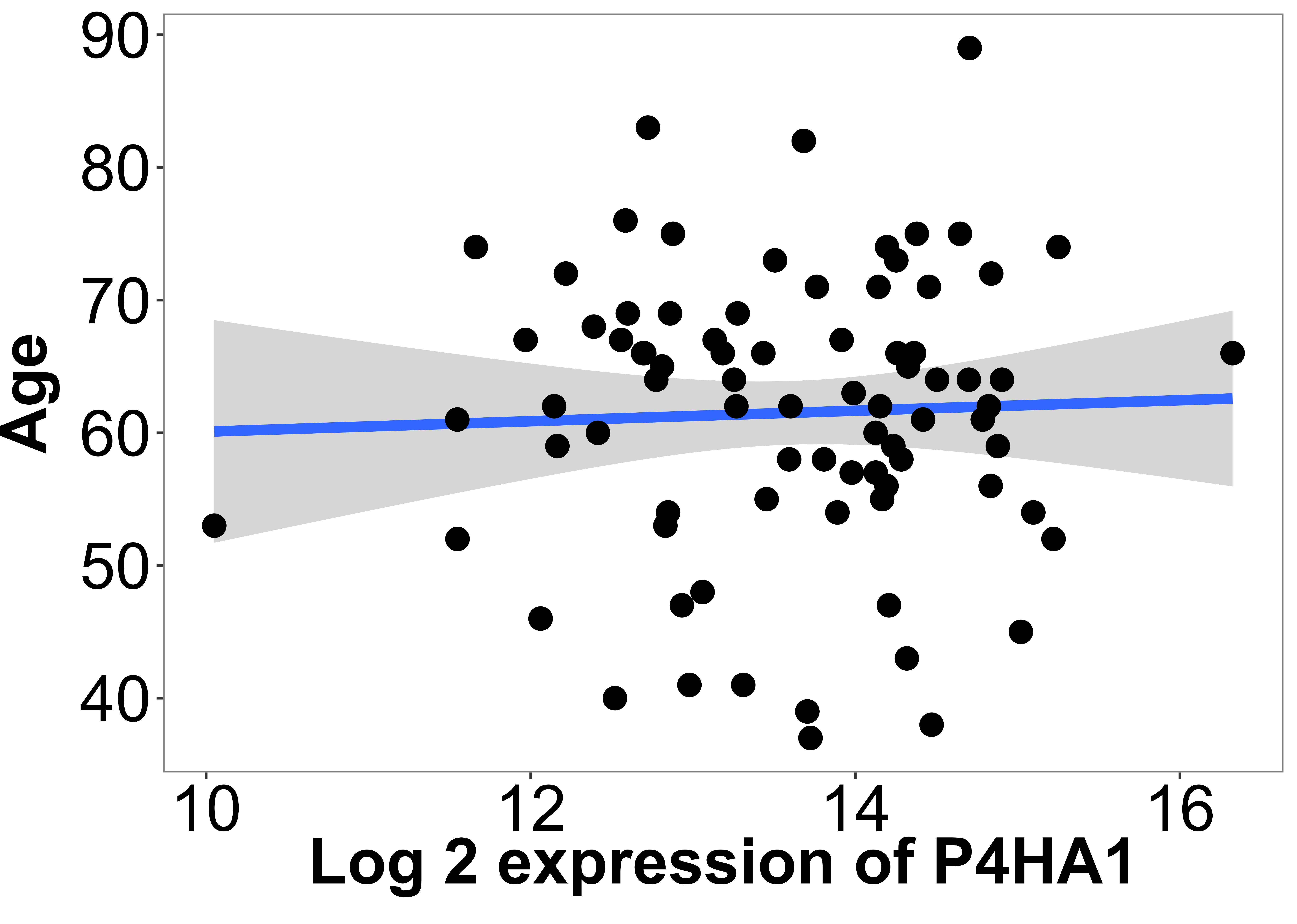

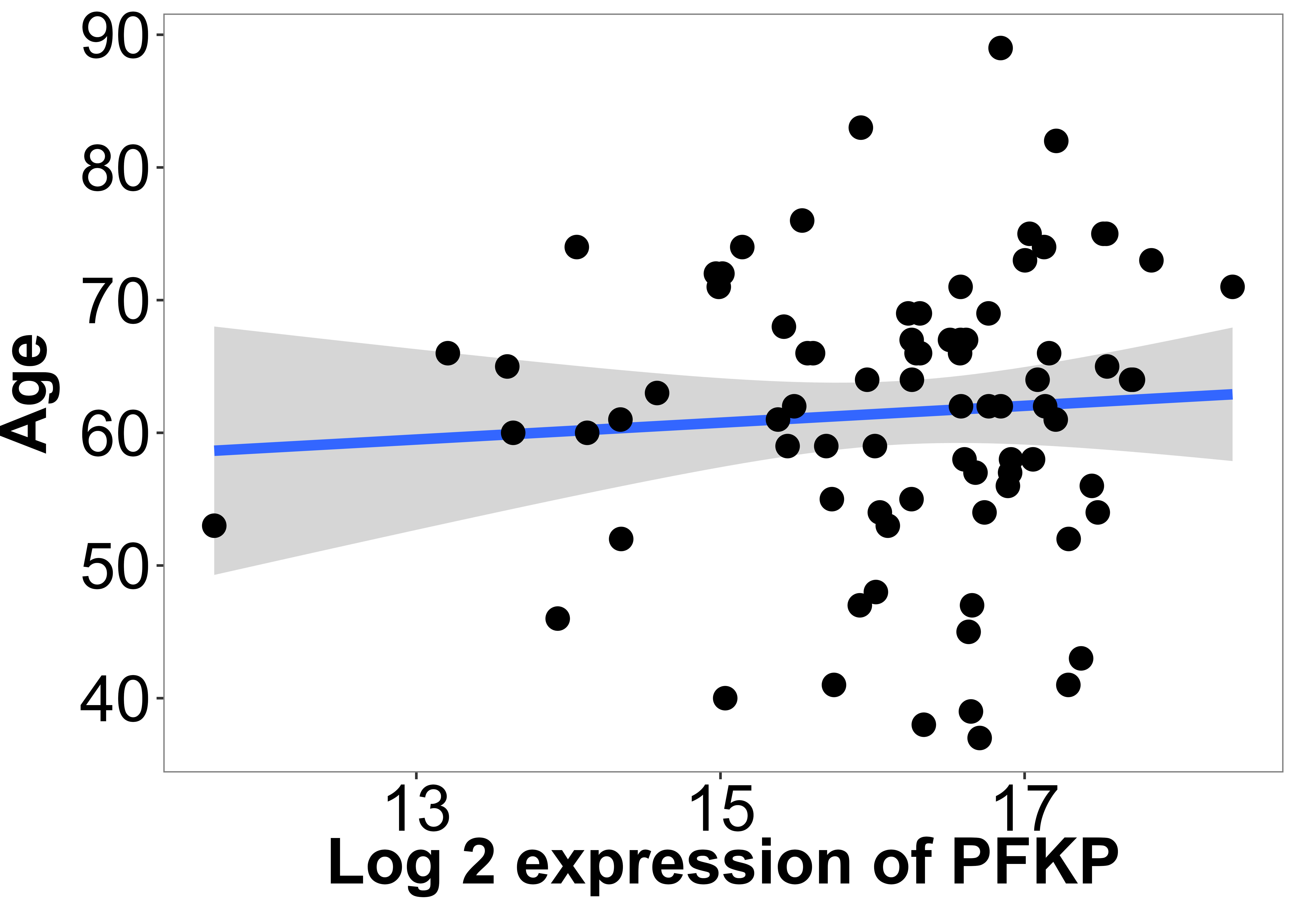

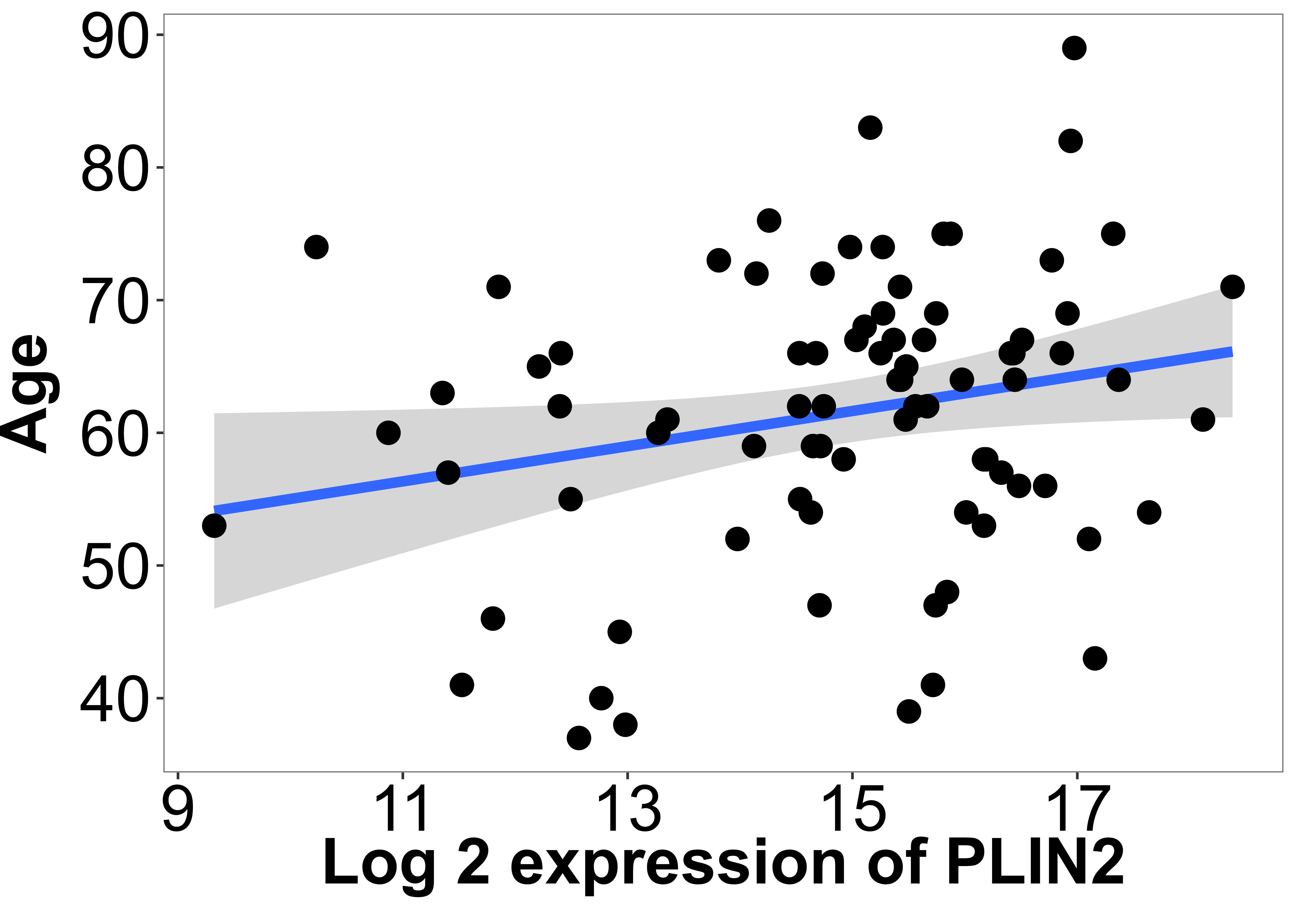

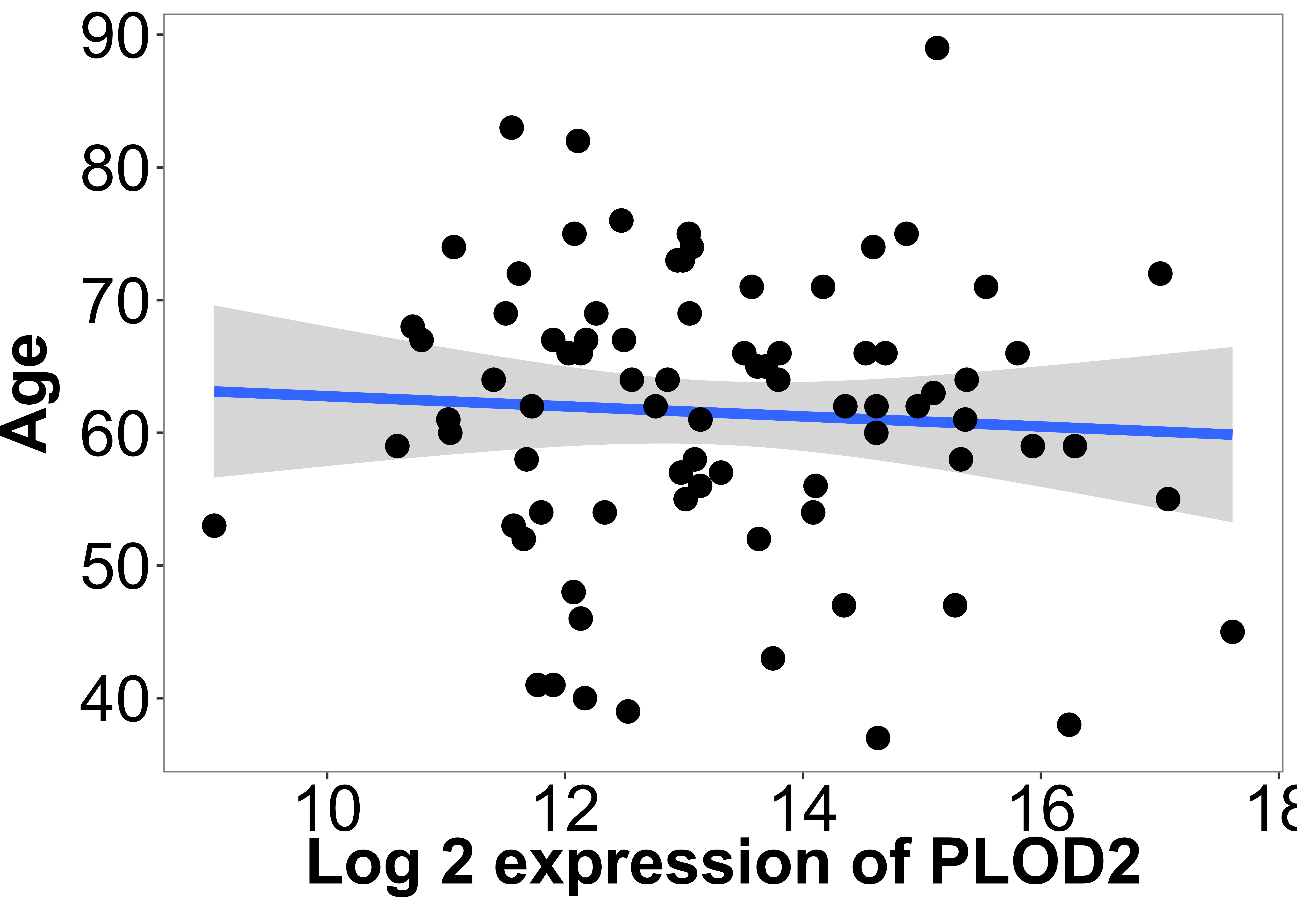

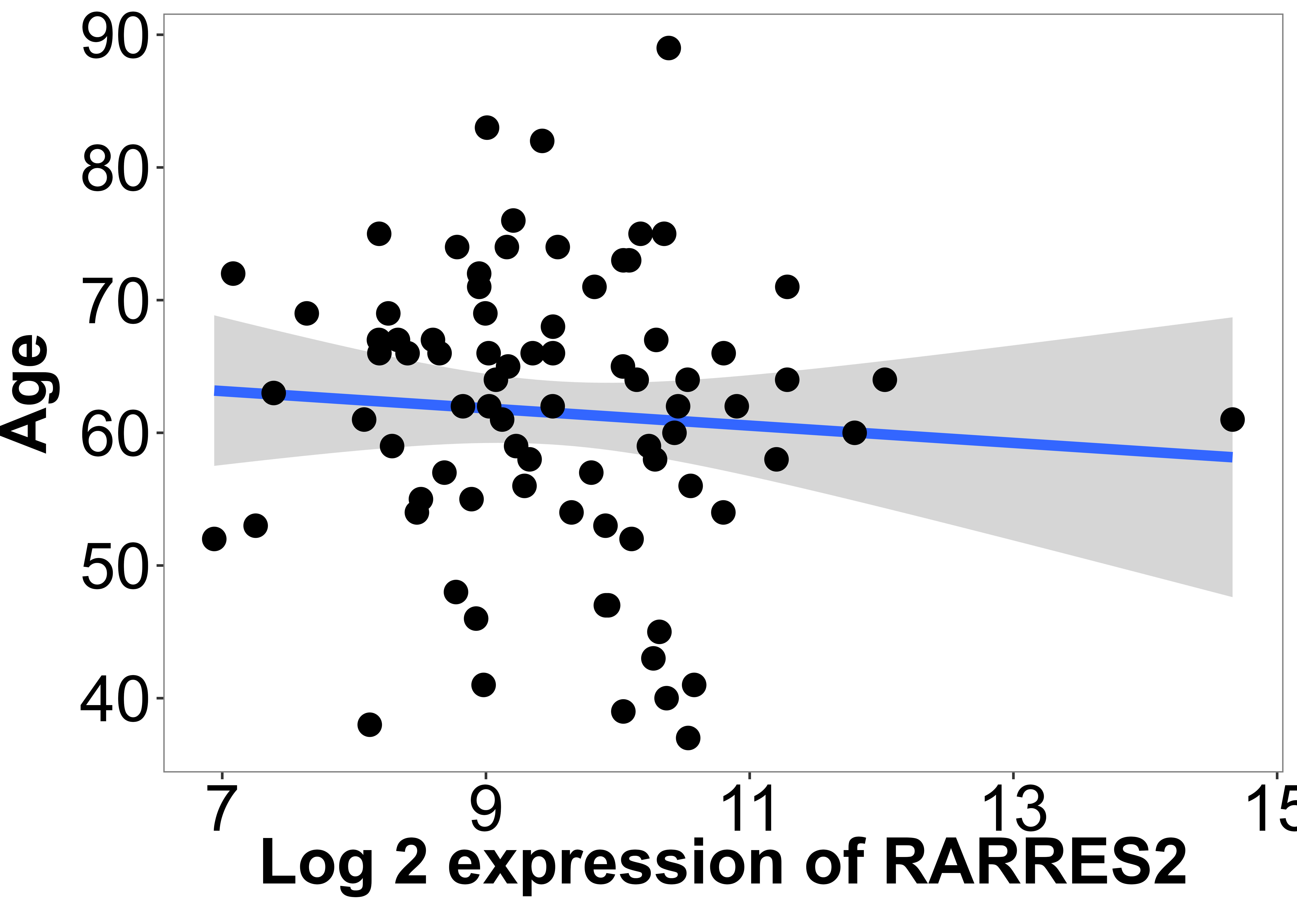

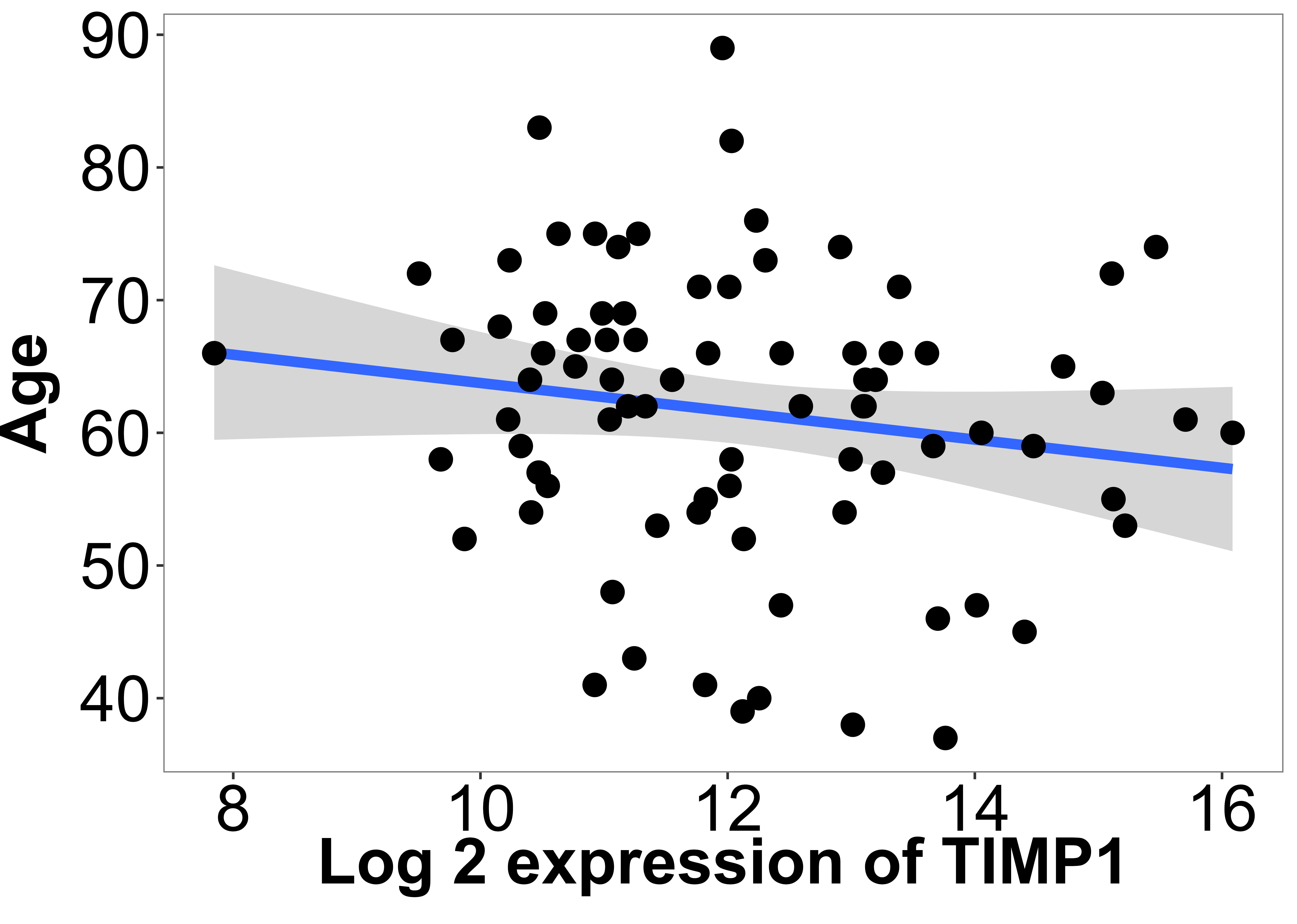

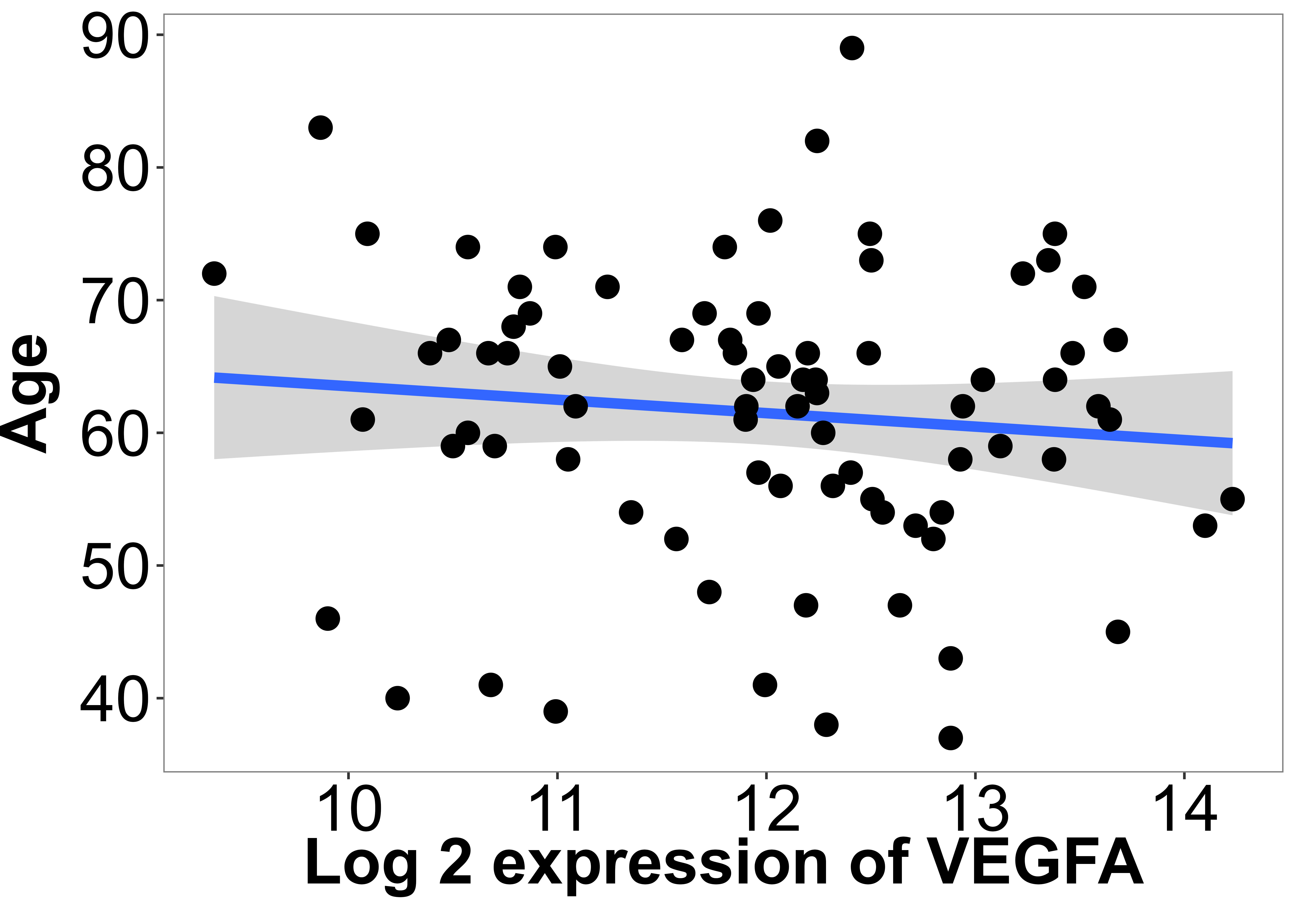

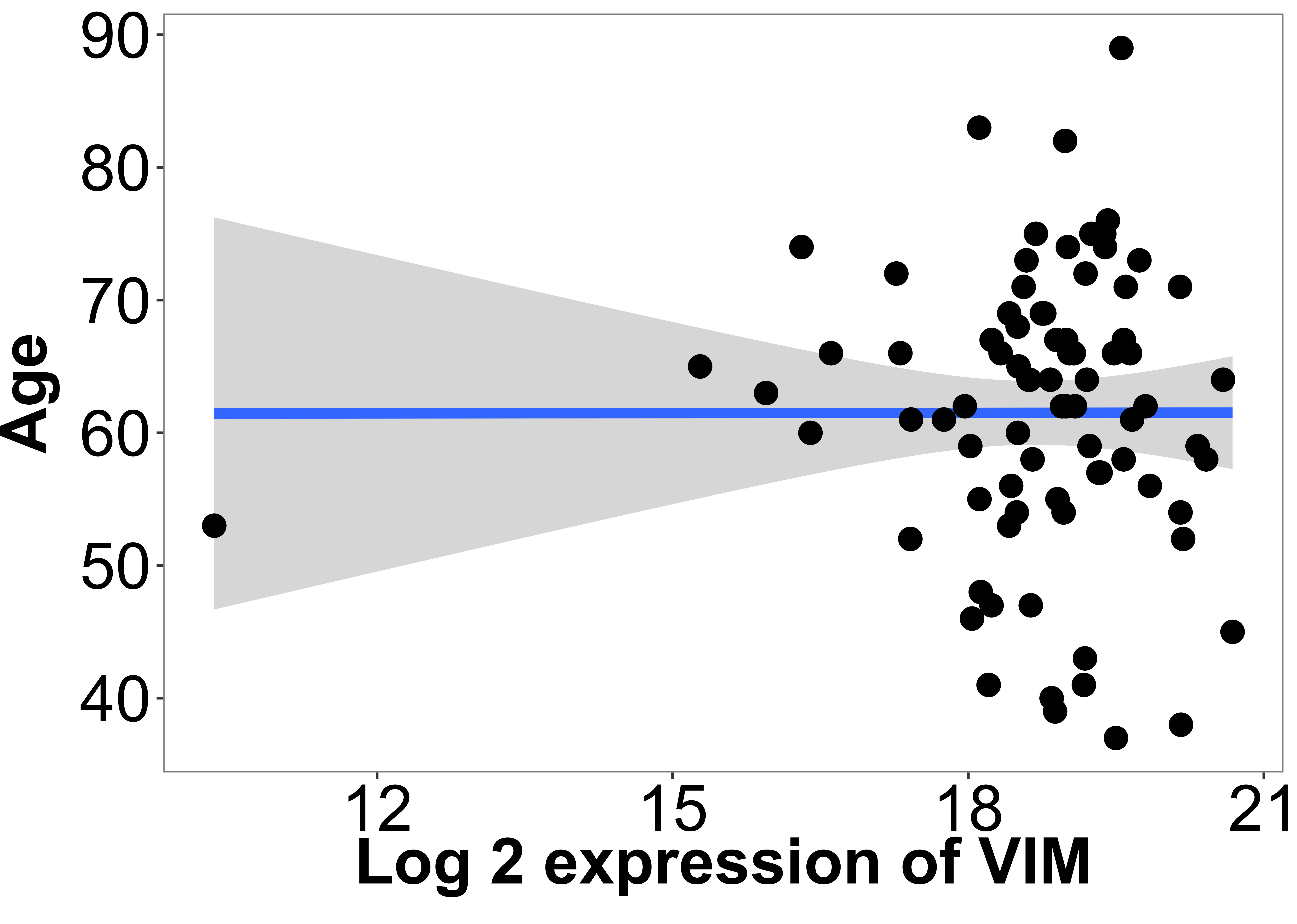

Supplement: Supplementary file 1 [file ijms-24-04488-s001.zip › covariateAnalysis.docx]

kmPlots


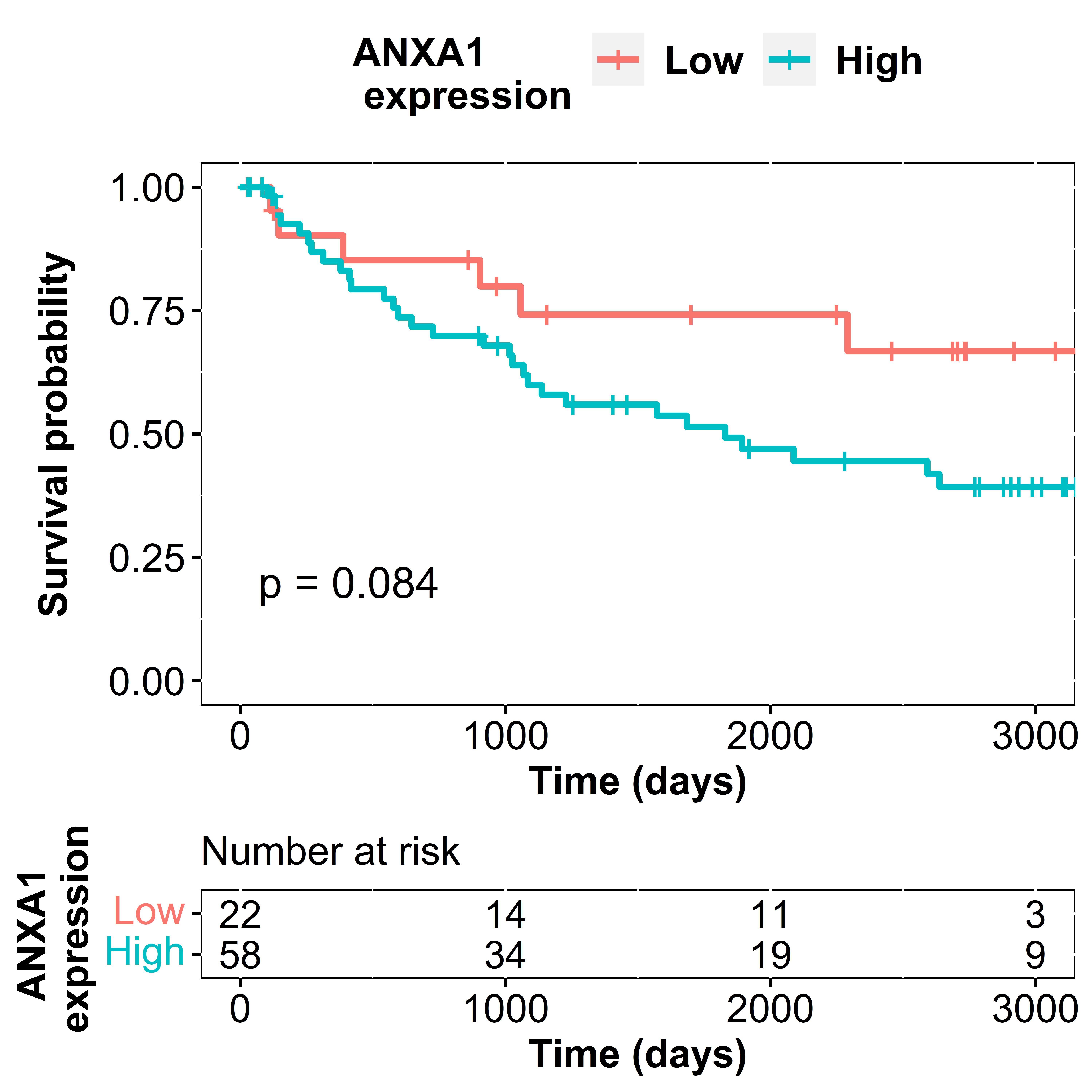

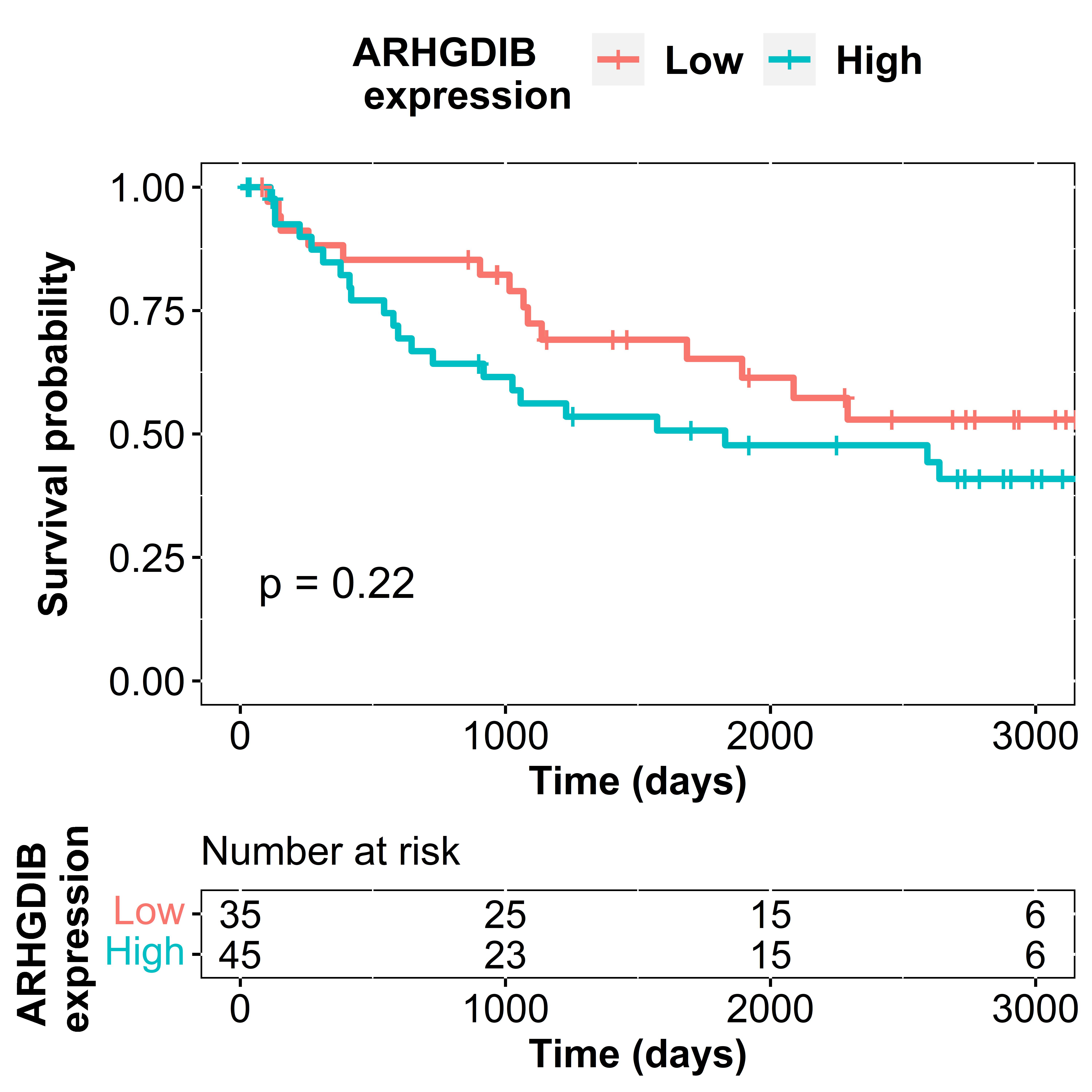

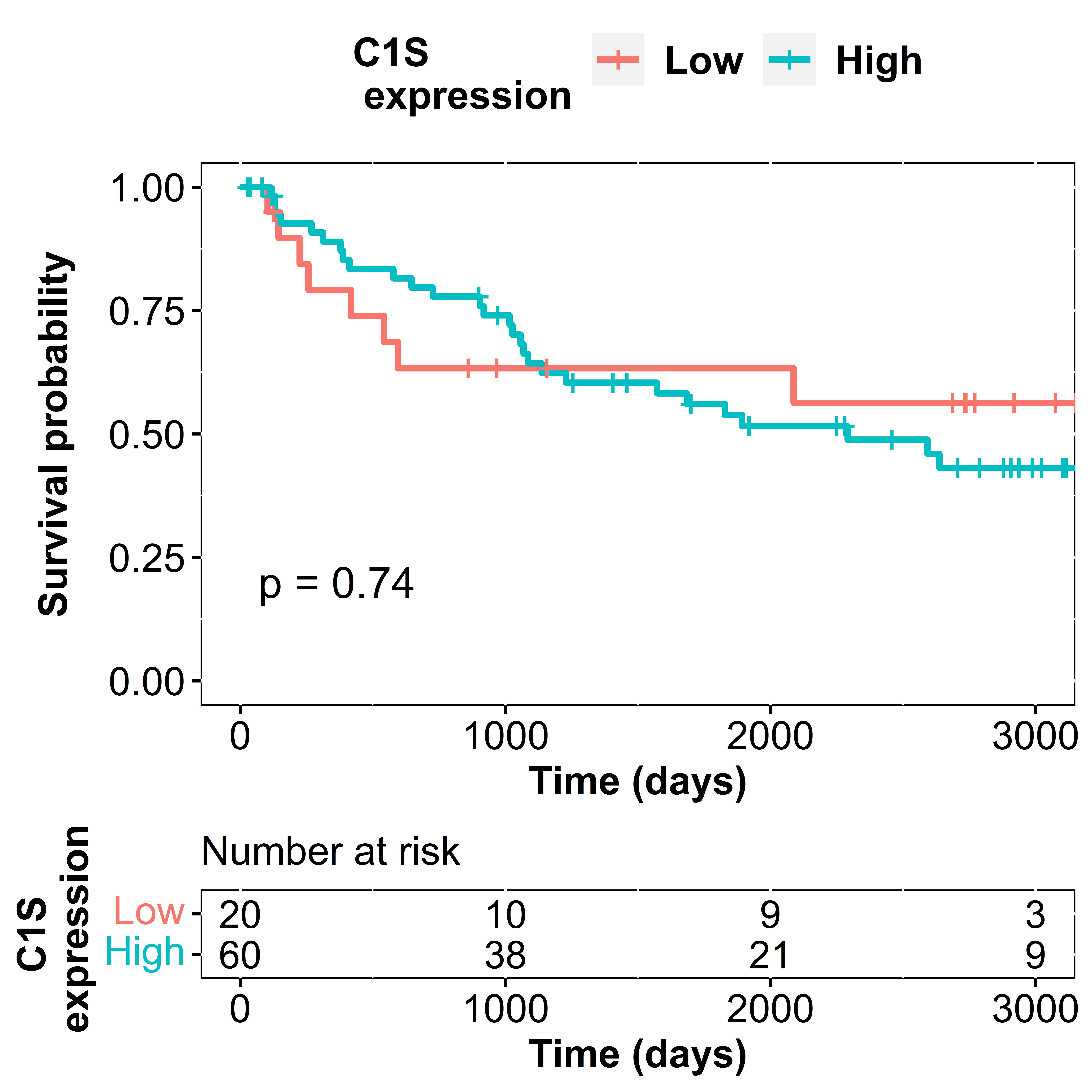

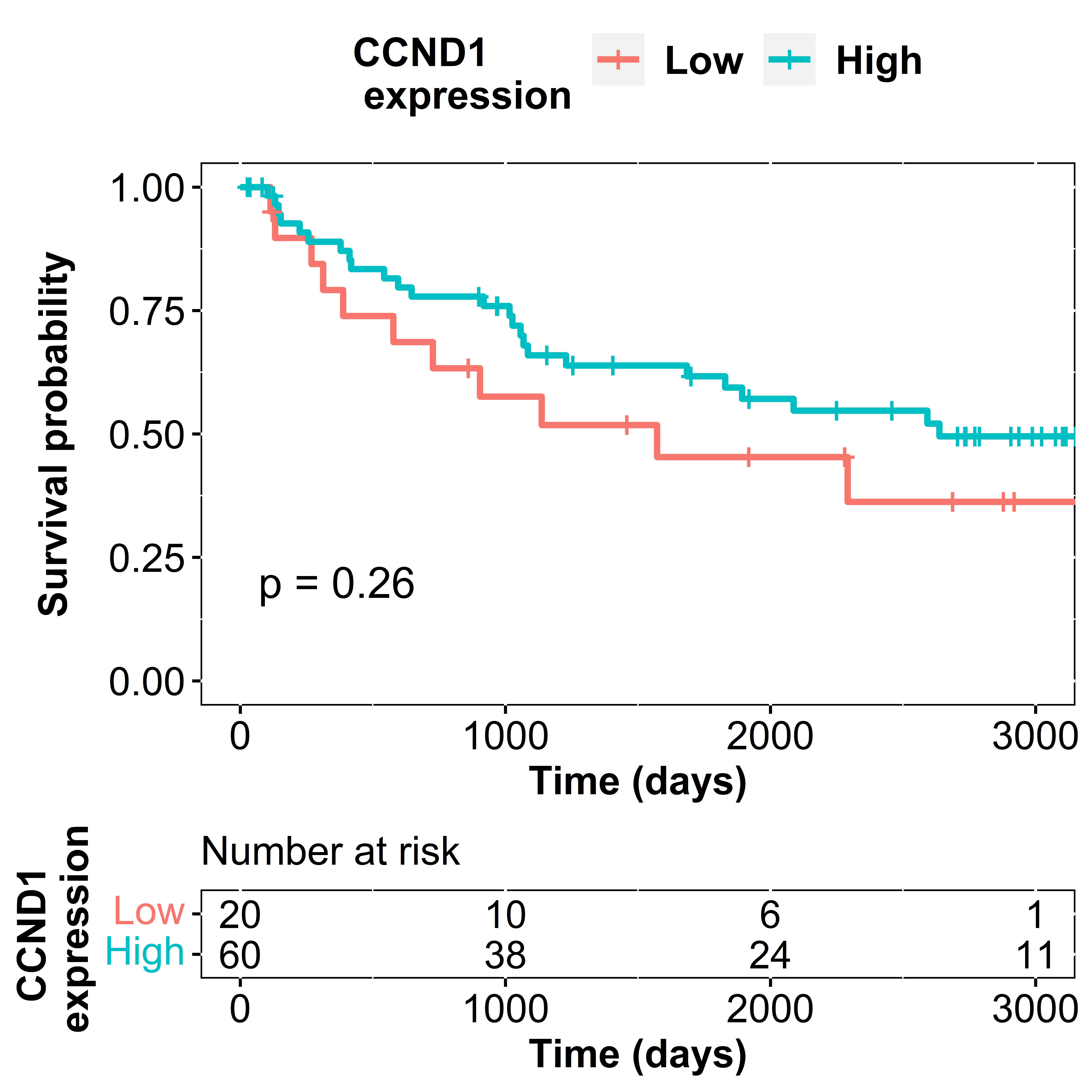

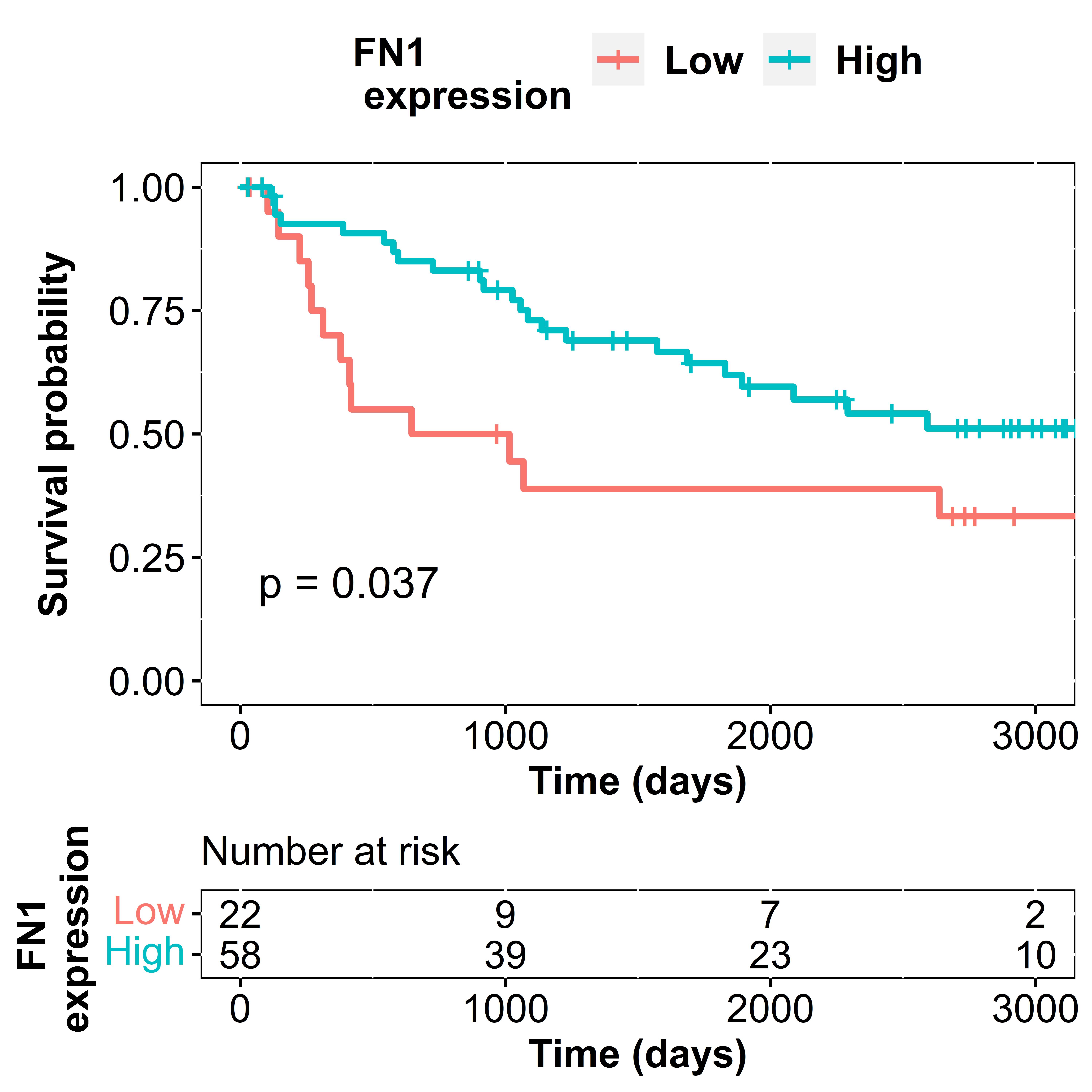

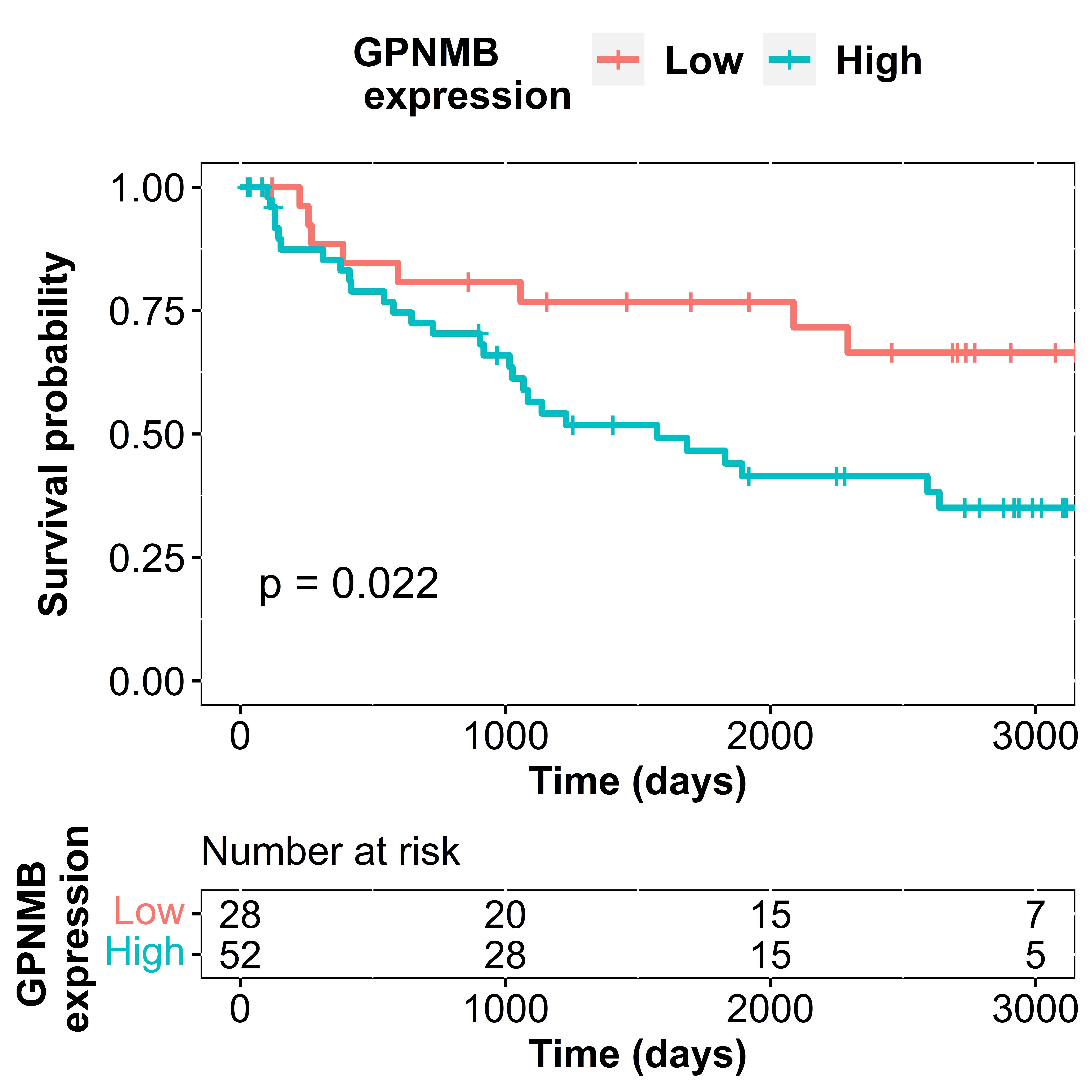

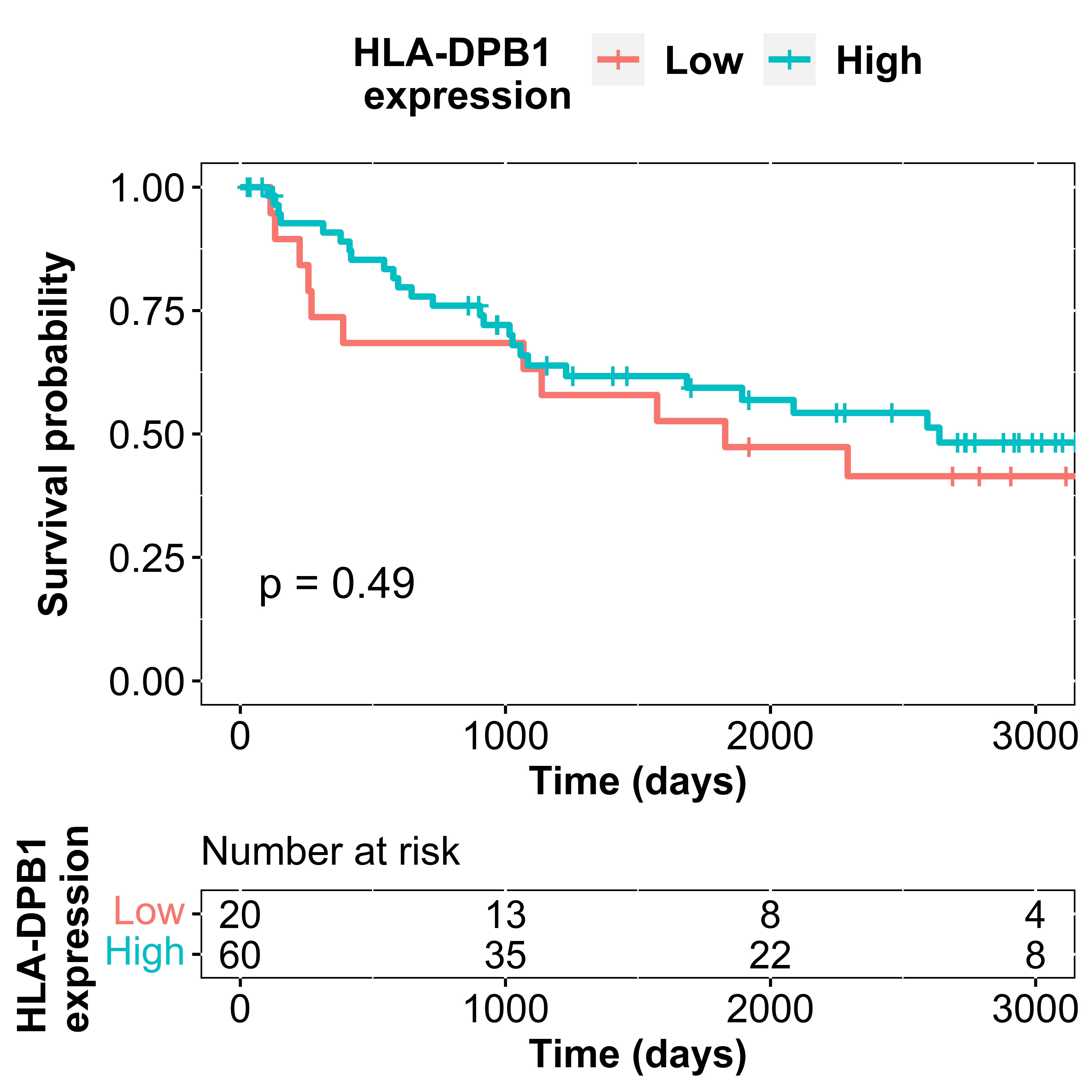

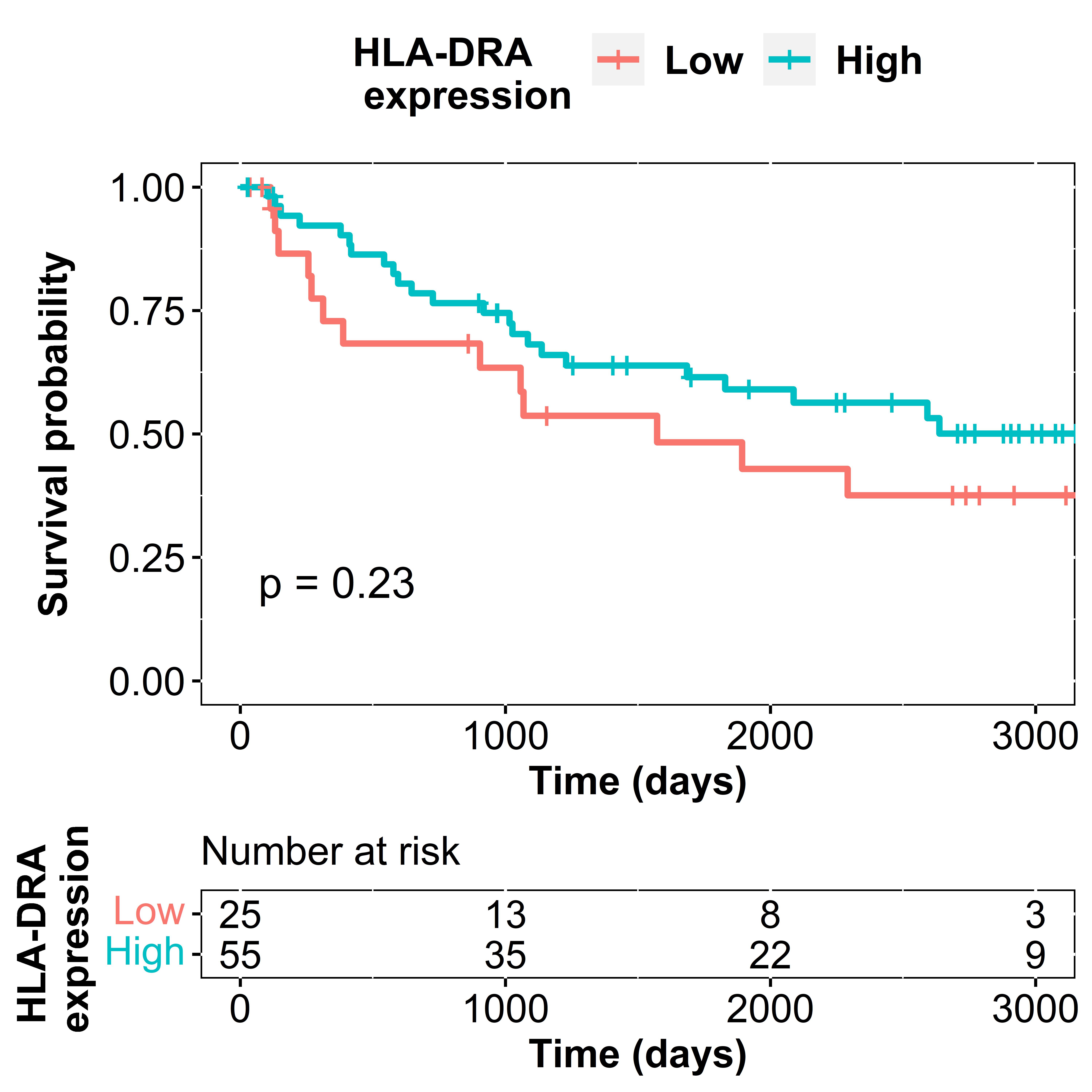

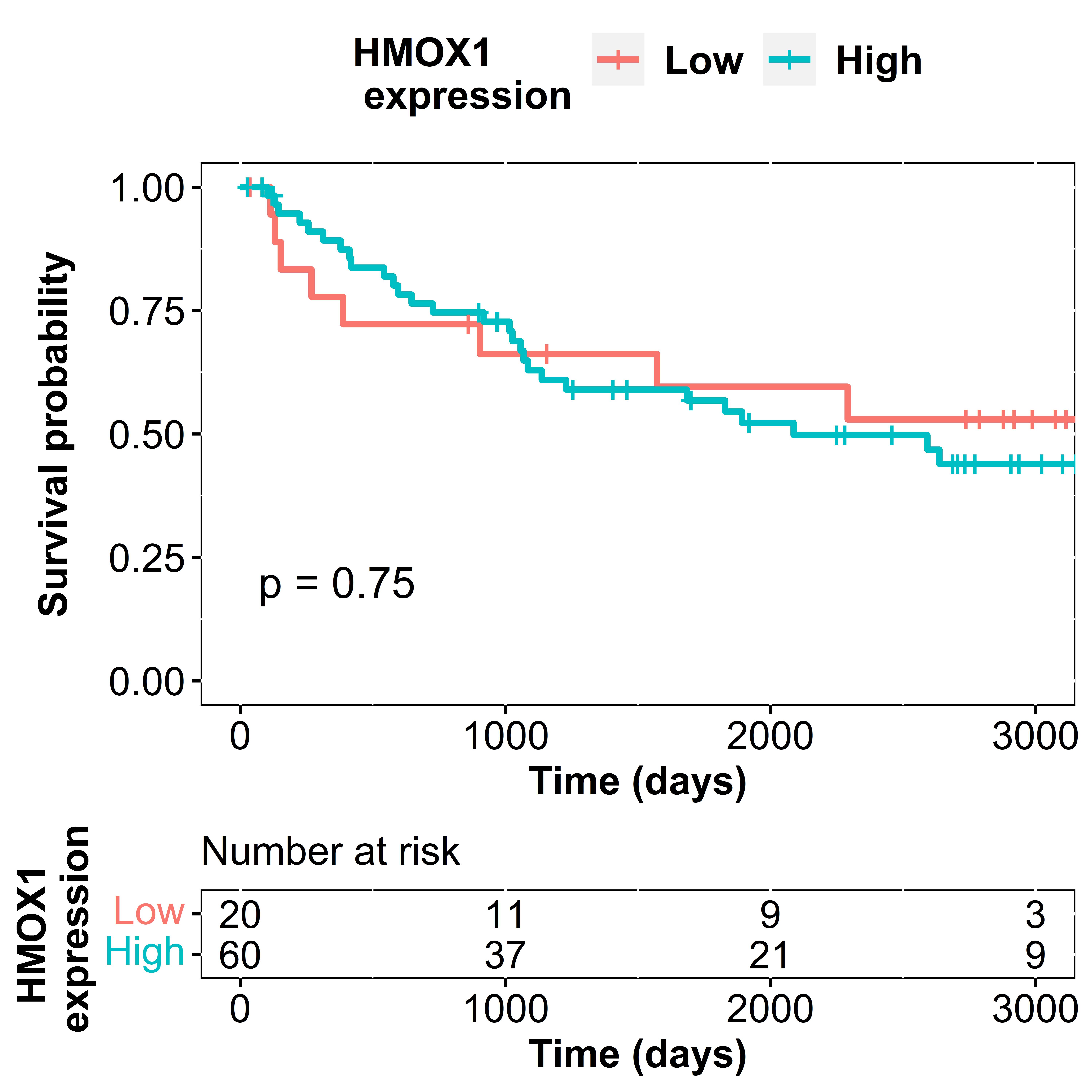

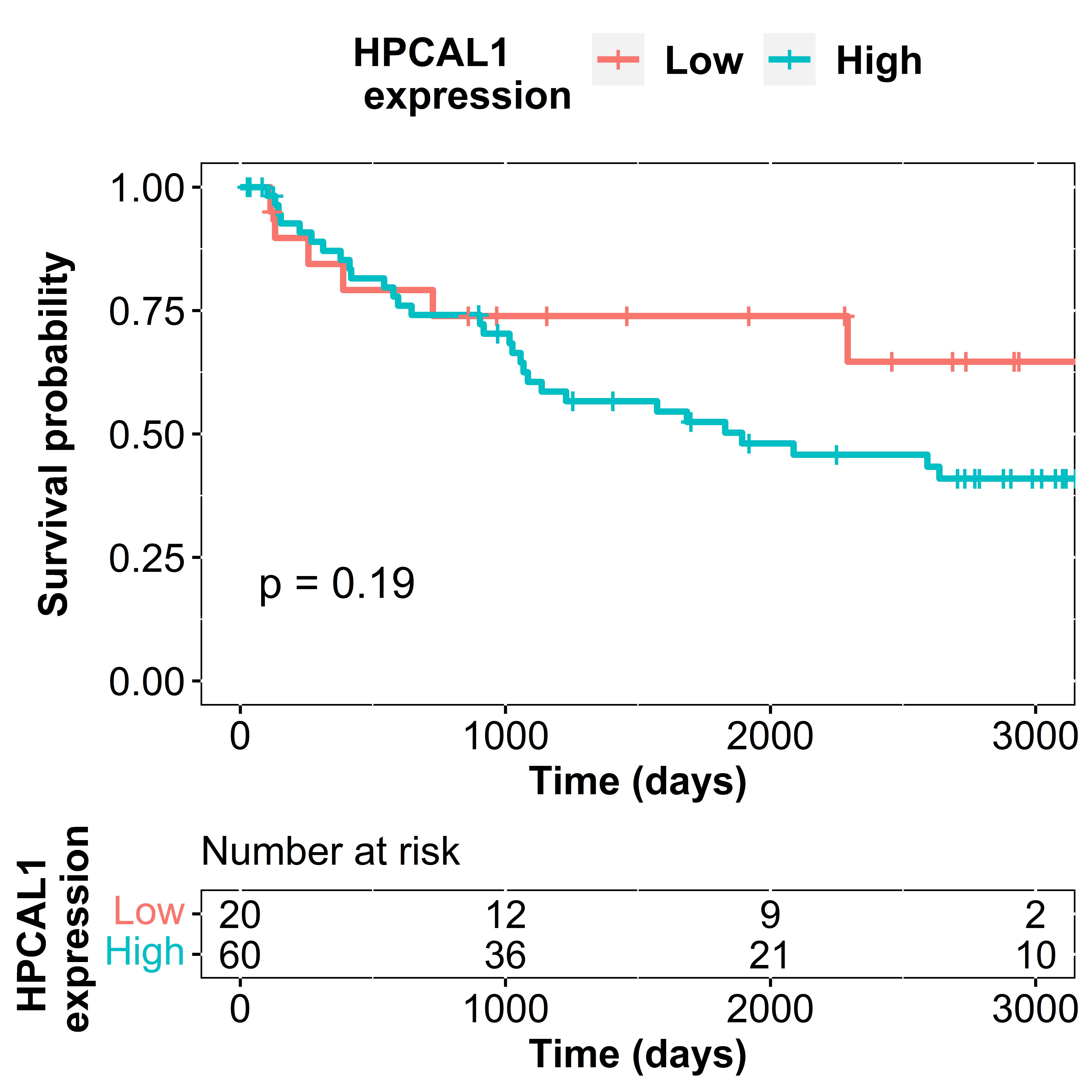

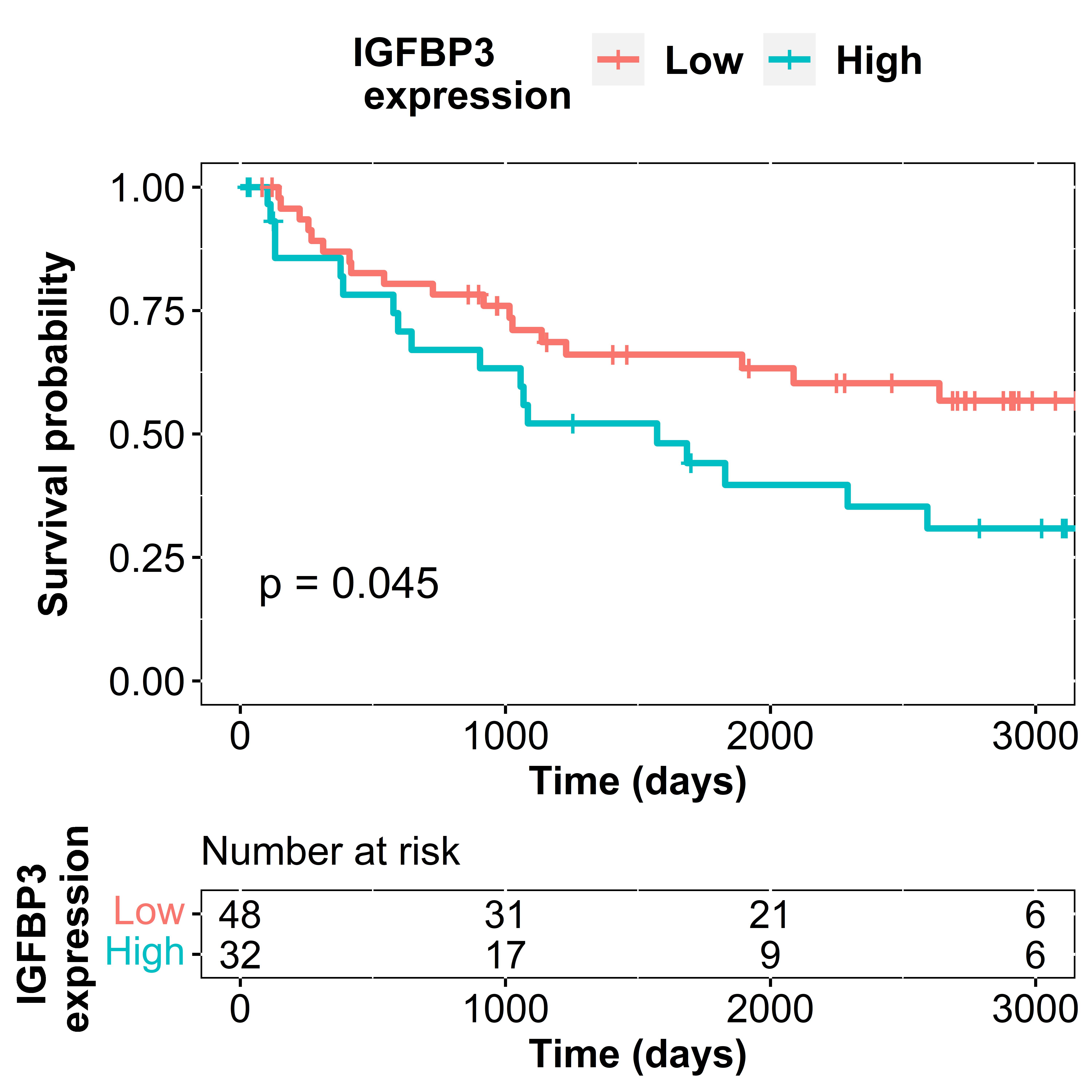

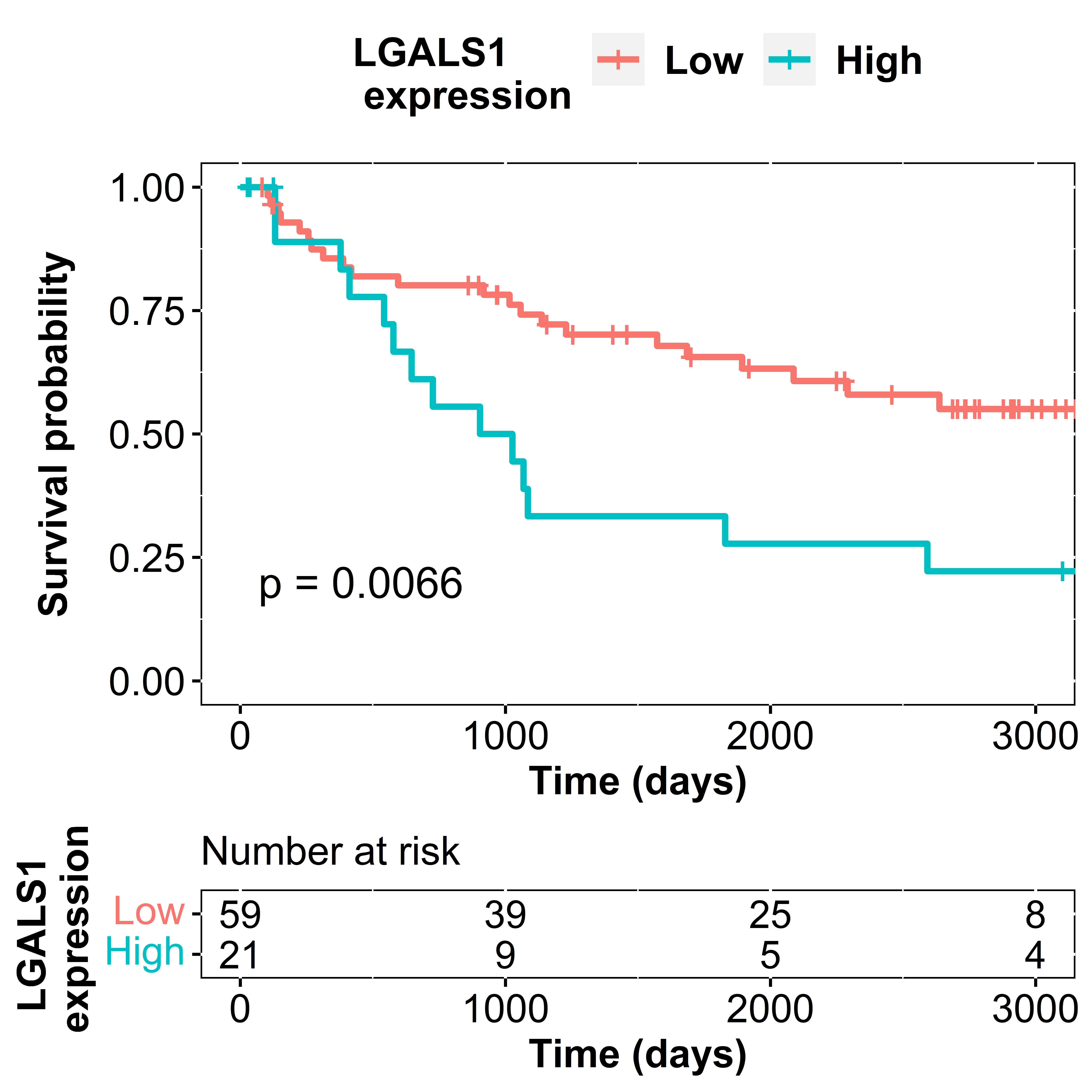

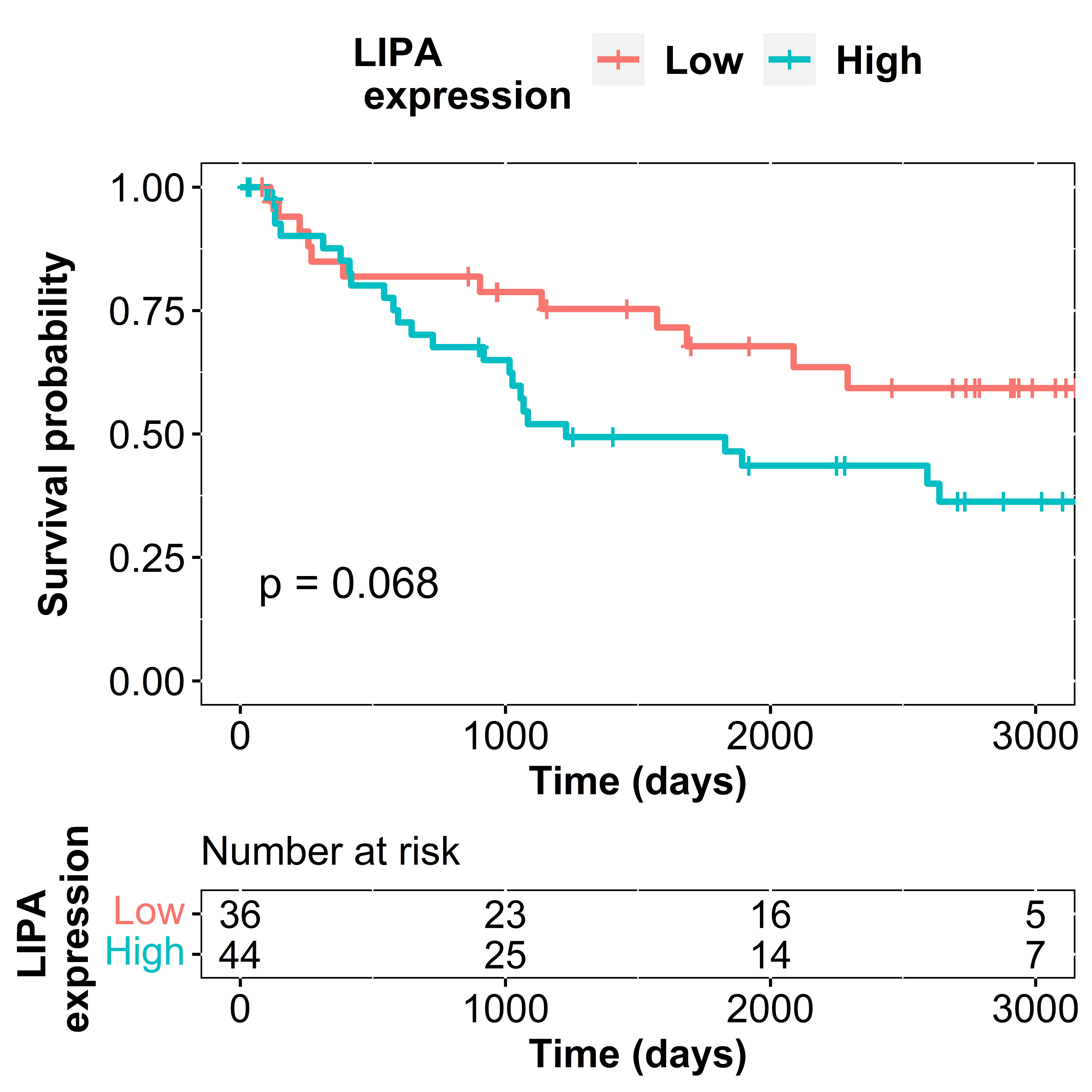

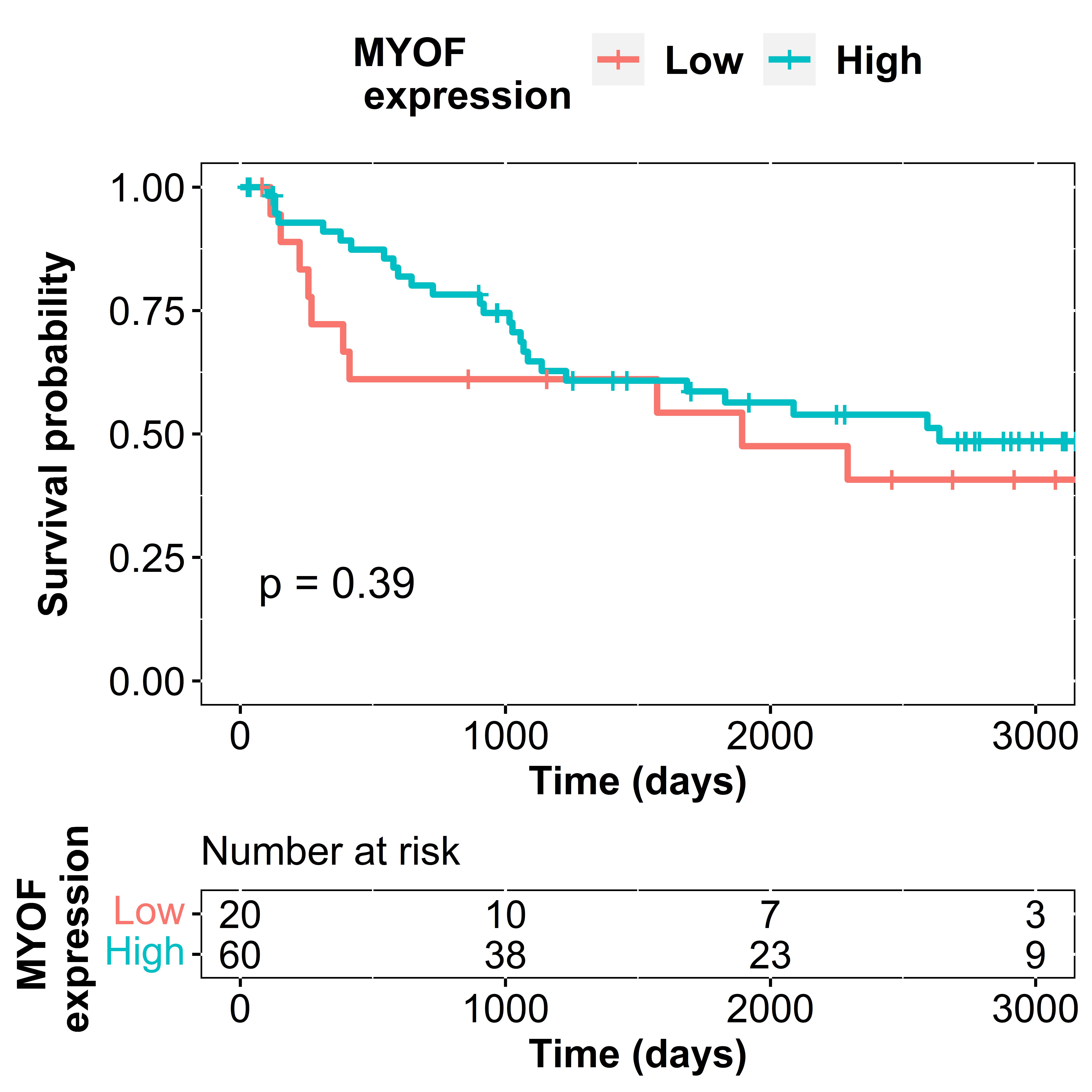

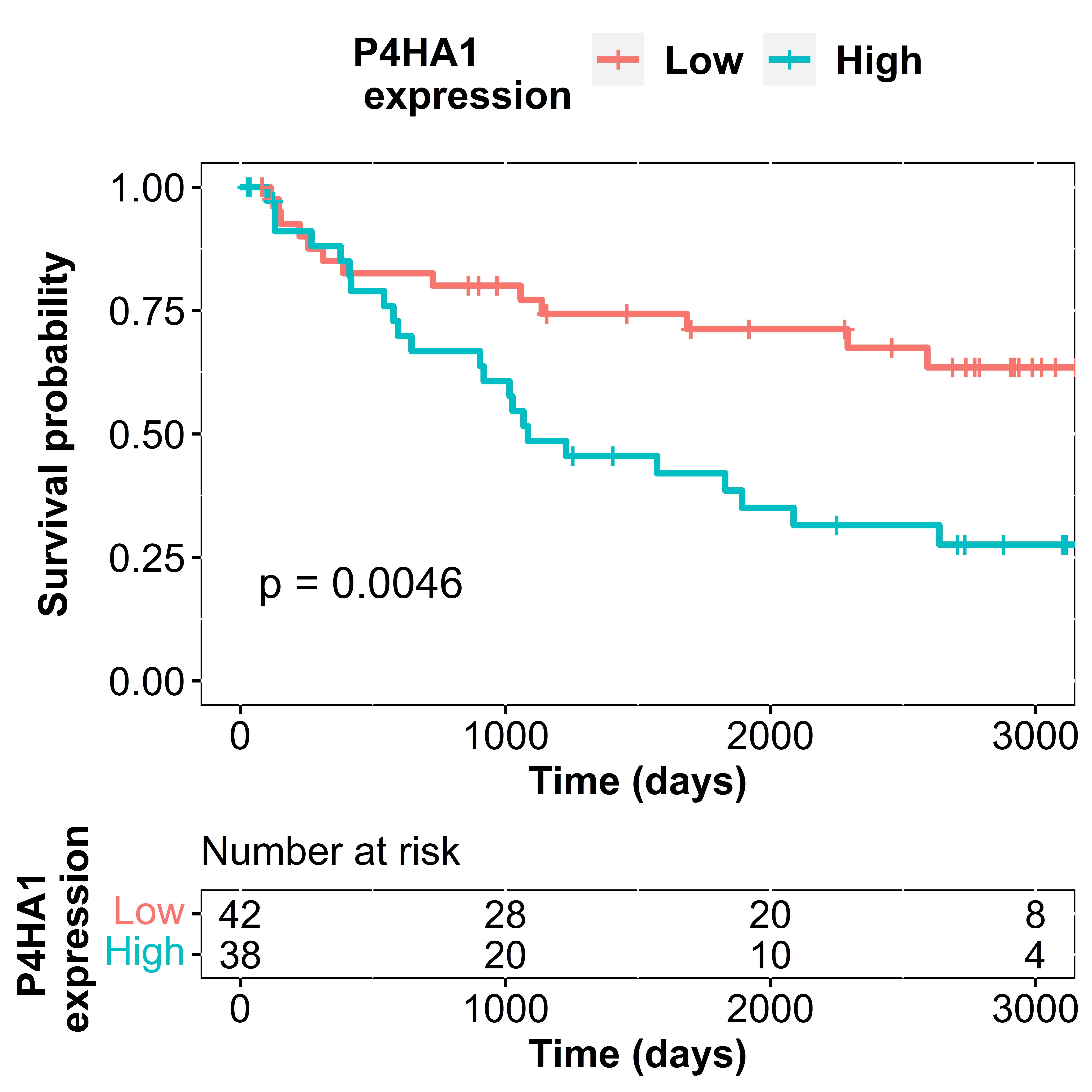

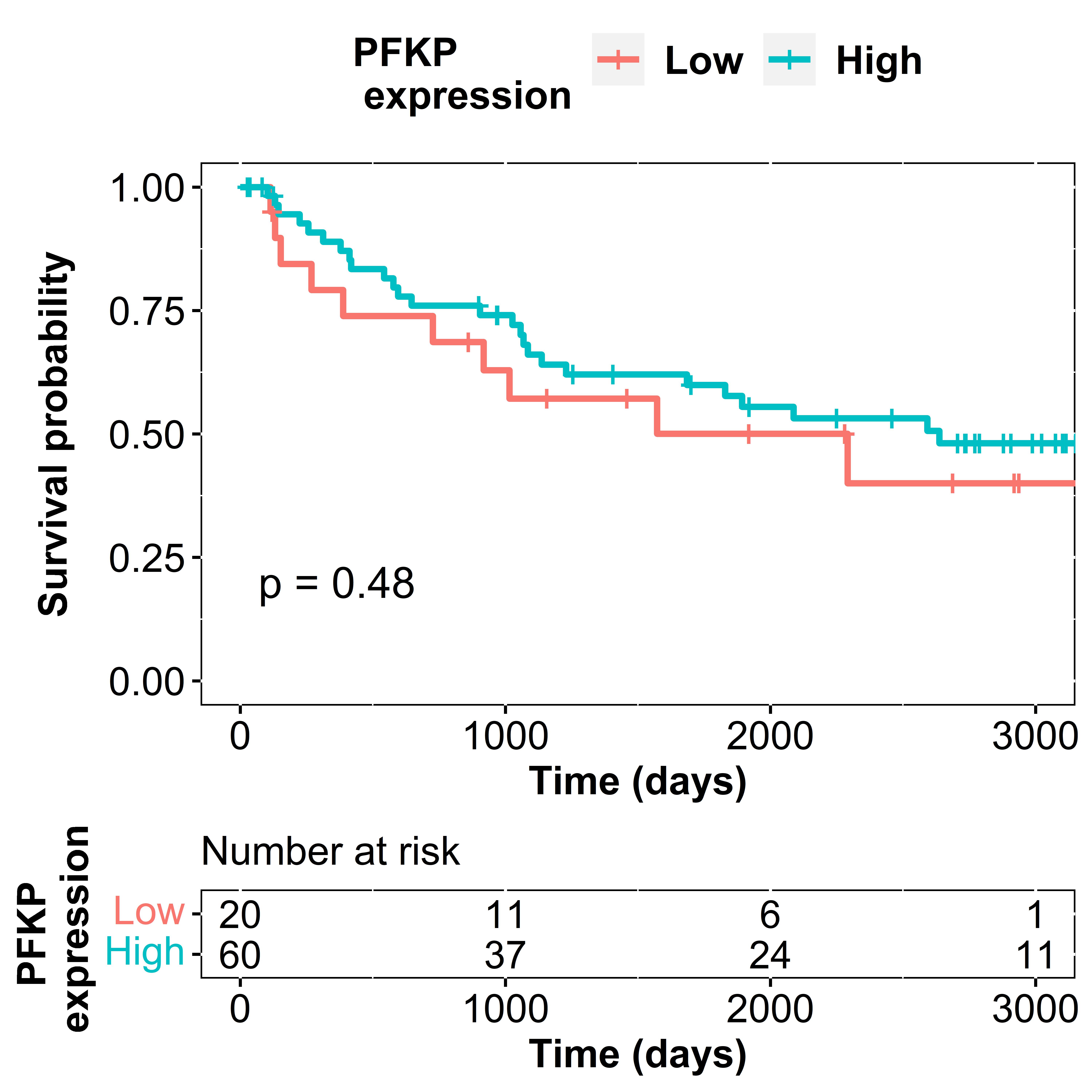

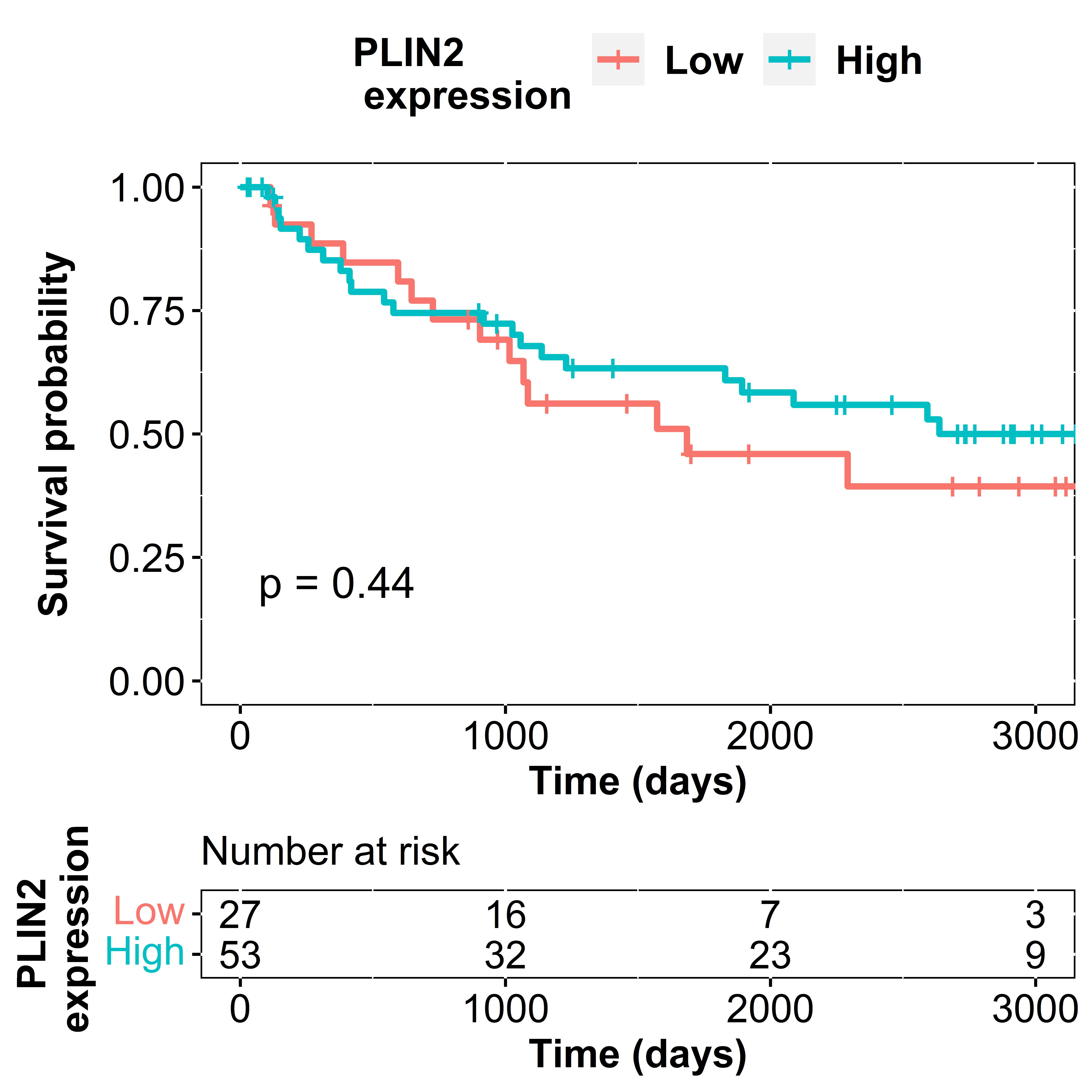

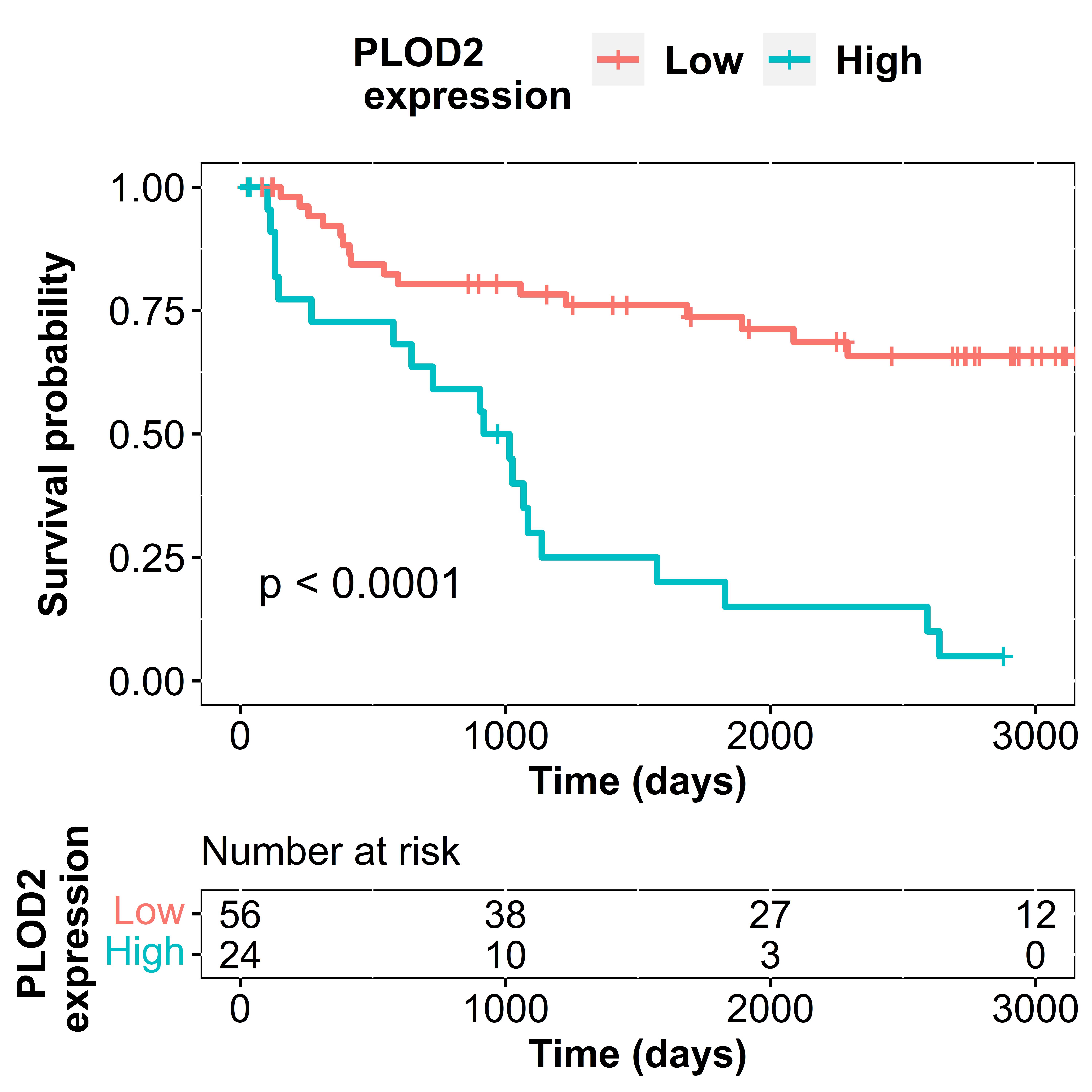

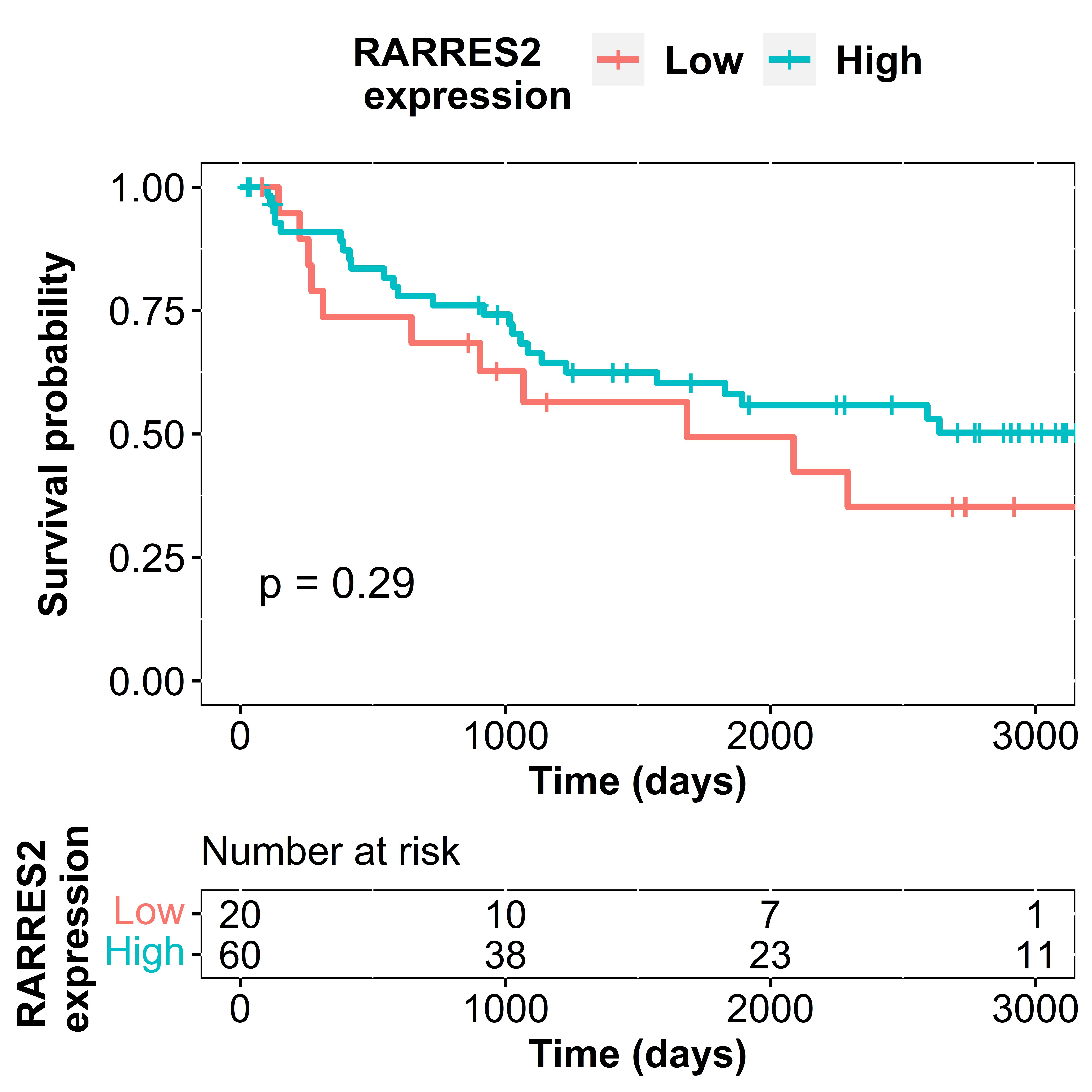

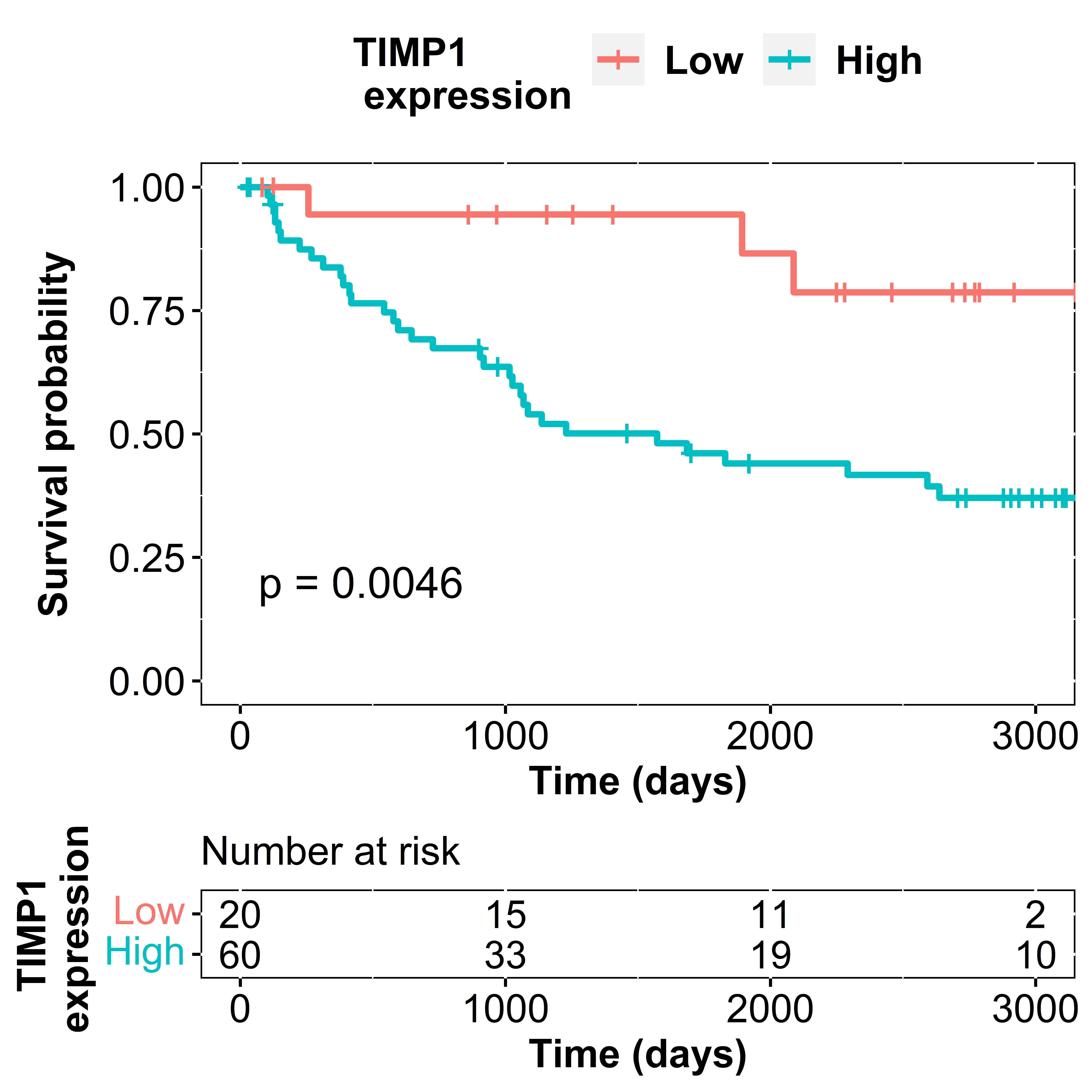

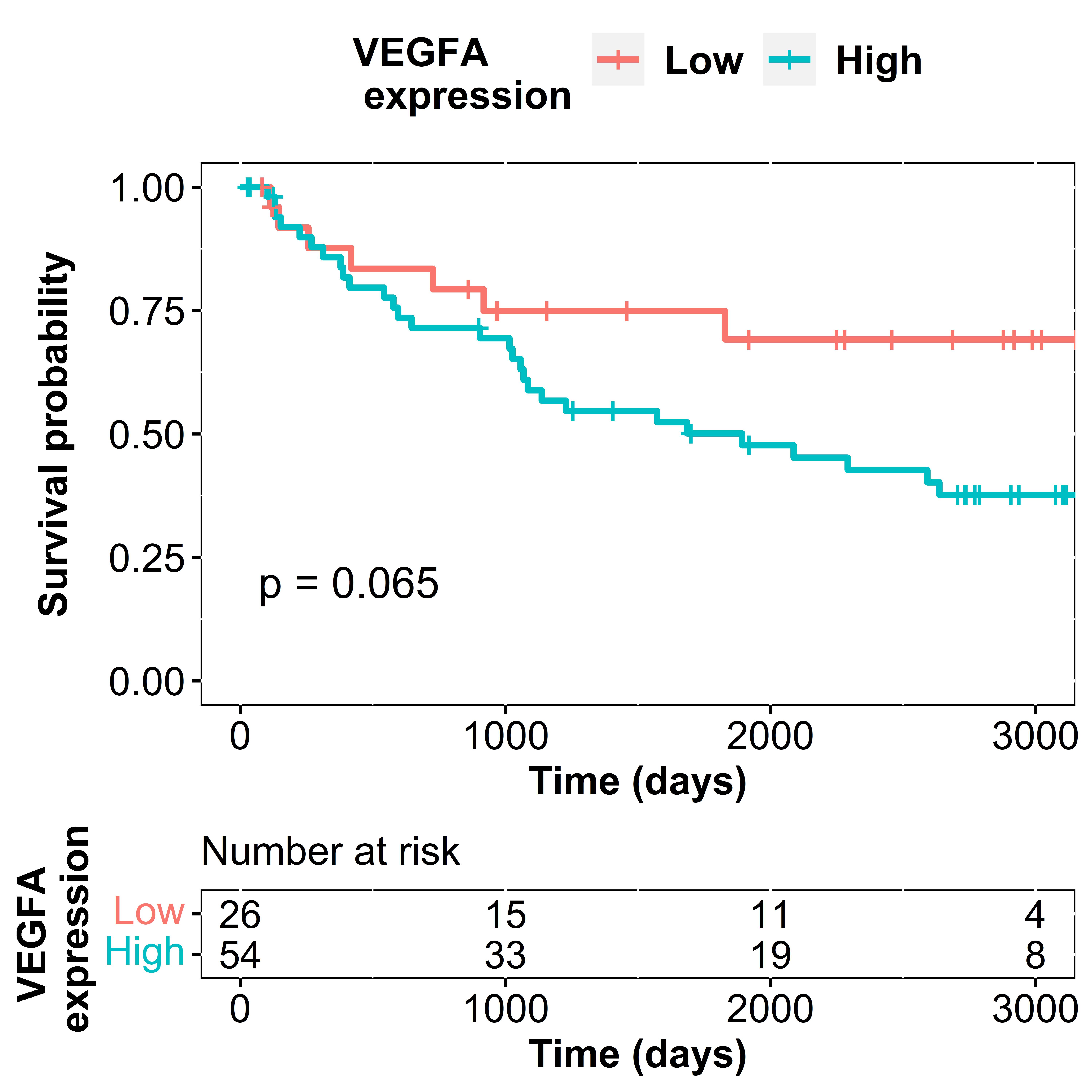

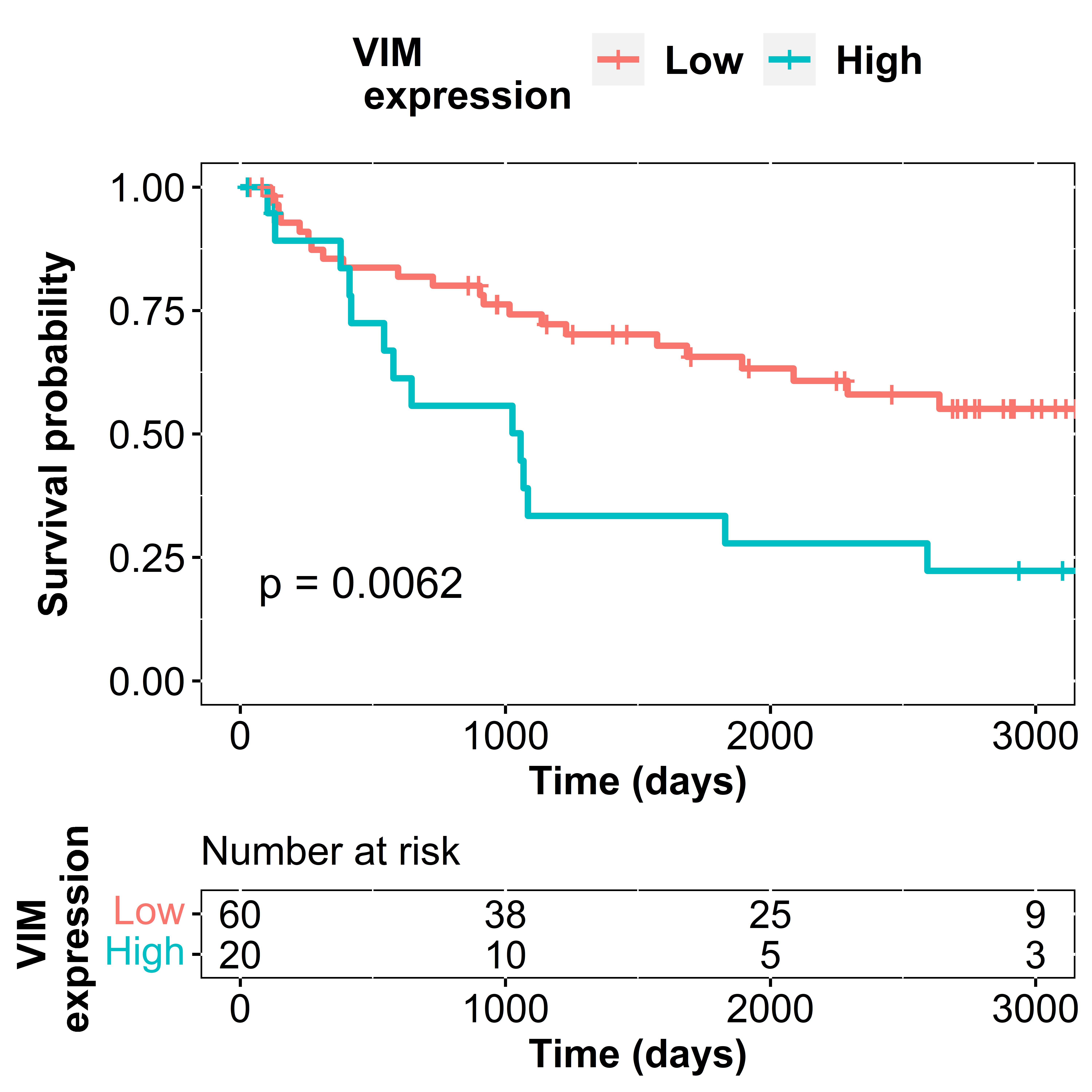

Supplement: Supplementary file 1 [file ijms-24-04488-s001.zip › kmPlots.docx]
